# Ultrasound promoted green synthesis, anticancer evaluation, and molecular docking studies of hydrazines: a pilot trial

**DOI:** 10.1080/14756366.2021.1995727

**Published:** 2021-12-11

**Authors:** Amena Ali, Abuzer Ali, Abu Tahir, Mohammed Afroz Bakht, Mohamed Jawed Ahsan

**Affiliations:** a Department of Pharmaceutical Chemistry, College of Pharmacy, Taif University, Taif, Saudi Arabia; b Department of Pharmacognosy, College of Pharmacy, Taif University, Taif, Saudi Arabia; c Department of Pharmacology, Raghukul College of Pharmacy, Bhopal, India; d Department of Chemistry, College of Science and Humanity Studies, Prince Sattam Bin Abdulaziz University, Al-Kharj, Saudi Arabia; e Department of Pharmaceutical Chemistry, Maharishi Arvind College of Pharmacy, Jaipur, India

**Keywords:** Anticancer, hydrazine carboxamide, EGFR inhibitor, ultrasound, water: glycerol system

## Abstract

We reported herein an efficient, environmentally friendly synthesis of hydrazine carboxamides (**6a–l**) in a water-glycerol (6:4) solvent system using ultrasonic irradiation. Ultrasonicated reactions were found to be much faster and more productive than conventional synthesis. The prepared compounds (**6a–l**) were tested against nine panels of 60 cancer cell lines according to the National Cancer Institute (NCI US) protocol. *N*-(4-Chlorophenyl)-2-(2-oxoindolin-3-ylidene)hydrazine-1-carboxamide (**6b**) was discovered to be promising anticancer agents with higher sensitivity against CCRF-CEM, HOP-92, UO-31, RMPI-8226, HL-60(TB), and MDA-MB-468 with percent growth inhibitions (%GIs) of 143.44, 33.46, 33.21, 33.09, 29.81, and 29.55 respectively. Compounds (**6a–l**) tested showed greater anticancer activity than Imatinib, except for compound **6k**. Compounds **6b** and **6c** were found to be lethal on the CCRF-CEM leukaemia cell line, with %GIs of 143.44 and 108.91, respectively. Furthermore, molecular docking analysis was performed to investigate ligand binding affinity at the active site of epidermal growth factor (EGFR).

## 1. Introduction

Hydrazine carboxamides have a wide range of biological activities, including anticancer activity^1–6^. Hydrazine carboxamides have been extensively studied for a variety of biological activities such as anticonvulsant^7–9^, antimicrobial^10^, anti-HIV^11^^,^^12^, radioprotectors^13^, antitubercular^14^, antitrypanosomal^15^^,^^16^, and many others. They have also been used as kinase inhibitors, inhibiting EGFR, VEGFR, CDK2, CDK5, GSK3, and many others^4–6^. Various methods for preparing hydrazine carboxamides via conventional heating have been reported. The semicarbazide and aromatic carbonyl compound were heated for 1 to 48 h in ethanol with a few drops of glacial acetic acid^8^. Another method of preparation, involving stirring and heating, took 30 min to complete the reaction^9^^,^^17^. The use of ultrasonication in green synthesis is an important method for the synthesis of organic compounds. It is an environmentally friendly method for producing higher yields of medicinal compounds. Ultrasonication is widely used in the food and meat processing industries^18^. It has also been used in the alcoholic beverage and beverage industries^19–21^. Ultrasonication has also been used to extract active ingredients from crude natural compounds^22^. Ultrasonication has also been used in the synthesis of medicinal compounds^23^. Over the last few decades, tremendous progress has been made in the use of ultrasound technology in organic and material synthesis^24^^,^^25^. The ultrasound technique increased the reaction rate even under milder conditions when compared to traditional heating methods^26^^,^^27^. Ultrasonic heating is not only more energy-efficient than traditional heating methods, but it is also less expensive^28^. During chemical reactions, ultrasound causes acoustic cavitation^27^^,^^29^. Acoustic cavitation generates high pressure (18,000 atomic pressures) and temperature (2000–5000 K), which affects chemical transformations^29–31^. The use of ultrasonication in the preparation of hydrazine carboxamides was reported here as an efficient green method. Researchers are working hard to find sustainable reaction solvents, with water and other benign organic solvents like glycerol gaining attention in recent years^32–34^. In the current study, an ultrasound-accelerated efficient synthesis of hydrazine carboxamide analogues (**6a–l**) in the water-glycerol (6:4) solvent system was performed in good yields. NMR, mass and infra-red spectral data were used to confirm the prepared compounds. The anticancer evaluation was carried out on nine different panels of cancer cell lines. Molecular docking against the epidermal growth factor receptor (EGFR) was also performed as a potential mechanism of action of the target compounds.

**Figure 1.**
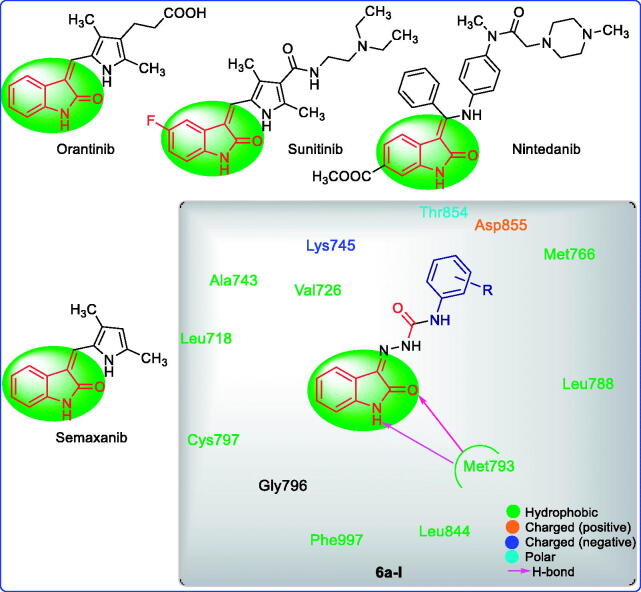
Some of the isatin containing anticancer agents, target compounds (**6a–l**), and their interactions with EGFR (PDB ID: 3W2R).

Cancer is now one of the most dreadful diseases and the second leading cause of death after cardiovascular disease. In 2018, an estimated 9.6 million deaths and 18.1 million cancer cases were reported^35^. EGFR is a popular target for anti-cancer drugs such as Gefitinib, Erlotinib, Cetuximab, Panitumumab, and others^36–41^. Some of the isatin containing anticancer agents like Ninetedanib (multi-kinase inhibitor), Orantinib (multi-kinase inhibitor), Sunitinib (multi-targeted receptor tyrosine kinase inhibitor), and Semaxanib (inhibit ATP binding to the tyrosine kinase domain of vascular endothelial growth factor receptor 2) and the interaction of isatin containing target compounds (**6a–l**) are shown in Figure 1.^42^ The binding interaction of target compounds (**6a–l**) at the EGFR active site was examined and investigated using molecular docking simulation.

## 2. Experimental

### 2.1. General method of synthesis of hydrazine carboxamides (6a–l)

1*H*-Indole-2,3-dione (0.001 mol; 0.147 g) (**5**) and *N*-(substituted phenyl)hydrazine carboxamides (**4a–l**) (0.001 mol) were ultrasonicated at 130 W for 5–20 min in a water-glycerol (6:4) solvent. Once the reactants were consumed, the crude product (precipitate) (**6a–l**) was collected using vacuum filtration. The isolated crude product was re-crystallized using absolute ethanol.

### 2.2. *In vitro* anticancer activity

In a single dose assay, the target compounds (**6a–l**) were tested for anticancer activity against nine different panels of 60 cancer cell lines. The National Cancer Institute (NCI US) protocol was followed to test the anticancer activity at a concentration of 10 µM^43–46^.

### 2.3. Molecular docking studies

The compounds **6a–l** were subjected to a molecular docking simulation against the epidermal growth factor receptor (EGFR). The protein data bank provided the EGFR (PDB: 3W2R) X-ray crystal structure with a resolution of 2.05 Å; *R*-value 0.220 (observed)^47^. The ligands (**6a–l**) were saved as mol files, and docking was carried out according to the protocol described elsewhere^37^.

## 3. Results and discussion

### 3.1. Chemistry

**Scheme 1.**
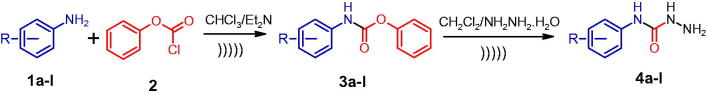
Synthesis of *N*-[substituted phenyl]hydrazinecarboxamide (**4a–l**) via ultrasonic irradiation.

**Scheme 2.**
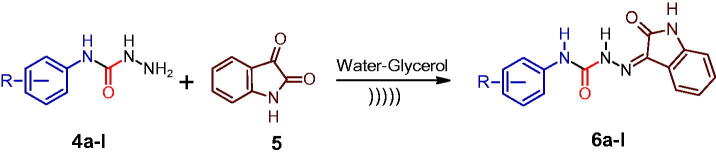
Synthesis of hydrazine carboxamides (**6a–l**) via ultrasonic irradiation.

Phenyl[substituted phenyl]carbamates (**3a–l**) were synthesised by ultrasonication of an equimolar mixture of substituted anilines (**1a–l**) (1 mmol) in triethylamine and phenylchloroformate (**2**) in chloroform. The conventional method took 4 h to complete the same type of reactions, whereas ultrasound-mediated synthesis took only 20 min^8^^,^^38^. In the second step, an equimolar mixture of phenyl[substituted phenyl]carbamates (**3a–l**) and hydrazine hydrate in methylene was allowed to react ultrasonically to produce *N*-[substituted phenyl]hydrazinecarboxamide (**4a–l**). The conventional method took 24 h to complete the same type of reactions, whereas ultrasound-mediated synthesis took 30–45 min^8^^,^^38^. Scheme 1 summarises the outline for the synthesis of *N*-[substituted phenyl]hydrazinecarboxamide (**4a–l**). To obtain the target compounds (**6a–l**), a mixture of *N*-[substituted phenyl]hydrazine carboxamide (**4a–l**) reacted with 1*H*-Indole-2,3-dione (**5**) in water-glycerol (6:4) was ultrasonicated (20 KHz; 130 W) for 5–20 min (Scheme 2). The conventional method took 30 min to 48 h to complete the reaction the same reaction^8^^,^^9^.

### 3.2. Optimisation of reaction conditions

The reaction conditions were initially optimised for the target compound (**6a**). As shown in Table 1, a mixture of *N*-[4-fluorophenyl]hydrazinecarboxamide (1 mmol; 0.169 g) (**4a**) and 1*H*-Indole-2,3-dione (1 mmol; 0.147 g) (**5**) was subjected to various reaction conditions to optimise the reaction conditions and obtain the target compounds (**6a**). The yield was satisfactory, but the conventional method of synthesis required a lengthy process (entries 1 and 2), as shown in Table 1. The yield was increased (to 72%) by stirring the reaction mixture for 60 min at 40 °C (slight heating) in a water-glycerol (6:4) solvent system. The reaction was then irradiated with different solvent systems using sonication. The yields were found to be very low in the case of solvents, dioxane (46%; entry 6) and toluene (55%; entry 7) with irradiative sonication. The yields were found to be satisfactory with the solvents methanol (68%; entry 4), acetonitrile (69%; entry 8), and ethanol (70%; entry 5) with irradiative sonication. The reactions were further ultrasonicated with water-glycerol solvent systems in different proportions and the best result (yield 94%) was obtained with the water-glycerol system in 6:4 or 3:2 proportion (entry 11). When compared to the conventional stirring process (entry 3; yield 72%) under similar solvent system conditions (water-glycerol; 6:4), the yield was higher and the reaction was found to be faster under ultrasonication (entry 11; yield 94%). Finally, all the target compounds (**6a–l**) were synthesised by two different methods, one by conventional stirring on a magnetic stirrer at 40 °C and another with ultrasonic irradiation. The reactions were found to be very fast (5 min), with higher yields (90–94%) of target compounds for phenyls with electronegative substitutions (4-F, 4-Cl, 4-Br, 2-Cl, and 3-Cl-4-F). The physical constants and yields of the target compounds (**6a–l**) are shown in Table 2.

**Table 1.** Optimisation of reaction conditions for the synthesis of *N*-(4-fluorophenyl)-2–(2-oxo-1,2-dihydro-3*H*-indol-3-ylidene)hydrazinecarboxamide (**6a**).

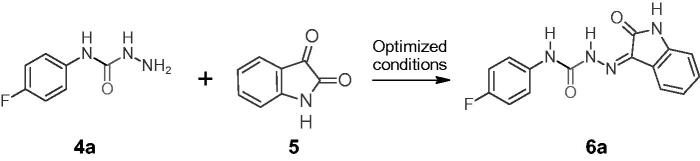				
Entry	Condition^a^	Solvent	Reaction time	Yield^b^ (%)
1	Reflux	CH_3_OH + a drop of GAA	12 h	62
2	Reflux	C_2_H_5_OH + a drop of GAA	10 h	65
3	Stirring at 40 ºC	H_2_O: Glycerol (6:4)	60 min	72
4	Ultrasound	CH_3_OH	20 min	68
5	Ultrasound	C_2_H_5_OH	20 min	70
6	Ultrasound	Toluene	20 min	46
7	Ultrasound	Dioxane	20 min	55
8	Ultrasound	CH_3_CN	20 min	39
9	Ultrasound	H_2_O: Glycerol (8:2)	5 min	72
10	Ultrasound	H_2_O: Glycerol (5:5)	5 min	79
11	Ultrasound	H_2_O: Glycerol (6:4)	5 min	94
12	Ultrasound	H_2_O: Glycerol (7:3)	5 min	85

^a^Reaction condition: 1*H*-Indole-2,3-dione (0.001 mol; 0.147 g) and *N*-(4-fluorophenyl)hydrazinecarboxamide (**4a**) (0.001 mol; 0.169 g).

^b^Yield of final dried compounds.

**Table 2.** Physical constants and yields of the prepared hydrazine carboxamide analogues (**6a–l**).

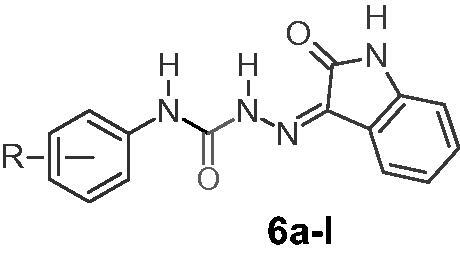						
S. No.	Compound	*R*	Mp (ºC)	*R*_f_*	Yield^a^ (Time in min)	
					Stirring at 40 ºC	))))))^b^
1	**6a**	4-F	220–222	0.68	77% (30 min)	94% (5 min)
2	**6b**	4-Cl	214–216	0.72	72% (25 min)	92% (5 min)
3	**6c**	4-Br	218–220	0.70	68% (25 min)	90% (5 min)
4	**6d**	4-CF_3_	192–194	0.68	66% (20 min)	88% (5 min)
5	**6e**	4-CH_3_	180–182	0.72	65% (40 min)	70% (10 min)
6	**6f**	4-OCH_3_	140–142	0.88	54% (45 min)	68% (15 min)
7	**6g**	2-Cl	130–132	0.70	70% (30 min)	91% (5 min)
8	**6h**	2-CH_3_	120–122	0.66	62% (45 min)	66% (10 min)
9	**6i**	2-OCH_3_	204–206	0.68	56% (30 min)	67% (15 min)
10	**6j**	2,4-(CH_3_)_2_	198–200	0.82	60% (180 min)	72% (20 min)
11	**6k**	2,6-(CH_3_)_2_	190–192	0.86	66% (180 min)	74% (20 min)
12	**6l**	3-Cl-4-F	128–130	0.77	72% (30 min)	90% (5 min)

*Chloroform : methanol (9:1).

^a^Yield of final dried compounds.

^b^Reaction condition: *N*-(Substituted phenyl)hydrazinecarboxamide (**4a–l**) (0.001 mol) and 1*H*-indole-2,3-dione (**5**) (0.001 mol; 0.147 g); Solvent 10 ml [H_2_O : Glycerol (6:4)]; ))))) (Ultrasound) 20 KHz; 130 W.

### 3.3. In vitro anticancer activity

**Figure 2.**
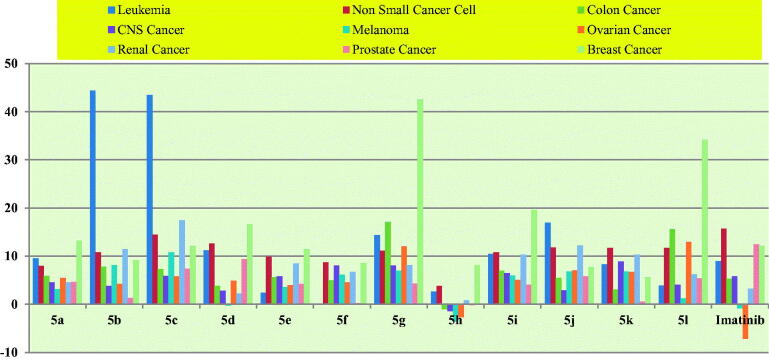
The average %GIs of hydrazine carboxamide analogues (**6a–l**) and Imatinib at 10 µM.

According to NCI US protocols, *in vitro* anticancer action of the target compounds was carried out against nine separate panels of 60 cancer cell lines^43–46^. The results of anticancer screening against the six most susceptible cancer cell lines are given Table 3, whereas detailed anticancer results on 60 cancer cell lines are given in Table 1S (Supplementary Information). The anticancer activity was expressed as growth percent (GP) and percent growth inhibition (% GI). Compounds, **6i**, **6g**, **6d,** and **6e** showed maximum sensitivity against the UO-31 (renal cancer) cell line with %GIs of 41.32, 35.00, 34.95, and 28.55% respectively. The compounds, **6a**, **6f**, **6j** and **6k** showed maximum sensitivity against T-47D (%GI = 33.86), HL-60(TB) (%GI = 64.73), HOP-92 (%GI = 41.77), MCF7 (%GI = 14.94) respectively. In contrast to the standard drug Imatinib, the mean growth percentages (GPs) of all target compounds (except compound 6k) be promising. The anticancer data of Imatinib was retrieved from the NCI database with NSC code 759854^43^. The compound **6l** showed maximum sensitivity against MCF7, MDA-MB-468, T-47D, KM12, HCT-15, and HOP-92 with % GI values of 75.92, 66.01, 52.99, 45.66, 41.78, and 36.62 respectively. The compound **6c** showed maximum sensitivity against CCRF-CEM, HL-60(TB), RMPI-8226, UO-31, NCI-H322M, and UACC-62 with %GI values of 108.91, 61.19, 43.88, 30.75, 24.70, and 24.28 respectively. The compound **6h** showed maximum sensitivity against MDA-MB-468, MCF7, T-47D, KM12, UO-31, and HCT-15 with %GI values of 88.54, 80.17, 57.99, 46.45, 31.95, and 31.95 respectively. *N*-(4-Chlorophenyl)-2–(2-oxoindolin-3-ylidene)hydrazine-1-carboxamide (**6b**) showed the most promising anticancer activity with a mean GP of 85.97 and was found to be maximum sensitive against CCRF-CEM, HOP-92, UO-31, RMPI-8223, HL-60(TB), and MDA-MB-468 with %GI values of 143.44, 33.46, 33.21, 33.09, 29.81, and 29.55 respectively. The compounds, **6b** and **6c** showed the lethal effect on CCFR-CEM (leukaemia) cell lines with a %GI value of 143.44 and 108.91 respectively. The average percent growth inhibitions (%GIs) of the target compounds were calculated for each compound and are shown in Table 4 and Figure 2. The compound, **6b** showed promising results on panels of leukaemia, melanoma, and renal cancer cell lines, while the compound **6l** showed promising results against panels of ovarian and breast cancer cell lines. The compounds, **6g** and **6k** showed promising anticancer activity on panels of colon and CNS cancer cell lines respectively. Furthermore, sunitinib showed anticancer activity against renal cancer cell line (786-0) with effective dose, ED_20_, ED_50_, ED_70_ and ED_90_ values of 3.6, 20.7, 45.2, and 90.5 µM respectively, whereas the title compounds (**6a–l**) showed anticancer activity with % GI values ranging from 10.39 to −2.47% at 10 µM^48^.

**Table 3.** The GP and %GI of hydrazine carboxamides (**6a–l**) at 10 µM.

Compound/NSC Code	Assay of cancer cell lines in one dose assay at 10 µM				
	Mean GP	Range of GP	The most sensitive cell lines	GP	% GI^#^
**6a** **NSC** 803846	93.52	66.16 to 112.67	T-47D (Breast cancer)	66.16	33.86
			MCF7 (Breast cancer)	71.29	28.71
			UO-31 (Renal cancer)	72.77	27.23
			NCI-H522 (Non-small cell lung cancer)	78.52	21.48
			UACC62 (Non-small cell lung cancer)	79.31	20.69
			SK-OV-3 (Ovarian cancer)	80.54	19.46
**6b** **NSC** 803848	85.97	−43.84 to 114.71	CCRF-CEM (Leukaemia)	**−43.44^a^**	**143.44**
			HOP-92 (Non-small cell lung cancer)	66.54	33.46
			UO-31 (Renal cancer)	66.79	33.21
			RMPI-8226 (Leukaemia)	66.91	33.09
			HL-60(TB) (Leukaemia)	70.19	29.81
			MDA-MB-468 (Breast cancer)	70.45	29.55
**6c** **NSC** 803847	88.45	−8.91 to 110.49	CCRF-CEM (Leukaemia)	**−8.91** ^a^	**108.91**
			HL-60(TB) (Leukaemia)	**38.91**	**61.19**
			RMPI-8226 (Leukaemia)	56.12	43.88
			UO-31 (Renal cancer)	69.25	30.75
			NCI-H322M (Non-small cell lung cancer)	75.30	24.70
			UACC-62 (Melanoma)	75.72	24.28
**6d** **NSC** 803849	93.56	65.05 to 111.03	UO-31 (Renal cancer)	65.05	34.95
			IGROV1 (Ovarian cancer)	79.02	20.98
			MDA-MB-468 (Breast cancer)	82.76	17.24
			NCI-H322M (Non-small cell lung cancer)	82.80	17.20
			NCI-H226 (Non-small cell lung cancer)	84.23	15.77
			MCF7 (Breast cancer)	84.31	15.69
**6e** **NSC** 803850	94.11	71.45 to 106.76	UO-31 (Renal cancer)	71.45	28.55
			UACC-62 (Melanoma)	79.52	20.48
			IGROV1 (Ovarian cancer)	81.20	18.80
			HCT-116 (Colon cancer)	85.17	14.83
			SNB-19 (CNS cancer)	85.38	14.62
			MALME3M (Melanoma)	85.71	14.29
**6f** **NSC** 803851	91.17	64.50 to 118.02	HL-60(TB) (Leukaemia)	**35.27**	**64.73**
			UO-31 (Renal cancer)	64.50	35.50
			NCI-H322M (Non-small cell lung cancer)	77.15	22.85
			HOP-92 (Non-small cell lung cancer)	77.45	22.55
			A498 (Renal Cancer)	78.08	21.92
			NCI-H522 (Non-small cell lung cancer)	79.94	20.06
**6g** **NSC** 803852	92.39	65.00 to 111.59	UO-31 (Renal cancer)	65.00	35.00
			SNB-75 (CNS cancer)	71.88	28.12
			HOP-92 (Non-small cell lung cancer)	74.84	25.16
			CCRF-CEM (Leukaemia)	74.89	25.11
			UACC62 (Melanoma)	79.06	20.94
			IGROV1 (Ovarian cancer)	79.34	20.66
**6h** **NSC** 803853	86.16	11.46 to 107.15	MDA-MB-468 (Breast cancer)	**11.46**	**88.54**
			MCF7 (Breast cancer)	**19.83**	**80.17**
			T-47D (Breast cancer)	**42.01**	**57.99**
			KM12 (Colon cancer)	53.55	46.45
			UO-31 (Renal cancer)	68.05	31.95
			HCT-15 (Colon cancer)	68.05	31.95
**6i** **NSC** 803854	90.93	58.68 to 117.61	UO-31 (Renal cancer)	58.68	41.32
			MCF7 (Breast cancer)	58.98	41.02
			T-47D (Breast cancer)	68.61	31.39
			CAKI-1 (Renal cancer)	73.32	26.68
			UACC-62 (Melanoma)	79.17	20.83
			HOP-92 (Non-small cell lung cancer)	80.29	19.71
**6j** **NSC** 803856	93.46	58.44 to 128.26	HOP-92 (Non-small cell lung cancer)	58.23	41.77
			T-47D (Breast cancer)	58.44	41.56
			MCF7 (Breast cancer)	67.75	32.25
			UO-31 (Renal cancer)	68.26	31.74
			HL-60(TB) (Leukaemia)	70.18	29.82
			CAKI-1 (Renal cancer)	81.39	18.61
**6k** **NSC** 803857	99.39	75.06 to 122.10	MCF7 (Breast cancer)	75.06	14.94
			UO-31 (Renal cancer)	81.67	18.33
			NCI-H522 (Non-small cell lung cancer)	84.35	15.65
			CAKI-1 (Renal cancer)	88.19	11.81
			HOP-92 (Non-small cell lung cancer)	89.16	10.84
			UACC-62 (Melanoma)	90.87	9.13
**6l** **NSC** 803858	89.53	81.12 to 118.88	MCF7 (Breast cancer)	**24.08**	**75.92**
			MDA-MB-468 (Breast cancer)	**33.99**	**66.01**
			T-47D (Breast cancer)	**47.01**	**52.99**
			KM12 (Colon cancer)	54.34	45.66
			HCT-15 (Colon cancer)	58.22	41.78
			HOP-92 (Non-small cell lung cancer)	63.38	36.62
Imatinib*^^NSC 759854	94.56	52.9 to 122.8	HT29 (Colon cancer)	52.9	47.1
			HOP-92 (Non-small cell lung cancer)	56.3	43.7
			MDA-MB-468 (Breast cancer)	70.9	29.1
			SF-539 (CNS cancer)	75.5	24.5
			SK-MEL-5 (Melanoma)	77.7	22.3

^a^The tested compound has a lethal effect on cancer cell lines.

**^#^**The percent growth inhibition (%GI) was calculated as % GI=100−GP.

*The data of Imatinib was retrieved from the NCI database with NSC Code 759854^43^.

**Table 4.** The average %GIs of hydrazine carboxamides (**6a–l**) and Imatinib at 10 µM.

Panels	6a	6b	6c	6d	6e	6f	6g	6h	6i	6j	6k	6l	Imatinib*
Leukaemia	9.56	43.47	**44.35**	11.24	2.44	−0.13	14.41	2.61	10.50	16.91	8.29	3.92	9
Non-small cancer cell	7.97	14.45	10.81	12.63	9.85	8.71	11.10	3.80	10.76	11.79	11.74	11.67	**15.68**
Colon cancer	5.92	7.28	7.83	3.81	5.62	4.97	**17.08**	−1.04	6.93	5.46	3.08	15.63	5.34
CNS cancer	4.53	5.87	3.85	2.79	5.79	8.06	8.02	−1.45	6.47	2.92	**8.92**	4.07	5.8
Melanoma	3.14	**10.83**	8.11	−0.26	3.60	6.16	6.93	−3.61	5.98	6.81	6.84	1.19	−0.87
Ovarian cancer	5.46	5.79	4.22	4.89	4.01	4.53	12.07	−2.68	5.05	7.04	6.73	**12.94**	−7.16
Renal cancer	4.53	**17.48**	11.46	2.27	8.45	6.71	8.10	0.80	10.29	12.21	10.29	6.23	3.25
Prostate cancer	4.68	7.39	1.33	9.38	4.23	0.22	4.35	−0.16	4.09	5.79	0.54	5.36	**12.5**
Breast cancer	13.21	12.14	9.13	16.62	11.46	8.56	42.58	8.12	19.63	7.76	5.66	**34.12**	12.15

*The data of Imatinib was retrieved from NCI website with NSC Code 759854^43^.

Bold font showed the maximum anticancer activity on the respective cancer panel by the tested compound.

**^#^**The percent growth inhibition (%GI) was calculated as %GI=100−GP.

The structure-activity relationship was established with the anticancer results showed the target compound with 4-chloro substitution on the phenyl ring showed maximum anticancer activity followed by 2-methyl, 4-bromo and 3-chloro-4-fluoro substitution on the phenyl ring. The anticancer activity was found to be associated with substitutions as 4-Cl > 2-CH_3_> 4-Br > 3-Cl-4-F > 2-OCH_3_ > 4-OCH_3_ > 2-Cl > 2,4-(CH_3_)_2_> 4-F > 4-CF_3_> 4-CH_3_> 2,6-(CH_3_)_2_.

### 3.4. Molecular docking

**Figure 3.**
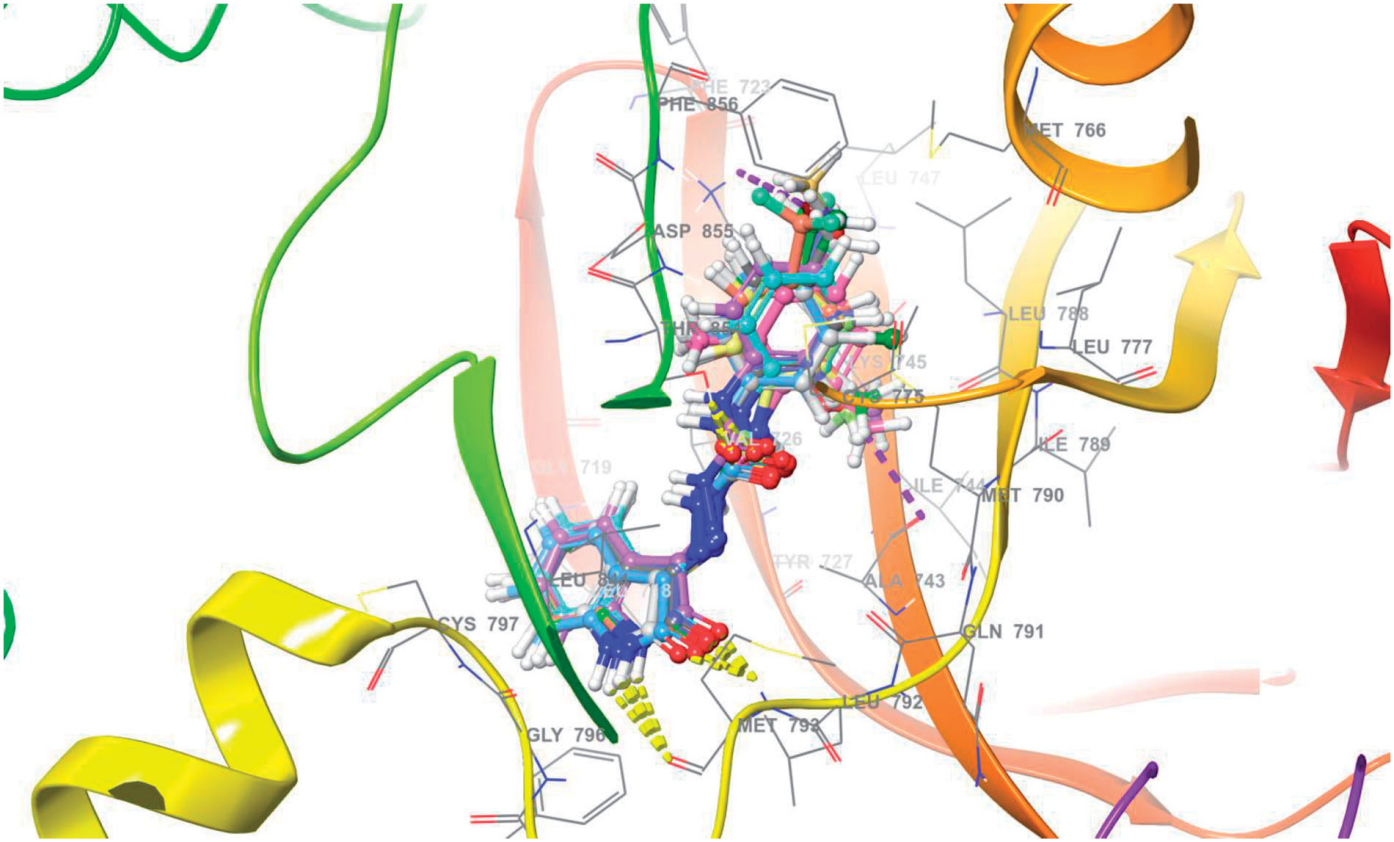
The molecular docking of ligands **6a–l** within the active site of EGFR.

**Figure 4.**
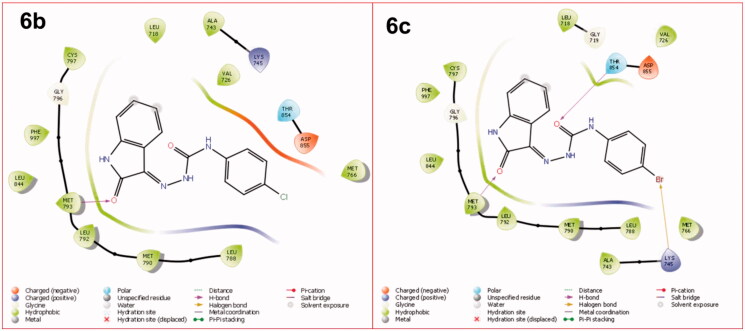
The 2D interaction of the compounds 6b and 6c within the active site of EGFR.

**Figure 5.**
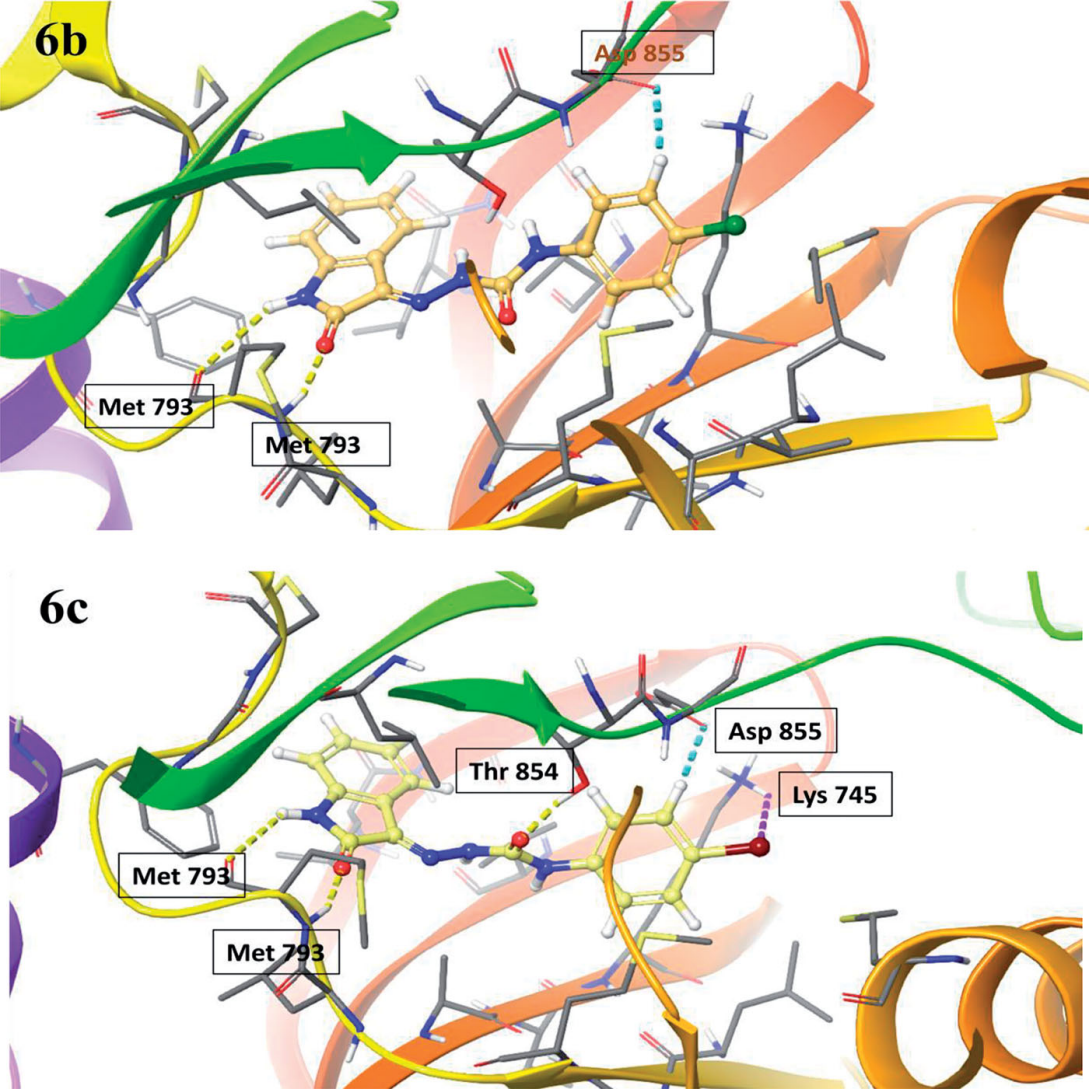
The 3D interaction of the compounds, 6b and 6c within the active site of EGFR.

The molecular docking was assessed to explore the interaction of target ligands (**6a–l**) against EGFR, a potential target for anticancer drugs according to the reported protocol^37^. The target compounds efficiently bind within the hydrophobic domain of EGFR. H-Bond interactions of NH and CO functions of indole ring with residue Met793 were observed in all the target compounds (**6a–l**). An additional H-bond interaction of CO function with the residue Thr854 was observed in the compounds **6c**, **6e**, **6j,** and **6k**. Some of the compounds, **6b**, **6c**, **6i**, **6j**, and **6l** also showed π-π stacking of aminophenyl ring with the residue Asp855. Some of the halogenated compounds (**6c** and **6g**) showed a halogen bond with the residue Lys745. The molecular docking scores and types of interaction with the amino acid residue of EGFR are summarised in Table 5. The molecular docking of ligands **6a–l** within the active site of EGFR is shown in Figure 3. The 2D interactions of compounds **6b** and **6c** against EGFR are shown in Figure 4. The 3D interactions of compounds **6b** and **6c** against EGFR are shown in Figure 5. The 2D and 3D interactions of some of the compounds against EGFR are shown in Figure 1S, 5S, while 3D interaction of Sunitinib, Semaxanib, and Imatinib against EGFR are shown in Figure 6S (Supplementary Information). The docking scores of the title compounds (**6a–l**) ranged from −7.284 to −9.967 kcal/mol, whereas the docking scores of reference drugs, Imatinib, sunitinib, and semaxanib were found to be −7.971, −7.825 and −8.148 kcal/mol respectively. Compound **6b** exhibited promising anticancer activity at 10 µM and was found to have a lethal effect on the leukaemia cell line, CCRF-CEM, exhibiting two types of interaction such as H-bond and π-π-stacking with the important residues Met793, andAsp855 respectively. Such type of interactions was also observed for the compounds **6i** and **6l** that displayed promising anticancer activity against UO-31 and MCF7 cell line with %GI of 41.32 and 75.92. Compounds **6c** and **6j** had similar types of interactions, though compound **6c** had an additional halogen bond interaction with the residue Lys745. Compound **6c** had the most promising anticancer activity against CCRF-CEM (% GI = 108.91), while compound **6j** displayed the most promising activity against HOP 92 (% GI = 108.91) cell lines. In molecular docking studies, Imatinib and Sunitinib showed three types of interactions: H-bond, π-cation, and π-π-staking, whereas Semaxanib showed only H-bond interaction as shown in Figure 6S (Supplementary Information).

**Table 5.** The molecular docking studies of hydrazine carboxamide analogues (**6a–l**) against the active site EGFR.

S. No.	Compound	Docking score	Glide emodel	Types of interaction
1	**6a**	−8.154	−66.255	H-bond (Met793)
2	**6b**	−8.383	−68.635	H-bond (Met793), π-π-Staking (Asp855)
3	**6c**	−8.659	−69.480	H-bond (Met793, Thr854), Halogen bond (Lys745), π-π-Staking (Asp855)
4	**6d**	−9.175	−65.750	H-bond (Met793)
5	**6e**	−7.284	−62.636	H-bond (Met793), H-bond (Thr854)
6	**6f**	−8.246	−70.716	H-bond (Met793)
7	**6g**	−9.332	−74.791	H-bond (Met793), Halogen bond (Ala743)
8	**6h**	−9.118	−76.037	H-bond (Met793)
9	**6i**	−8.875	−75.436	H-bond (Met793), π-π-Staking (Asp855)
10	**6j**	−9.969	−75.640	H-bond (Met793, Thr854), π-π-Staking (Asp855)
11	**6k**	−9.785	−79.712	H-bond (Met793, Thr854)
12	**6l**	−8.621	−67.784	H-bond (Met793), π-π-Staking (Asp855)
13	Imatinib	−7.971	−95.634	H-bond (Asp855, Thr854), π-π-Staking (Met766), π-Cation and π-π-Staking (Asp855, Leu718, and Gly796)
14	Sunitinib	−7.825	−74.018	H-bond (Gly796), π-Cation and π-π-Staking (Asp855)
15	Semaxanib	−8.148	−50.761	H-bond (Gln791)

### 3.5. Toxicity prediction

The title compounds (**6a–l**) were tested for virtual toxicity using the free online software Protox^49^. The 50% lethal dose (LD_50_) of title compounds (**6a–l**) was predicted to be between 2100 and 3009 mg/Kg. The title compounds (**6a–l**) could be classified as Class V compounds based on the predicted value of LD_50_ (>2000 mg/Kg), which meant the compounds would be harmful if swallowed. The results of toxicity prediction are summarised in Table 6. However, testing all of these chemicals on experimental platforms is impossible due to several challenges such as time, cost, and ethical concerns regarding animal trials. As a result, *in silico* toxicity is rapidly evolving as an essential platform for predicting the toxicity of chemicals that may be harmful to humans, animals, plants, and the environment^50^^,^^51^. The title compounds (**6a–l**) were predicted to be free from immunotoxicity, mutagenicity (except for the compounds, **6f**, **6g,** and **6i**), and cytotoxicity, but minor hepatotoxicity and carcinogenicity concerns could not be ruled out.

**Table 6.** The toxicity prediction of hydrazine carboxamide analogues (**6a–l**).

S. No.	Compound	Hepatotoxicity	Carcinogenicity	Immunotoxicity	Mutagenicity	Cytotoxicity	LD_50_ (mg/Kg)
1	**6a**	+	+	−	−	−	2100
2	**6b**	+	+	−	−	−	2100
3	**6c**	+	+	−	−	−	3009
4	**6d**	+	+	−	−	−	2100
5	**6e**	+	+	−	−	−	2100
6	**6f**	+	+	−	+	−	2100
7	**6g**	+	+	−	+	−	2100
8	**6h**	+	+	−	−	−	2100
9	**6i**	+	+	−	+	−	2100
10	**6j**	+	+	−	−	−	2100
11	**6k**	+	+	−	−	−	2100
12	**6l**	+	+	−	−	−	2100

## 4. Conclusion

In the present work, we report herein the green efficient and eco-friendly synthesis of hydrazine carboxamides from *N*-(substituted phenyl)hydrazine carboxamide and 1*H*-indole-2,3-dione in presence of water-glycerol (6:4), a benign, non-toxic, and eco-friendly solvent system under ultrasonication with superior yields. The synthesised compounds were tested for their anticancer activity against nine different panels of 60 cancer cell lines and the results were found to be superior to Imatinib for all the target compounds except compound, **6k**. *N*-(4-Chlorophenyl)-2–(2-oxoindolin-3-ylidene)hydrazine-1-carboxamide (**6b**) was emerged as a lead compound in the study with promising anticancer activity.

## Supplementary Material

Supplemental Material

## References

[1] Pandeya SN. Semicarbazone a versatile therapeutic pharmacophores for fragment based anticonvulsant drug design. Acta Pharm 2012;62:263–86. 23470343 10.2478/v10007-012-0030-1

[2] Ahsan MJ. Semicarbazone analogs as anticonvulsant agents: a review. Cent Nerv Syst Agents Med Chem 2013;13:148–58. 24156314 10.2174/18715249113136660016

[3] Ali SMM, Azad MAK, Jesmin M, et al. In vivo anticancer activity of vanillin semicarbazone. Asian Pac J Trop Biomed 2012;2:438–42. 10.1016/S2221-1691(12)60072-0 PMC3609319 23569946

[4] Liu Z, Wu S, Wang Y, et al. Design, synthesis and biological evaluation of novel thieno[3,2-d]pyrimidine derivatives possessing diaryl semicarbazone scaffolds as potent antitumor agents. Eur J Med Chem 2014;87:782–93. 25440879 10.1016/j.ejmech.2014.10.022

[5] da Cruz ACN, Brondani DJ, de Santana TI, et al. Biological evaluation of arylsemicarbazone derivatives as potential anticancer agents. Pharmaceuticals 2019;12:169. 10.3390/ph12040169 PMC6958387 31744203

[6] Zhai X, Bao G, Wang L, et al. Design, synthesis and biological evaluation of novel 4-phenoxy-6,7-disubstituted quinolines possessing (thio)semicarbazones as c-Met kinase inhibitors. Bioorg Med Chem 2016;24:1331–45. 26897090 10.1016/j.bmc.2016.02.003

[7] Ahsan MJ, Khalilullah H, Yasmin S, et al. Synthesis and anticonvulsant evaluation of 2-(substituted benzylidene/ethylidene)-N-(substituted phenyl)hydrazinecarboxamide analogues. Med Chem Res 2013;22:2746–54.

[8] Yogeeswari P, Sriram D, Thirumurugan R, et al. Discovery of N-(2,6-dimethylphenyl)-substituted semicarbazones as anticonvulsants: hybrid pharmacophore-based design. J Med Chem 2005;48:6202–11. 16190747 10.1021/jm050283b

[9] Yogeeswari P, Sriram D, Thirumurugan R, et al. Synthesis of N4-(2,4-dimethylphenyl) semicarbazones as 4-aminobutyrate aminotransferase inhibitors. Acta Pharm 2006;56:259–72. 19831276

[10] Ahsan MJ, Amir M, Bakht MA, et al. Synthesis and antimicrobial activity of *N*^1^-(3-Chloro-4-flourophenyl)-*N*^4^-substituted semicarbazone derivatives. Arabian J Chem 2016;9:S861–S866.

[11] Dimmock JR, Pandeya SN, Quail JW, et al. Evaluation of the semicarbazones, thoisemicarbazones and bis-carbohydrazones of some aryl alicyclic ketones from anticonvulsant and other biological properties. Eur J Med Chem 1995;30:303–14.

[12] Mishra V, Pandeya SN, Declercq E, et al. Syntheis of aryl semicarbazone of 4-aminoacetophenone and their anti-HIV activity. Pharmaceut Acta Helvet 1998;73:215–8. 10.1016/s0031-6865(98)00028-4 9861870

[13] Taroua M, Ribuot C, Pera MH, et al. New α, β and γ semicarbazone and thiosemicarbazone 1,3-ditholanes as radioprotectors. anticonvulsant activity. Eur J Med Chem 1996;31:589–95.

[14] Sriram D, Yogeeswari P, Thirumurugan R. Antituberculous activity of some aryl semicarbazone derivatives. Bioorg Med Chem Lett 2004;14:3923–4. 15225698 10.1016/j.bmcl.2004.05.060

[15] Cerecetto H, Maio RD, Gonzalez M, et al. Synthesis and antitrypanosomal evaluation of *E*-Isomers of 5-Nitro-2-Furaldehyde and 5-nitrothiophene-2-carboxaldehyde semicarbazone derivatives. structure-activity relationships. Eur J Med Chem 2000;35:343–50. 10785560 10.1016/s0223-5234(00)00131-8

[16] Cerecetto H, Maio RD, Ibarruri G, et al. Synthesis and anti-trypanosomal activity of novel 5-nitro-2-furaldehyde and 5-nitrothiophene-2-carboxaldehyde semicarbazone derivatives. Il Farmaco 1998;53:89–94. 9604315 10.1016/s0014-827x(97)00011-6

[17] Amir M, Ahsan MJ, Ali I. Synthesis of *N*^1^-(3-chloro-4-flourophenyl)-*N*^4^-substituted semicarbazones as novel anticonvulsant agents. Indian J Chem 2010;49B:1509–14.

[18] Alarcon-Rojo AD, Carrillo-Lopez L, Reyes-Villagrana MR, et al. Ultrasound and meat quality: a review. Ultrason Sonochem 2020;55: 369–82. 10.1016/j.ultsonch.2018.09.016 31027999

[19] Chemat F, Ashokkumar M. Preface: ultrasound in the processing of liquid foods, beverages and alcoholic drinks. Ultrason Sonochem 2017;38:753. 28237268 10.1016/j.ultsonch.2017.01.041

[20] F Chemat Zill-E-Huma MK. Applications of ultrasound in food technology: processing, preservation and extraction. Ultrason Sonochem 2011;18:813–35. 21216174 10.1016/j.ultsonch.2010.11.023

[21] Awad TS, Moharram HA, Shaltout OE, et al. Applications of ultrasound in analysis, processing and quality control of food: a review. Food Res Int 2012;48:410–27.

[22] Bakht MA, Geesi MH, Riadi Y, et al. Ultrasound-assisted extraction of some branded tea: optimization based on polyphenol content, antioxidant potential and thermodynamic study. Saudi J Biol Sci 2019;26:1043–52. 31303839 10.1016/j.sjbs.2018.07.013 PMC6601128

[23] Geesi MH, Moustapha ME, Bakht MA, Riadi Y. Ultrasound-accelerated green synthesis of new quinolin-2-thione derivatives and antimicrobial evaluation against *Escherichia coli* and *Staphylococcus aureus*. Sustainable Chem Pharm 2020;15:100195.

[24] Wang SY, Ji SJ, Su XM. A meldrum's acid catalyzed synthesis of bis(indolyl)methanes in water under ultrasonic condition. Chin J Chem 2008;26:22–4.

[25] Li JT, Li XL, Li TS. Synthesis of oximes under ultrasound irradiation. Ultrason Sonochem 2006;13:200–2. 16455283 10.1016/j.ultsonch.2005.11.011

[26] Zang H, Zhang Y, Zang Y, Cheng BW. An efficient ultrasound-promoted method for the one-pot synthesis of 7,10,11,12-tetrahydrobenzo[c]acridin-8(9H)-one derivatives. Ultrason Sonochem 2010;17:495–9. 20006532 10.1016/j.ultsonch.2009.11.003

[27] Jarag KJ, Pinjari DV, Pandit AB, Shankarling GS. Synthesis of chalcone (3-(4-fluorophenyl)-1-(4-methoxyphenyl)prop-2-en-1-one): advantage of sonochemical method over conventional method. Ultrason Sonochem 2011;18:617–23. 20980185 10.1016/j.ultsonch.2010.09.010

[28] Bakht MA, Ansari MJ, Riadi Y, et al. Benzalkonium chloride and urea based deep eutectic solvent (DES): a novel catalyst for the efficient synthesis of isoxazolines under ultrasonic irradiation. J Mol Liq 2016;224:1249–55.

[29] Mason TJ. Sonochemistry and the environment - providing a "green" link between chemistry, physics and engineering. Ultrason Sonochem 2007;14:476–83. 17207652 10.1016/j.ultsonch.2006.10.008

[30] Gao DM, Ma WL, Li TR, et al. An improved synthesis of 1,2-diarylethanols under conventional heating and ultrasound irradiation. Molecules 2012;17:10708–15. 22960865 10.3390/molecules170910708 PMC6268955

[31] Yadav JS, Reddy BVS, Reddy KS. Ultrasound-accelerated synthesis of chiral allylic alcohols promoted by indium metal. Tetrahedron 2003;59:5333–6.

[32] Liu T, Baek DR, Kim JS, et al. Green synthesis of silver nanoparticles with size distribution depending on reducing species in glycerol at ambient pH and temperatures. ACS Omega 2020;5:16246–54. 32656447 10.1021/acsomega.0c02066 PMC7346276

[33] Díaz-Álvarez AE, Francos J, Lastra-Barreira B, et al. Glycerol and derived solvents: new sustainable reaction media for organic synthesis. Chem Commun 2011;47:6208–27. 10.1039/c1cc10620a 21451852

[34] Quispe CAG, Coronado CJR, Carvalho JA Jr. Glycerol: production, consumption, prices, characterization and new trends in combustion. Renewable Sustainable Energy Rev 2013;27:475–93.

[35] WHO cancer reports. 2020; ISBN 978-92-4-000129-9.

[36] Mohamady S, Galal M, Eldehna WM, et al. Dual targeting of VEGFR2 and C-met kinases via the design and synthesis of substituted 3-(Triazolo-thiadiazin-3-yl)indolin-2-one derivatives as angiogenesis inhibitors. ACS Omega 2020;5:18872–86. 32775889 10.1021/acsomega.0c02038 PMC7408256

[37] Sogabe S, Kawakita Y, Igaki S, et al. Structure-based approach for the discovery of Pyrrolo[3,2-d]pyrimidine-based EGFR T790M/L858R mutant inhibitors. ACS Med Chem Lett 2013;4:201–5. 24900643 10.1021/ml300327z PMC4027575

[38] Ahsan MJ, Hassan MZ, Jadav SS, et al. Synthesis and biological potentials of 5-aryl-*N*-[4-(trifluoromethyl) phenyl]-1,3,4-oxadiazol-2-amines. Lett Org Chem 2020;17:133–40.

[39] Merla A, Goel S. Novel drugs targeting the epidermal growth factor receptor and its downstream pathways in the treatment of colorectal cancer: a systematic review. Chemother Res Pract 2012;2012:387172. 23097702 10.1155/2012/387172 PMC3477664

[40] Xu MJ, Johnson DE, Grandis JR. EGFR-targeted therapies in the post-genomic era. Cancer Metastasis Rev 2017;36:463–73. 28866730 10.1007/s10555-017-9687-8 PMC5693744

[41] Blair JA, Rauh D, Kung C, et al. Structure-guided development of affinity probes for tyrosine kinases using chemical genetics. Nat Chem Biol 2007;3:229–38. 17334377 10.1038/nchembio866

[42] Nath R, Pathania S, Grover G, Akhtar MJ. Isatin containing heterocycles for different biological activities: analysis of structure activity relationship. J Mol Str 2020;1222:128900.

[43] DTP Developmental therapeutic Programs: http://dtp.nci.nih.gov

[44] Monks A, Scudiero D, Skehan P, et al. Feasibility of a high-flux anticancer drug screen using a diverse panel of cultured human tumor cell lines. J Natl Cancer Inst 1991;83:757–66. 2041050 10.1093/jnci/83.11.757

[45] Boyd MR, Paull KD. Some practical considerations and applications of the National Cancer Institute *in vitro* anticancer drug discovery screen. Drug Dev Res 1995;34:91–109.

[46] Shoemaker RH. The NCI60 human tumour cell line anticancer drug screen. Nat Rev Cancer 2006;6:813–23. 16990858 10.1038/nrc1951

[47] EGFR Kinase domain T790M/L858R mutant: https://www.rcsb.org/structure/3W2R

[48] Canter D, Kutikov A, Golovine K, et al. Are all multi-targeted tyrosine kinase inhibitors created equal? An in vitro study of sunitinib and pazopanib in renal cell carcinoma cell lines. Can J Urol 2011;18:5819–25. 21854714 PMC3182114

[49] Toxicity prediction software. Available at: https://tox-new.charite.de/protox_II/index.php?site=home

[50] Banerjee P, Eckert AO, Schrey A, Preissner KR. ProTox-II: a webserver for the prediction of toxicity of chemicals. Nucleic Acids Res 2018;46:W257–W263. 29718510 10.1093/nar/gky318 PMC6031011

[51] Raies AB, Bajic VB. In silico toxicology: computational methods for the prediction of chemical toxicity. Wiley Interdiscip Rev Comput Mol Sci 2016;6:147–72. 27066112 10.1002/wcms.1240 PMC4785608

