# Supplementary material for: Ultrasound promoted green synthesis, anticancer evaluation, and molecular docking studies of hydrazines: a pilot trial
Source: J Enzyme Inhib Med Chem. 2021 Dec 11;37(1):135–44. doi: 10.1080/14756366.2021.1995727 (PMC8741255; doi:10.1080/14756366.2021.1995727)
Supplement: Supplemental Material [file IENZ_A_1995727_SM8756.pdf]

Ultrasound Promoted Green Synthesis, Anticancer Evaluation, and Molecular Docking Studies of Hydrazine Carboxamides: A Pilot Trial

Amena Ali[a], Abuzer Ali[b], Abu Tahir[c], Mohammed Afroz Bakht[d], Mohamed Jawed Ahsan[e,*]

[a] *Department of Pharmaceutical Chemistry, College of Pharmacy, Taif University, P.O. Box 11099, Taif 21944 Saudi Arabia*

[b] *Department of Pharmacognosy, College of Pharmacy, Taif University, P.O. Box 11099, Taif 21944 Saudi Arabia*

[c] *Department of Pharmacology, Raghukul College of Pharmacy, Bhopal, Madhya Pradesh 462 003, India*

[d] *Department of Chemistry, College of Science and Humanity Studies, Prince Sattam Bin Abdulaziz University, P.O. Box- 83, Al-Kharj 11942, Saudi Arabia*

[e] *Department of Pharmaceutical Chemistry, Maharishi Arvind College of Pharmacy, AmbabariCircle, Jaipur, Rajasthan 302 039, India*

***Corresponding authors**: Mohamed Jawed Ahsan (jawedpharma@gmail.com)

ELECTRONIC SUPPLEMENTARY INFORMATIONS

Experimental

General

Chemical were procured from Merk Germany, SD Fine. Melting point was recorded by an open tube method. The thin layer chromatography was run in chloroform-methanol solvent (9:1) and the spot were identified in iodine vapour. The NMR in DMSO-d_6 (^{1}H and ^{13}C) and mass spectra were recorded from Bruker AvIII HD-300 MHz FT NMR and Waters ACQUITY® TQD respectively. Ultrasonic Procesor model UP200Ht was used for the preparation of target compounds at a frequency of 20 KHz and 130 W.

*General method of synthesis of phenyl[substituted phenyl]carbamate (**3a-l**):*

A solution of substituted aniline (**1a-l**) (1 mmol) in triethylamine (1 mmol; ~1.4 mL) was added drop-wise to a solution of phenylchloroformate (**2**) (1 mmol; 3.25 mL) in chloroform (5 mL) and the reaction mixture was ultrasonicated for 20 min. After consumption of reactants the

precipitate was filtered, washed with water, dried and re-crystallized with ethanol to get phenyl[substituted phenyl]carbamate (**3a-l**). The conventional methods took 4 h to complete the reaction.[1,2]

*General method of synthesis of N-[substituted phenyl]hydrazinecarboxamide (**4a-l**)*

A solution of phenyl[substituted phenyl]carbamate (**3a-l**) (1 mmol) in 10 mL methylene chloride and hydrazine hydrate (2 mmol; ~0.12 mL) was ultrasonicated for 30-45 min. The precipitate of *N*-[substituted phenyl]hydrazinecarboxamide (**4a-l**) was obtained once the reaction completed. The conventional methods took 24 h to complete the reaction [1,2].

*General method of synthesis of N-(substituted phenyl)-2-(2-oxoindolin-3-ylidene)hydrazine-1-carboxamides (**6a-l**)*

The reaction mixture of 1*H*-Indole-2,3-dione (0.001 mol; 0.147 g) (**5**) and *N*-(substituted phenyl)hydrazinecarboxamide (**4a-l**) (0.001 mol) in water-glycerol (6:4) solvent (10 mL) was ultrasonicated at 130 W for 5-20 min. The crude product (precipitate) (**6a-l**) was collected by vacuum filtration once the reaction completed. The isolated crude product was re-crystallized with absolute ethanol. The conventional methods also reported elsewhere.[1,2]

*N-(4-Fluorophenyl)-2-(2-oxoindolin-3-ylidene)hydrazine-1-carboxamide (**6a**)*: IR 3372 and 3264 cm^{-1} NH, 1685 and 1680 cm^{-1} CONH, 1528 cm^{-1} N=C, 785 cm^{-1} CF; ^{1}H NMR (300 MHz DMSO- d_6): δ 6.91 (2H, d, *J* = 3.9 Hz, ArH), 7.03-7.20 (3H, m, ArH), 7.33-7.40 (1H, m, ArH), 7.57-7.65 (2H, m, ArH), 8.09 (1H, d, *J* = 7.8, ArH), 9.58 (1H, s, ArNH), 10.42 (1H, s, NH), 10.77 (1H, s, CONH); ^{13}C NMR (75 MHz DMSO- d_6): δ 164.7, 162.6, 152.4, 143.1, 138.3, 134.8, 131.9, 130.6, 125.2, 122.7-121.1 (m), 115.1-115.5 (m), 110.5; Anal. Calc. for $C_{15}H_{11}FN_4O_2$: C, 60.40; H, 3.72; N, 18.78 found: C, 60.28; H, 3.75; N, 18.70%; EI-MS m/z = 321.1 $(M+Na)^+$, 322.1 $(M+Na)^+$ Isotopomer.

*N-(4-Chlorophenyl)-2-(2-oxoindolin-3-ylidene)hydrazine-1-carboxamide (**6b**)*: IR 3365 and 3284 cm^{-1} NH, 1681 and 1676 cm^{-1} CONH, 1514 cm^{-1} N=C, 694 cm^{-1} CCl; ^{1}H NMR (300 MHz DMSO- d_6): δ 6.90-7.67 (7H, m, ArH), 8.09 (1H, d, *J* = 7.5 Hz, ArH) 9.69 (1H, s, ArNH), 10.45 (1H, s, NH), 10.78 (1H, s, CONH); ^{13}C NMR (75 MHz DMSO- d_6): δ 164.7, 162.2, 152.3, 151.2, 143.1, 141.7, 137.6, 135.1, 132.0, 128.7 (m), 126.6, 125.3, 120.8 (m), 115.5, 110.5; Anal. Calc.

for $C_{15}H_{11}ClN_4O_2$: C, 57.24; H, 3.52; N, 17.80 found: C, 57.18; H, 3.55; N, 17.72%; EI-MS m/z = 337.1 $(M+Na)^+$, 339.0 $(M+Na)^+$ Isotopomer.

*N-(4-Bromophenyl)-2-(2-oxoindolin-3-ylidene)hydrazine-1-carboxamide (**6c**)*: IR 3365 and 3286 cm^{-1} NH, 1681 and 1676 cm^{-1} CONH, 1519 cm^{-1} N=C, 675 cm^{-1} CBr; ^1H NMR (400 MHz DMSO- d_6): δ 6.93 (1H, d, J = 8 Hz, ArH), 7.06-7.10 (1H, m, ArH), 7.36-7.41 (3H, m, ArH), 7.63-7.67 (2H, m, ArH), 7.65 (2H, d, J = 7.9 Hz, ArH), 7.77 (1H, d, J = 7.0 Hz, ArH), 8.11 (1H, d, J = 7.2), 9.87 (1H, s, ArNH), 10.54 (1H, s, NH), 10.86 (1H, s, CONH); ^{13}C NMR (75 MHz DMSO- d_6): δ 164.7, 152.3, 143.1, 137.4, 135.1, 132.0, 128.7, 126.6, 125.3, 121.6, 120.8, 115.5, 110.5; Anal. Calc. for $C_{15}H_{11}BrN_4O_2$: C, 50.16; H, 3.09; N, 15.60 found: C, 50.10; H, 3.05; N, 15.65%; EI-MS m/z = 381.1 $(M+Na)^+$.

*N-(4-Trifluoromethylphenyl)-2-(2-oxoindolin-3-ylidene)hydrazine-1-carboxamide (**6d**)*: IR 3365 and 3218 cm^{-1} NH, 1684 and 1678 cm^{-1} CONH, 1515 cm^{-1} N=C, 786 cm^{-1} CF; ^1H NMR (300 MHz DMSO- d_6): δ 7.39-7.65 (6H, m, ArH), 7.77 (1H, m, ArH), 8.10 (1H, m, ArH), 9.61 (1H, s, ArNH), 10.42 (1H, s, NH), 10.78 (1H, s, CONH); ^{13}C NMR (75 MHz DMSO- d_6): δ 168.7, 152.6, 142.9, 141.2, 134.6, 132.3, 131.4, 129.7, 125.3, 124.7, 124.3, 121.9, 119.1, 117.7; Anal. Calc. for $C_{16}H_{11}F_3N_4O_2$: C, 55.18; H, 3.18; N, 16.09 found: C, 55.11; H, 3.15; N, 16.04%; EI-MS m/z = 371.2 $(M+Na)^+$.

*N-(4-Methylphenyl)-2-(2-oxoindolin-3-ylidene)hydrazine-1-carboxamide (**6e**)*: IR 3365 and 3292 cm^{-1} NH, 1680 and 1672 cm^{-1} CONH, 1518 cm^{-1} N=C; ^1H NMR (400 MHz DMSO- d_6): δ 2.49 (3H, s, CH_3), 6.93 (1H, d, J = 4 Hz, ArH), 7.0-7.10 (1H, m, ArH), 7.37-7.42 (3H, m, ArH), 7.64-7.67 (2H, d, J = 12 Hz, ArH), 8.09 (2H, d, J = 8.0), 9.86 (1H, s, ArNH), 10.51 (1H, s, NH), 10.87 (1H, s, CONH); ^{13}C NMR (75 MHz DMSO- d_6): δ 164.8, 152.1, 143.0, 135.9, 134.5, 131.9, 129.2, 125.1, 121.6, 119.2, 115.6, 110.5, 20.4; Anal. Calc. for $C_{16}H_{14}N_4O_2$: C, 65.30; H, 4.79; N, 19.04 found: C, 65.21; H, 4.75; N, 19.01%; EI-MS m/z = 317 $(M+Na)^+$, 333 $(M+K)^+$

*N-(4-Methoxyphenyl)-2-(2-oxoindolin-3-ylidene)hydrazine-1-carboxamide (**6f**)*: IR 3365 and 3290 cm^{-1} NH, 1685 and 1678 cm^{-1} CONH, 1516 cm^{-1} N=C; ^1H NMR (300 MHz DMSO- d_6): δ 3.88 (3H, s, OCH_3), 6.91 (1H, d, J = 7.5 Hz, ArH), 7.03-7.12 (2H, m, ArH), 7.18-7.25 (2H, m, ArH), 7.35-7.40 (1H, m, ArH), 7.75 (1H, d, J = 8.1), 8.13 (1H, d, J = 7.5), 9.23 (1H, s, ArNH), 10.66 (1H, s, NH), 10.76 (1H, s, CONH); ^{13}C NMR (75 MHz DMSO- d_6): δ 168.7, 158.9, 152.66, 141.6, 134.6, 131.9, 131.1, 129.5, 124.5, 119.9, 119.3, 117.7, 114.7, 56.2; Anal. Calc. for

$C_{16}H_{14}N_4O_3$: C, 61.93; H, 4.55; N, 18.06 found: C, 61.85; H, 4.51; N, 18.01%; EI-MS m/z = 333 $(M+Na)^+$, 349 $(M+K)^+$

*N-(2-Chlorophenyl)-2-(2-oxoindolin-3-ylidene)hydrazine-1-carboxamide (**6g**)*: IR 3365 and 3288 cm^{-1} NH, 1680 and 1677 cm^{-1} CONH, 1517 cm^{-1} N=C, 696 cm^{-1} CCl; ^{1}H NMR (400 MHz DMSO- d_6): δ 6.89-6.93 (1H, m, ArH), 7.04-7.19 (2H, m, ArH), 7.33-7.40 (2H, m, ArH), 7.50-7.57 (1H, m, ArH), 8.16 (1H, d, *J* = 8 Hz, ArH), 9.31 (1H, s, ArNH), 10.78 (1H, s, NH), 10.90 (1H, s, CONH); ^{13}C NMR (75 MHz DMSO- d_6): δ 164.4, 152.2, 143.4, 135.1, 134.7, 132.2, 130.9, 129.3, 127.8, 125.6, 125.2, 123.1, 121.8, 121.6, 115.3; Anal. Calc. for $C_{15}H_{11}ClN_4O_2$: C, 57.24; H, 3.52; N, 17.80 found: C, 57.19; H, 3.56; N, 17.75%; EI-MS m/z = 337.0 $(M+Na)^+$, 339.0 $(M+Na)^+$ Isotopomer.

*N-(2-Methylphenyl)-2-(2-oxoindolin-3-ylidene)hydrazine-1-carboxamide (**6h**)*: IR 3365 and 3285 cm^{-1} NH, 1680 and 1674 cm^{-1} CONH, 1512 cm^{-1} N=C; ^{1}H NMR (400 MHz DMSO- d_6): δ 6.91-6.93 (1H, m, ArH), 7.05-7.10 (2H, m, ArH), 7.19-7.25 (2H, m, ArH), 7.36-7.40 (1H, m, ArH), 7.71 (1H, d, *J* = 8 Hz, ArH), 8.13 (1H, d, *J* = 8 Hz, ArH), 9.31 (1H, s, ArNH), 10.78 (1H, s, NH), 10.90 (1H, s, CONH); ^{13}C NMR (75 MHz DMSO- d_6): δ 164.6, 152.5, 143.2, 136.1, 134.4, 132.0, 130.3, 129.5, 126.3, 125.3, 124.2, 122.4, 121.6, 115.5, 110.5, 17.7; Anal. Calc. for $C_{16}H_{14}N_4O_2$: C, 65.30; H, 4.79; N, 19.04 found: C, 65.23; H, 4.74; N, 19.00%; EI-MS m/z = 317 $(M+Na)^+$, 333 $(M+K)^+$.

*N-(2-Methoxyphenyl)-2-(2-oxoindolin-3-ylidene)hydrazine-1-carboxamide (**6i**)*: IR 3365 and 3298 cm^{-1} NH, 1686 and 1680 cm^{-1} CONH, 1518 cm^{-1} N=C; ^{1}H NMR (300 MHz DMSO- d_6): δ 3.89 (3H, s, OCH_3), 6.95-7.12 (5H, m, ArH), 7.32-7.40 (1H, m, ArH), 8.14-8.18 (2H, m, ArH), 9.22 (1H, s, ArNH), 10.76 (2H, s, CONH); ^{13}C NMR (75 MHz DMSO- d_6): δ 164.5, 152.0, 148.2, 143.2, 134.4, 132.0, 127.2, 126.8, 123.7, 121.5, 120.6, 118.9, 115.4, 111.0, 110.9, 55.8; Anal. Calc. for $C_{16}H_{14}N_4O_3$: C, 61.93; H, 4.55; N, 18.06 found: C, 61.88; H, 4.51; N, 18.02%; EI-MS m/z = 333.1 $(M+Na)^+$.

*N-(2,4-Dimethylphenyl)-2-(2-oxoindolin-3-ylidene)hydrazine-1-carboxamide (**6j**)*: IR 3365 and 3294 cm^{-1} NH, 1680 and 1674 cm^{-1} CONH, 1513 cm^{-1} N=C; ^{1}H NMR (300 MHz DMSO- d_6): δ 2.23 (3H, s, CH_3), 2.25 (3H, s, CH_3), 6.90-7.10 (4H, m, ArH), 7.32-7.39 (1H, s, ArH), 7.57 (1H, d, *J* = 11.6 Hz, ArH), 8.12 (1H, d, *J* = 10.4), 8.81 (1H, s, ArNH), 10.59 (1H, s, NH), 10.74 (1H, s, CONH); ^{13}C NMR (75 MHz DMSO- d_6): δ 164.6, 152.6, 143.1, 141.6, 134.2, 133.4, 131.9,

130.8, 129.7, 126.7, 125.3, 122.8, 121.5, 115.5, 110.8, 20.4, 17.6; Anal. Calc. for $C_{17}H_{16}N_4O_2$: C, 66.22; H, 5.23; N, 18.17 found: C, 66.17; H, 5.19; N, 18.14%; EI-MS m/z = 331.1 $(M+Na)^+$.

N-(2,6-Dimethylphenyl)-2-(2-oxoindolin-3-ylidene)hydrazine-1-carboxamide (***6k***): Mp 190 ºC found, 190 ºC (reported)[2]; ^{1}H NMR (400 MHz DMSO- d_6): δ 2.21 (6H, s, CH_3), 6.92 (1H, d, *J* = 8 Hz, ArH), 7.05-7.10 (4H, m, ArH), 8.13 (1H, d, *J* = 8, ArH), 8.95 (1H, s, ArNH), 10.53 (1H, s, NH), 10.81 (1H, s, CONH); ^{13}C NMR (75 MHz DMSO- d_6): δ 164.8, 153.0, 143.0, 135.6, 134.5, 131.8, 127.8, 126.5, 125.2, 121.6, 115.6, 110.4, 18.1; EI-MS m/z = 331 $(M+Na)^+$, 347 $(M+K)^+$.

N-(3-Chloro-4-fluorophenyl)-2-(2-oxoindolin-3-ylidene)hydrazine-1-carboxamide (***6l***): IR 3365 and 3290 cm^{-1} NH, 1681 and 1675 cm^{-1} CONH, 1518 cm^{-1} N=C, 784 cm^{-1} CF, 694 cm^{-1} CCl; ^{1}H NMR (300 MHz DMSO- d_6): δ 6.92 (1H, m, ArH), 7.09 (1H, m, ArH), 7.34-7.37 (2H, m, ArH), 7.50-7.59 (1H, m, ArH), 7.89-7.92 (1H, m, ArH), 8.09 (1H, d, *J* = 10 Hz, ArH), 9.73 (1H, s, ArNH), 10.50 (1H, s, NH), 10.78 (1H, s, CONH); ^{13}C NMR (75 MHz DMSO- d_6): δ 164.7, 155.8, 152.6, 143.2, 138.5, 135.9, 135.3, 132.1, 125.4, 121.6, 120.8, 119.8, 117.8, 115.5, 110.9; Anal. Calc. for $C_{15}H_{10}ClFN_4O_2$: C, 54.15; H, 3.04; N, 16.84 found: C, 54.11; H, 3.06; N, 16.80%; EI-MS m/z = 355.1 $(M+Na)^+$, 357.0 $(M+Na)^+$ Isotopomer.

References

1. M.J. Ahsan, M.Z. Hassan, S.S. Jadav, M.H. Geesi, M.A. Bakht, Y. Riadi, Salahuddin, M.S. Akhtar, M.S. Mallick, M.H. Akhtar, Synthesis and Biological Potentials of 5-aryl-*N*-[4-(trifluoromethyl) phenyl]-1,3,4-oxadiazol-2-amines. Lett. Org. Chem. 17 (2020) 133-140**.** http://dx.doi.org/10.2174/1570178616666190401193928

2. P. Yogeeswari, D. Sriram, R. Thirumurugan, A. Saxena, J. Stables, J. Vaigunda, Raghuvendran, K. Suddan, R.K. Pavana, Discovery of N-(2,6-Dimethyphenyl)-substituted semicarbazones as Anticonvulsants: Hybrid Pharmacophore-Based Design, J. Med. Chem. 48 (2005) 6202-6211. https://doi.org/10.1021/jm050283b

Table 1S. The growth percent (GP) of hydrazine carboxamides (**6a-l**) on 60 NCI cancer cell lines at 10 µM.

**Panels**	**Cell lines**	**6a**	**6b**	**6c**	**6d**	**6e**	**6f**	**6g**	**6h**	**6i**	**6j**	**6k**	**6l**
Leukemia	CCRF-CEM	95.36	-8.91	-43.84	99.59	-	105.01	91.94	101.74	89.88	92.70	74.89	102.15
	HL-60(TB)	94.49	38.90	70.19	70.18	111.03	102.46	78.35	93.52	93.78	35.27	99.20	96.76
	K-562	85.00	81.46	79.05	89.67	100.35	96.70	88.06	95.96	91.19	94.33	98.25	92.05
	MOLT-4	92.48	87.25	90.21	93.03	96.49	103.13	93.34	98.54	93.44	94.47	98.20	98.77
	RPMI-8226	91.04	56.12	66.91	96.58	87.43	100.50	84.80	98.21	84.80	85.38	80.36	91.84
	SR	84.24	79.08	76.66	83.49	92.52	92.97	77.08	96.36	83.89	96.37	99.36	94.90
Non-Small Lung Cancer	A549/ATCC	87.52	92.55	98.91	102.88	99.34	98.91	107.15	102.17	95.70	93.38	91.64	99.40
	EKVX	94.24	86.54	86.58	87.15	84.41	95.07	86.64	93.85	83.93	89.60	93.56	81.23
	HOP-62	83.18	98.64	98.37	95.45	98.10	90.96	104.61	100.80	98.77	99.15	96.49	89.20
	HOP-92	94.01	80.03	66.54	58.23	84.76	85.70	90.30	89.16	80.29	77.45	74.84	63.38
	NCI-H226	91.74	95.52	83.57	87.79	84.23	90.49	87.12	97.52	86.47	88.51	83.16	88.47
	NCI-H23	96.84	93.00	100.72	83.80	89.40	91.65	80.10	92.66	89.81	88.25	83.24	89.16
	NCI-H322M	93.77	75.30	70.90	89.66	82.80	85.86	73.51	101.81	81.18	77.15	79.57	93.10
	NCI-H460	108.44	91.30	92.12	97.34	103.07	94.23	92.07	103.47	104.02	100.43	106.50	102.05
	NCI-H522	78.51	89.82	72.25	84.04	85.27	88.74	78.59	84.35	83.02	79.94	85.31	88.94
Colon Cancer	Colo205	102.90	99.54	97.83	93.92	96.45	97.79	104.53	105.95	85.21	103.70	109.41	91.34
	HCC-2998	100.19	99.04	104.12	105.43	105.05	90.85	78.16	103.79	94.37	83.73	85.15	83.22
	HCT-116	81.95	94.42	88.60	92.83	86.53	85.17	89.84	96.90	93.02	88.76	93.86	93.15
	HCT-15	96.96	87.27	76.98	93.29	85.94	103.89	68.05	94.73	94.36	92.03	101.57	58.22
	HT29	101.67	93.54	97.60	101.95	108.78	103.02	101.08	100.48	105.35	107.10	105.59	108.65
	KM12	85.55	81.12	92.36	88.46	93.29	98.09	53.55	98.60	94.83	95.82	96.54	54.34

	SW-620	89.37	90.28	91.56	97.43	84.62	86.41	85.21	106.80	84.36	90.62	86.33	101.69
CNS Cancer	SF-268	103.85	100.65	86.21	96.98	96.87	97.08	96.78	99.84	99.10	97.09	94.56	97.80
	SF-295	95.01	101.31	93.00	99.34	96.89	93.96	94.05	99.55	94.13	103.27	97.52	97.57
	SF-539	94.93	83.84	83.57	97.58	89.61	90.60	95.72	96.54	98.33	98.60	84.92	99.25
	SNB-19	97.00	89.25	96.54	100.83	88.47	85.38	91.06	99.25	89.69	93.78	94.99	96.55
	SNB-75	91.31	101.45	104.54	92.45	95.10	88.94	82.02	114.11	82.66	93.77	71.88	83.21
	U251	90.70	100.41	100.90	96.06	98.33	95.66	92.23	99.42	97.27	95.95	102.61	101.21
Melanoma	LOX IMVI	92.20	86.52	78.01	93.62	94.27	97.18	96.54	97.94	98.25	91.86	95.80	95.01
	MALME-3M	97.56	96.29	86.12	94.59	90.10	85.71	96.90	98.10	95.81	89.71	87.94	101.06
	M14	99.74	95.30	89.75	95.62	93.14	93.88	94.71	102.29	95.09	95.00	87.05	94.85
	MDA-MB-435	98.25	95.48	90.43	106.38	105.09	106.76	84.50	111.07	91.01	96.34	93.67	100.95
	SK-MEL-2	102.40	99.74	93.52	103.12	105.43	96.56	101.96	103.69	99.98	105.61	111.59	103.04
	SK-MEL-28	107.83	91.47	89.33	113.54	94.72	91.14	104.65	114.60	103.76	87.26	94.02	110.53
	SK-MEL-5	100.51	90.72	81.33	102.68	100.81	98.45	87.56	108.09	94.08	93.76	95.70	91.99
	UACC-257	93.95	95.79	114.71	102.88	97.20	95.32	92.01	105.86	89.03	98.46	93.58	108.51
	UACC-62	79.31	75.72	79.34	89.94	86.83	79.52	78.77	90.87	79.17	80.65	79.06	83.30
Ovarian Cancer	IGROV1	85.20	80.23	85.24	82.59	79.02	81.20	65.28	105.66	81.96	82.79	79.34	72.20
	OVCAR-3	101.94	97.51	97.40	95.93	95.01	98.76	100.55	106.13	106.62	90.24	102.71	89.57
	OVCAR-4	103.43	98.15	102.43	96.37	101.00	102.66	71.05	100.58	104.63	108.71	100.01	76.60
	OVCAR-5	90.35	85.99	81.38	106.43	95.44	92.59	93.65	108.22	86.67	89.08	89.43	96.98
	OVCAR-8	96.74	101.53	98.86	101.14	98.03	96.74	97.50	102.60	92.91	93.69	92.36	96.69
	NCI/ADR-RES	103.58	110.49	97.17	96.76	107.73	102.25	93.02	100.74	94.06	96.22	92.24	91.66
	SK-OV-3	80.54	96.53	96.97	86.51	95.72	94.08	94.43	94.83	97.81	89.99	96.78	85.71

Renal cancer	786-0	97.29	97.82	89.61	102.47	99.87	102.18	96.85	99.50	90.42	92.08	101.22	98.60
	A498	102.44	77.61	72.00	105.25	96.44	107.93	96.48	108.04	90.73	78.08	85.49	104.71
	ACHN	92.79	80.52	83.70	97.97	88.28	87.71	98.96	100.91	92.98	81.97	89.44	94.56
	CAKI-1	81.54	89.16	76.25	81.39	90.31	89.05	80.47	88.19	73.32	85.86	80.49	81.93
	RXF 393	109.68	96.28	82.41	100.36	90.92	95.34	100.93	97.51	100.50	96.54	100.18	92.08
	SN12C	94.59	97.07	99.33	97.89	100.34	89.20	86.40	95.65	93.45	94.66	89.89	95.85
	TK-10	112.67	100.61	90.04	128.26	101.21	103.43	107.03	122.10	117.61	108.56	105.95	116.62
	UO-31	72.77	69.25	66.79	68.26	65.05	71.45	68.05	81.67	58.68	64.50	65.00	65.78
Prostate Cancer	PC-3	85.86	89.76	91.92	83.26	88.95	93.83	92.46	91.21	89.74	84.79	90.80	86.02
	DU-145	104.78	107.59	93.29	97.98	102.60	105.73	98.84	109.11	102.07	103.62	108.12	103.27
Breast cancer	MCF7	71.29	76.30	79.08	67.75	84.31	86.23	19.83	75.06	58.98	87.23	88.70	24.08
	MDA-MB-231/ATCC	94.04	102.09	109.73	88.06	87.84	91.08	85.14	94.31	82.74	89.67	96.06	89.89
	HS 578T	96.15	95.08	87.42	99.99	91.37	89.34	89.43	97.53	87.83	91.49	85.44	98.35
	BT-549	89.65	102.97	88.96	98.69	92.29	93.60	96.64	98.06	97.92	97.09	96.58	101.95
	T-47D	66.16	87.16	91.55	58.44	92.65	92.41	42.01	94.14	68.61	96.43	92.61	47.01
	MDA-MB-468	103.45	81.65	70.45	87.38	82.76	96.00	11.46	92.21	86.14	91.54	106.63	33.99
**Mean**	**-**	**93.52**	**88.45**	**85.97**	**93.46**	**93.56**	**94.11**	**86.16**	**99.39**	**90.93**	**91.17**	**92.39**	**89.53**

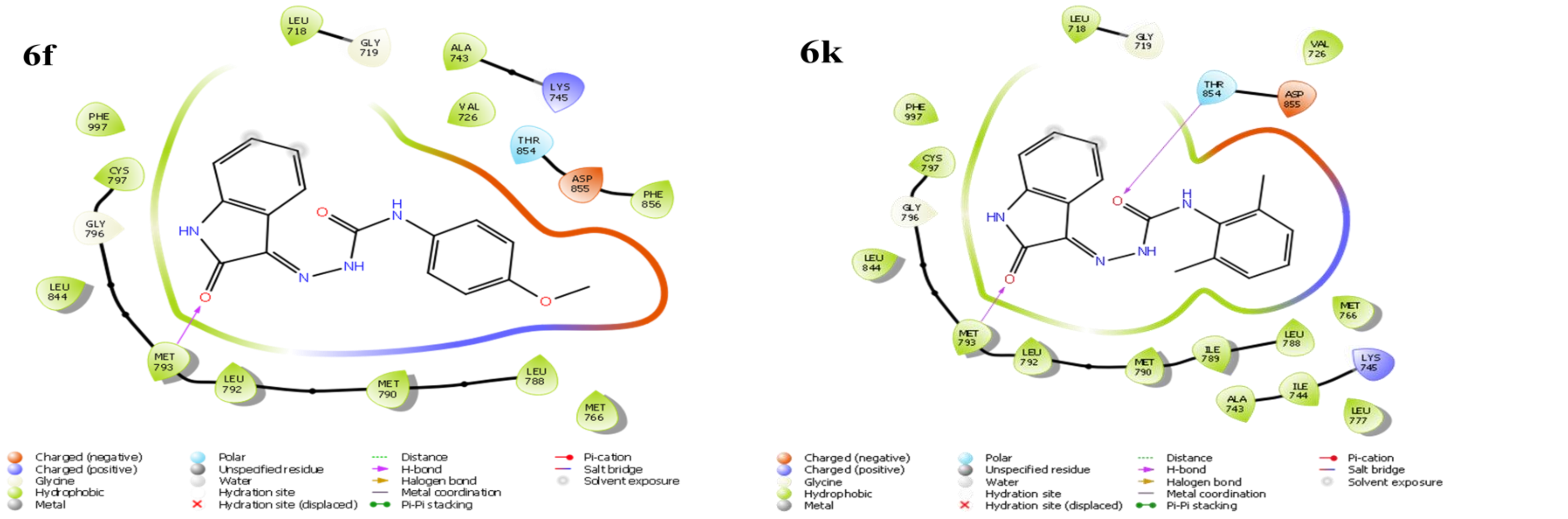

Fig 1S. The 2D interaction of ligands **6f** and **6k** with the binding site of EGFR

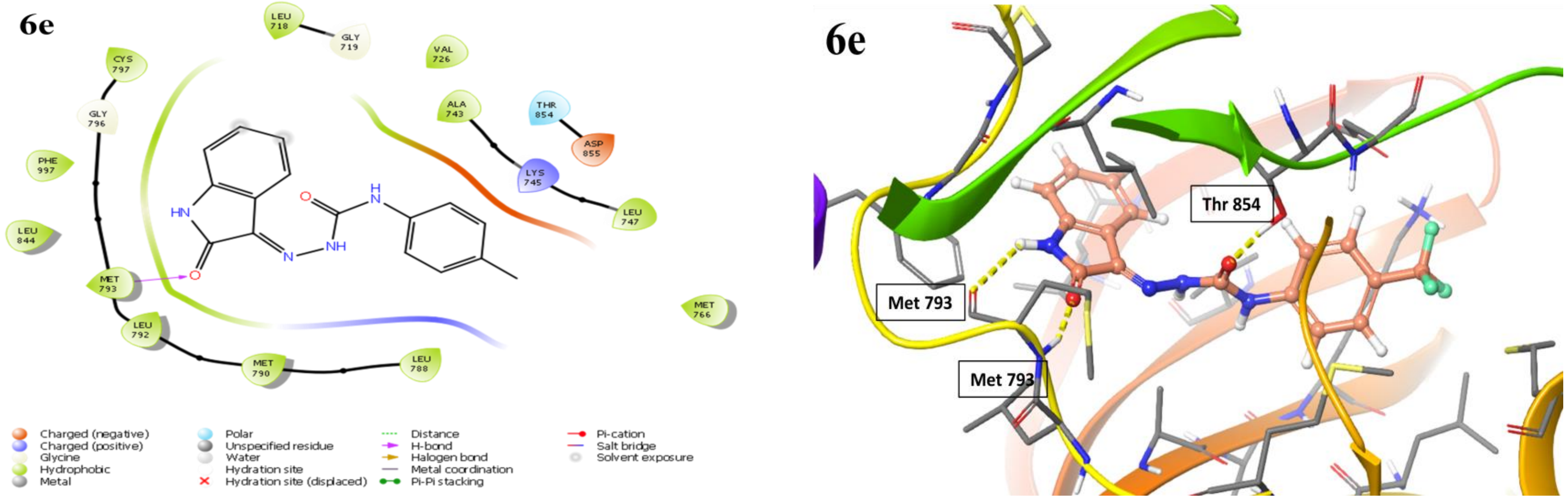

Fig 2S. The 2D and 3D interaction of ligand **6e** with the binding site of EGFR

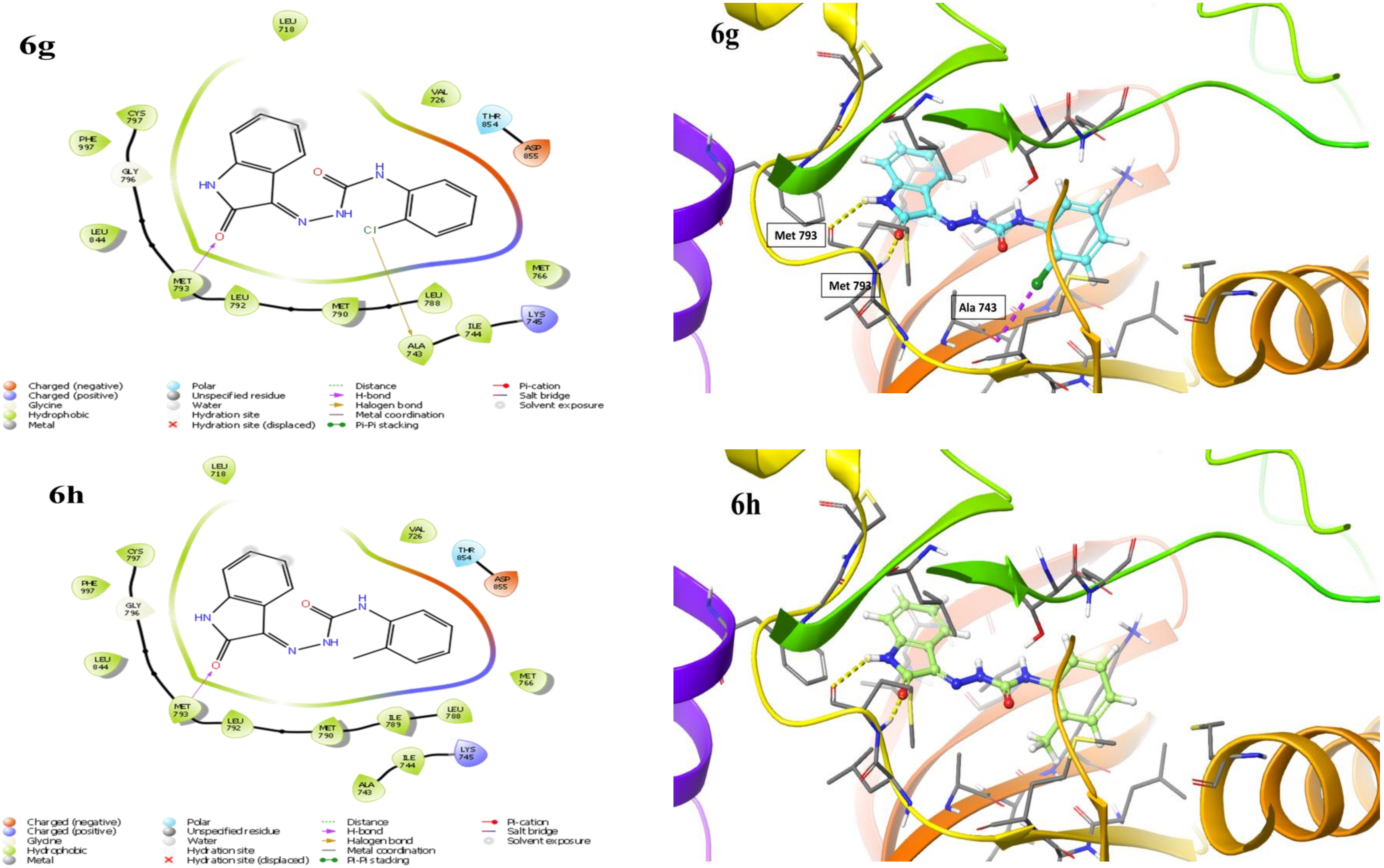

Fig 3S. The 2D and 3D interactions of ligands **6g**, and **6h** with binding site of EGFR

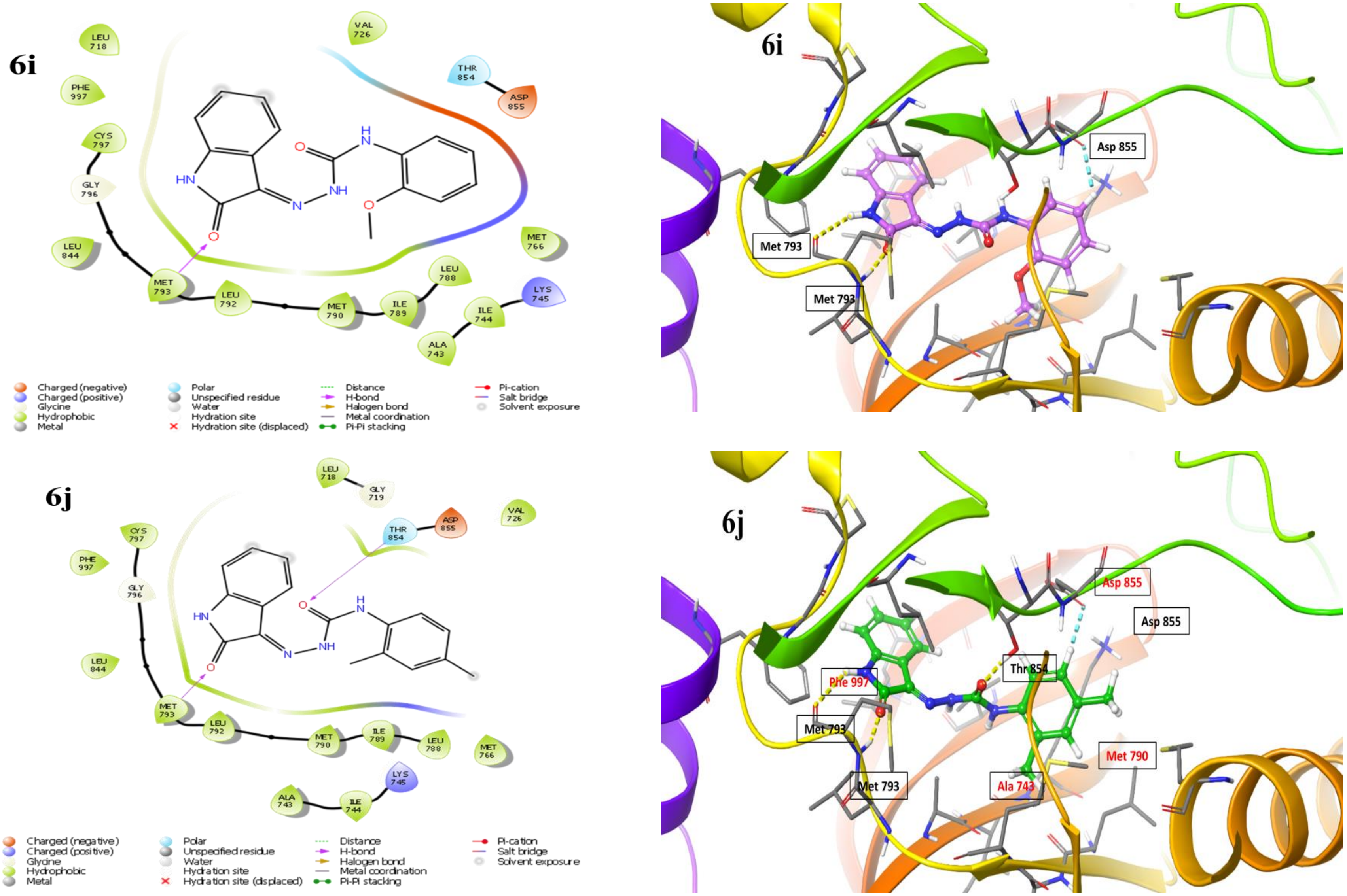

Fig 4S. The 2D and 3D interactions of ligands **6j**, and **6k** with active site of EGFR

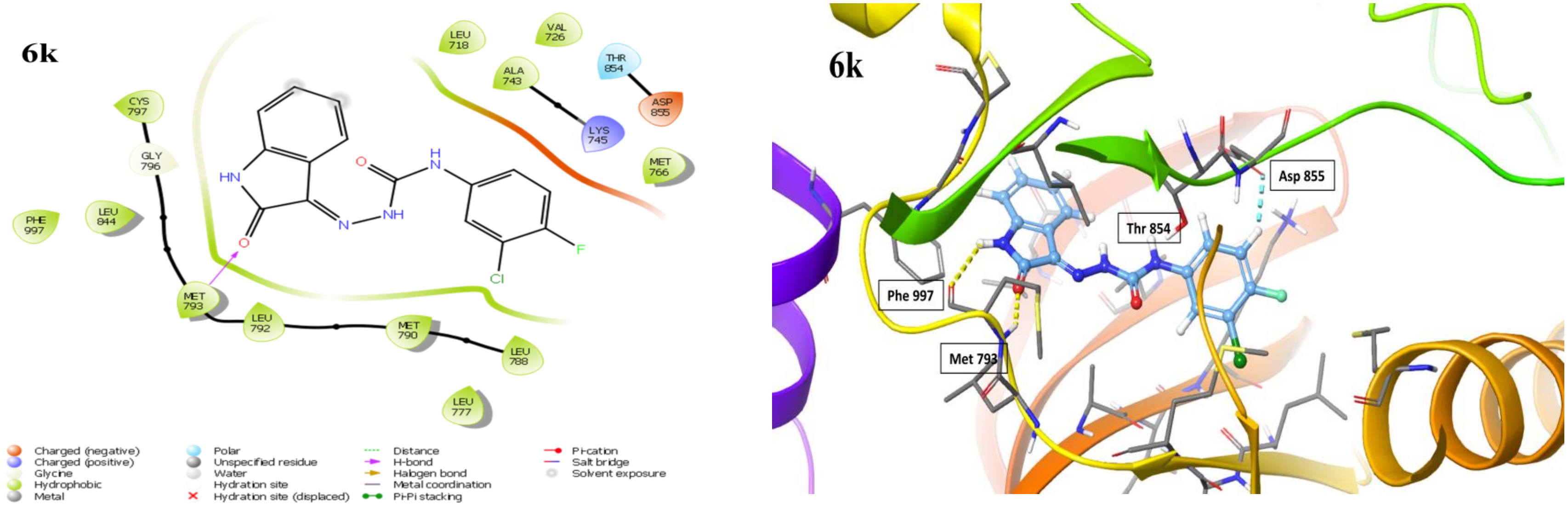

Fig 5S. The 2D and 3D interactions of ligand **6k** with the active site of EGFR

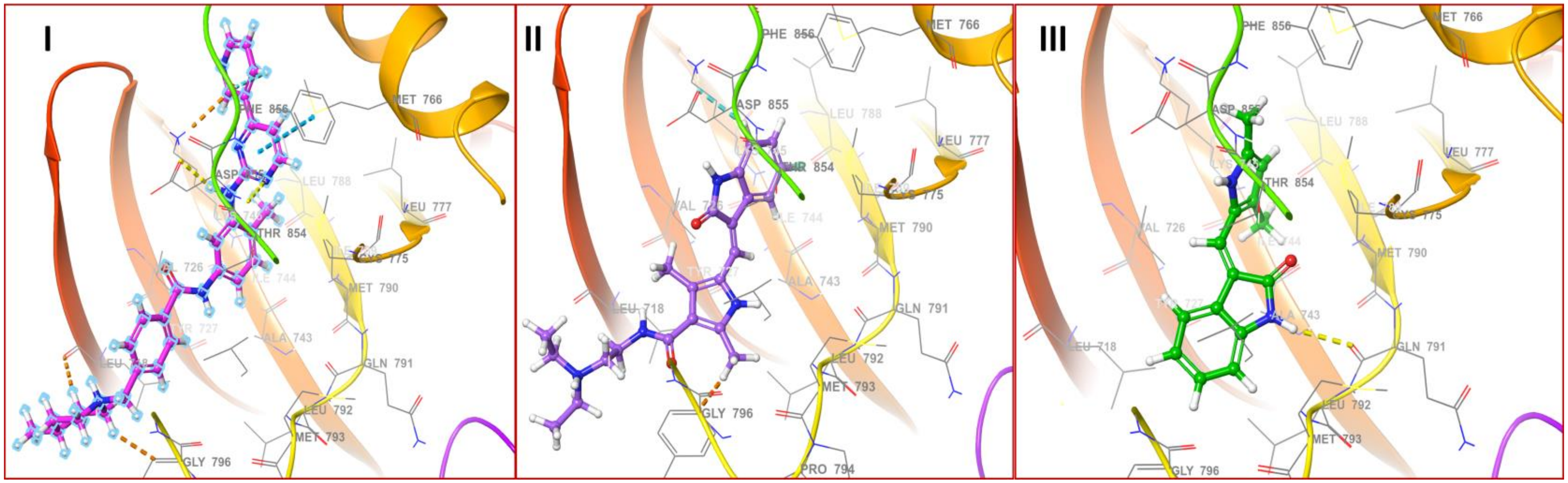

Fig 6S. The 3D interactions of ligands Imatinib (**I**), Sunitinib (**II**) and Semaxanib (**III**) against the active site of EGFR

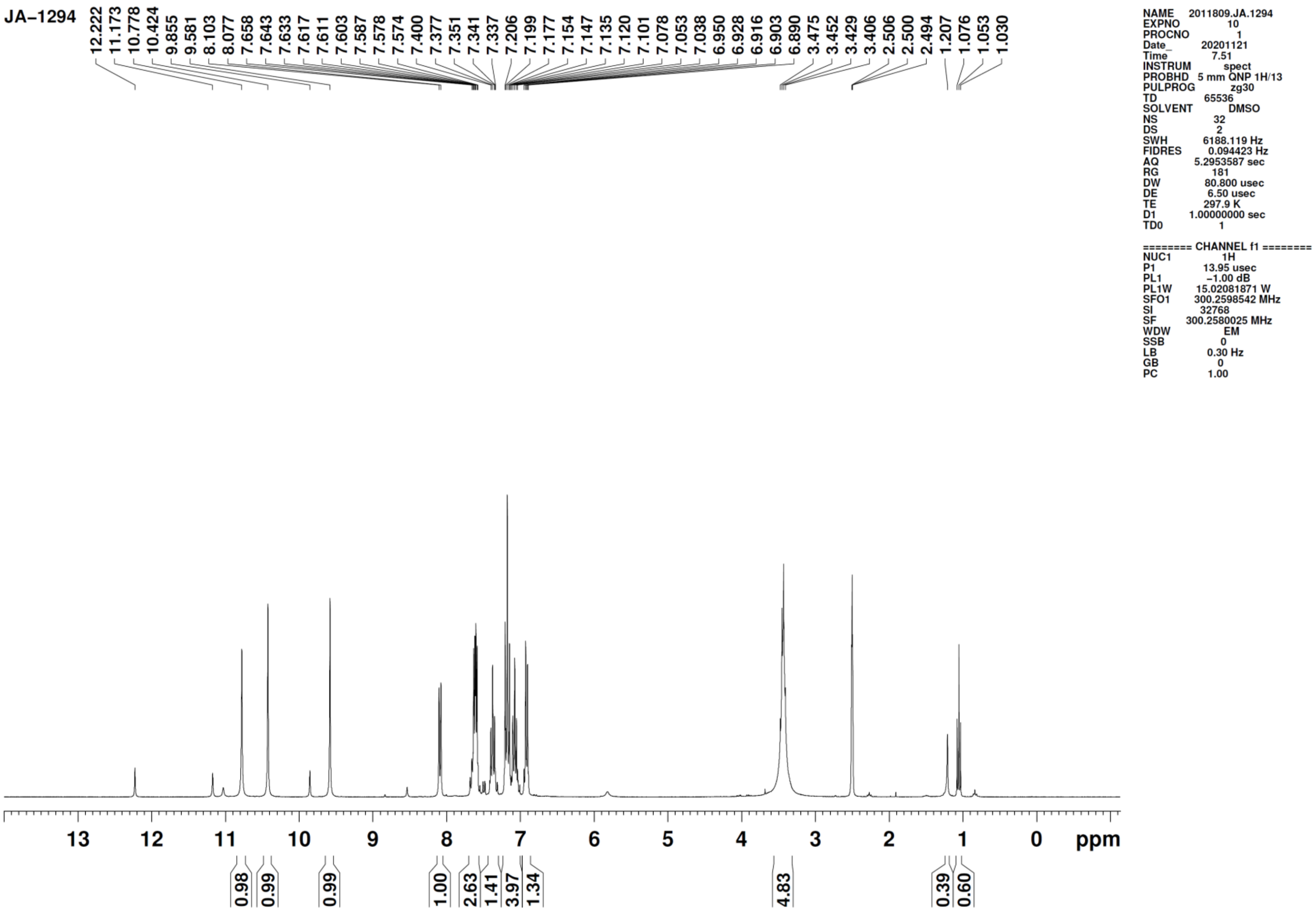

^{1}H NMR spectra of Compound **6a**

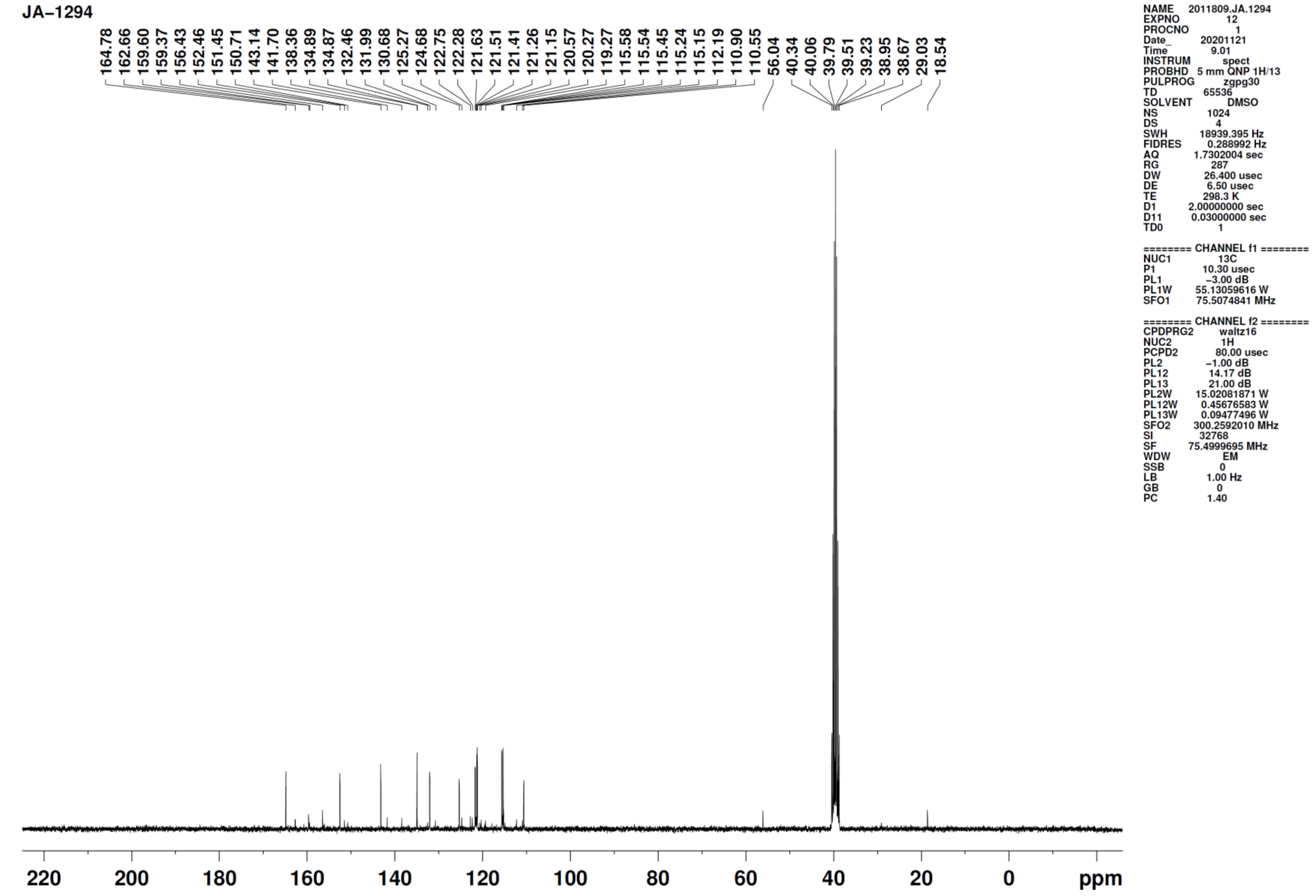

^{13}C NMR spectra of Compound **6a**

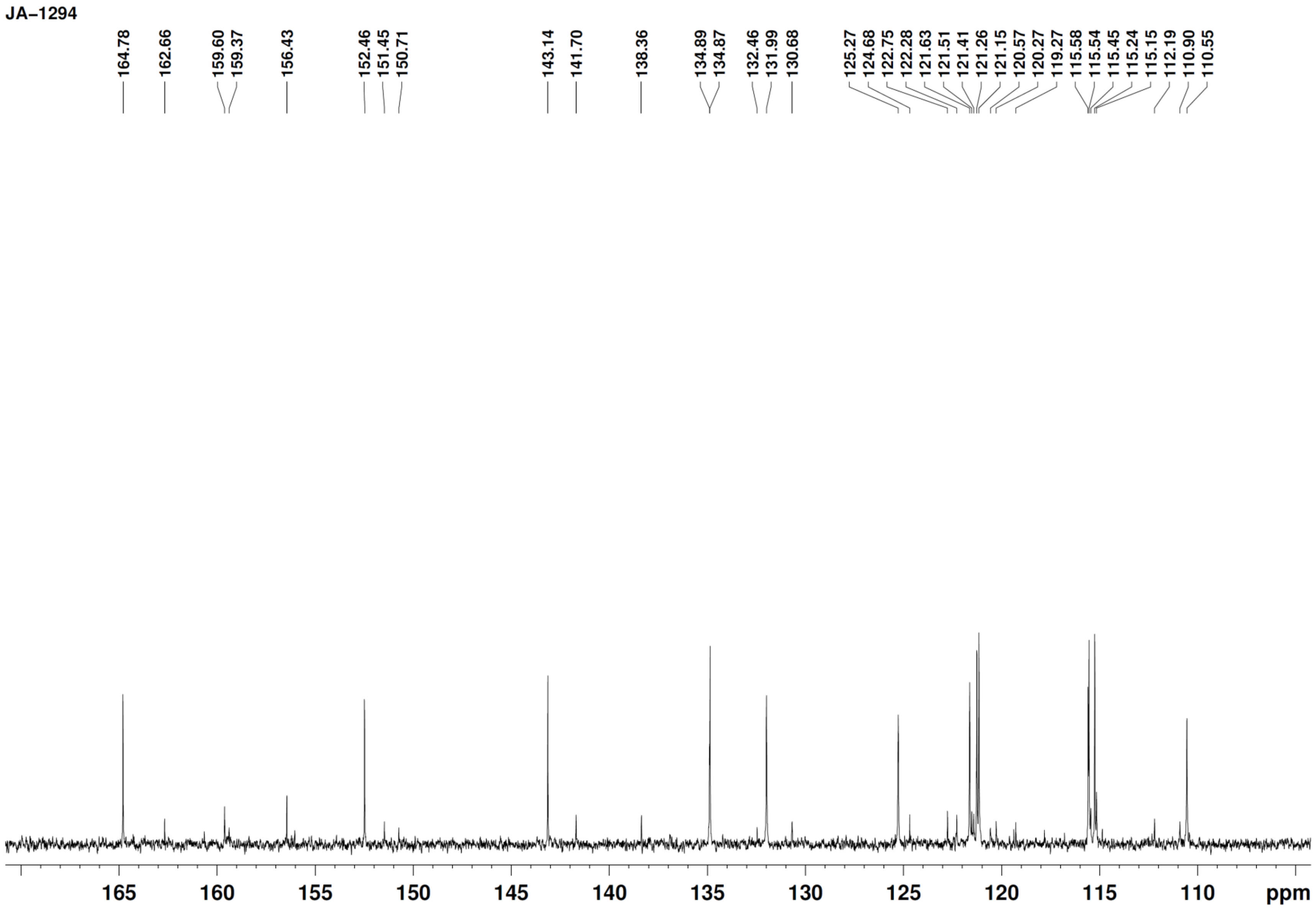

^{13}C NMR spectra of Compound **6a** showing multiplicity in the peaks

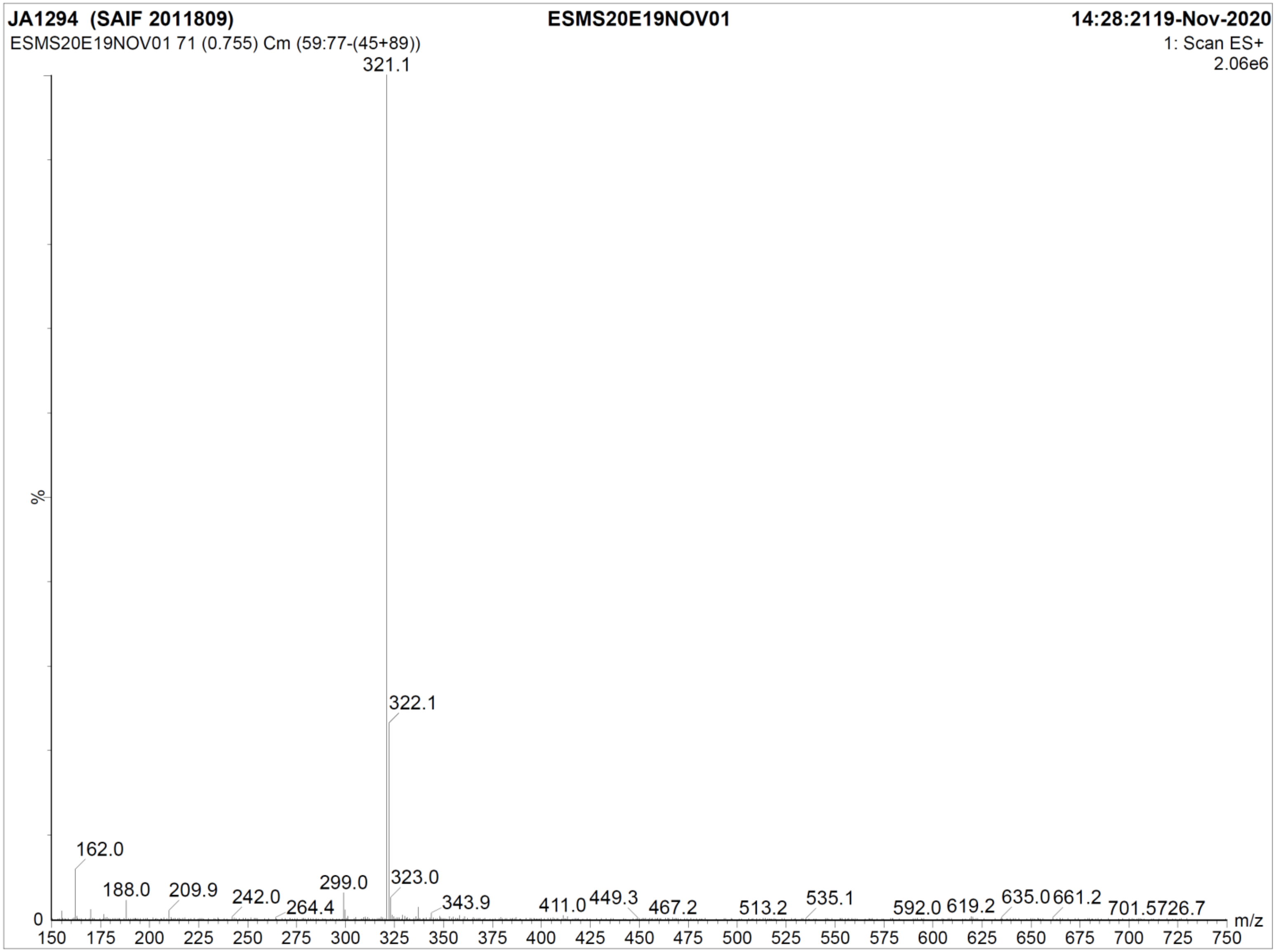

Mass spectra of Compound **6a**

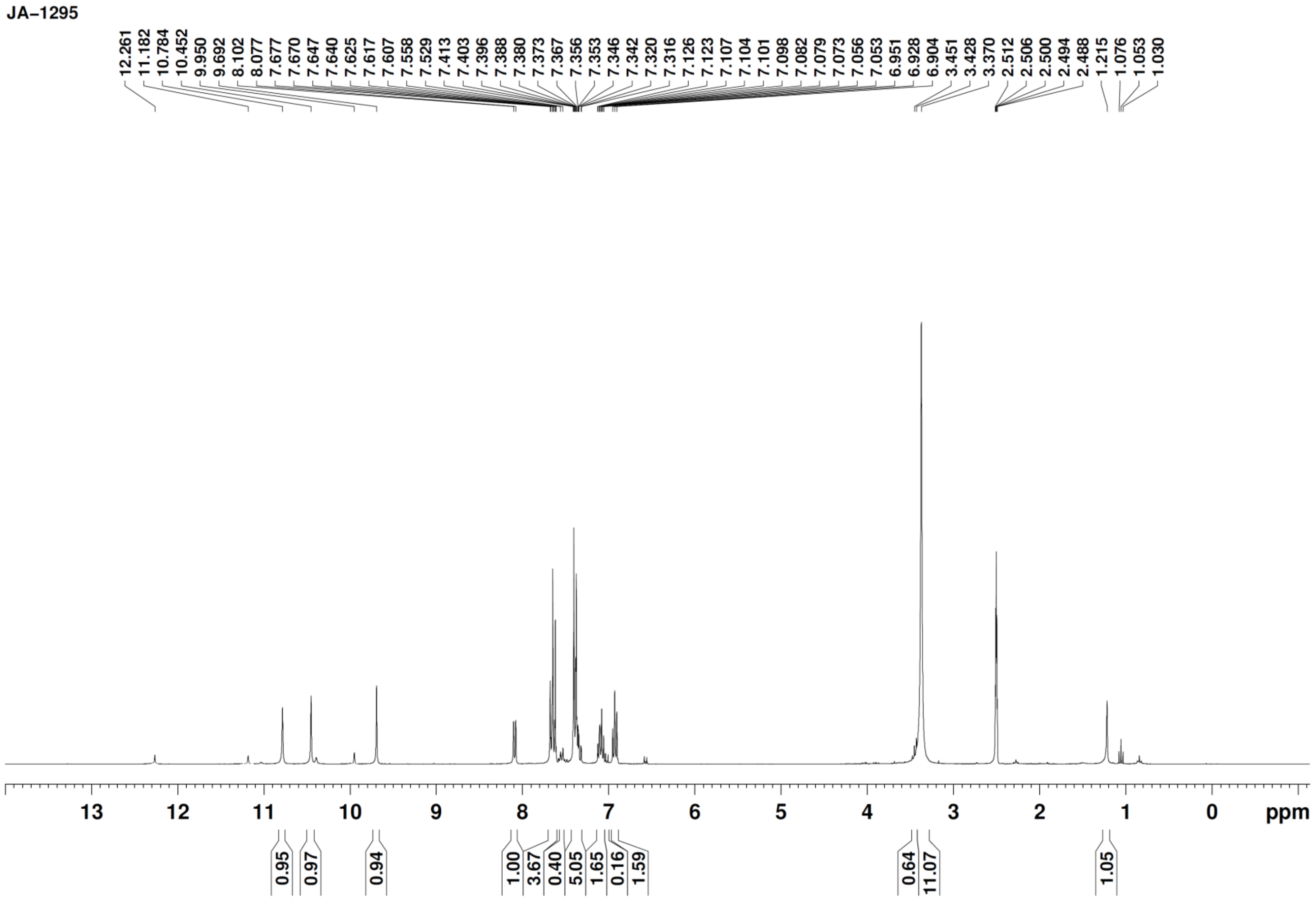

^{1}H NMR spectra of Compound **6b**

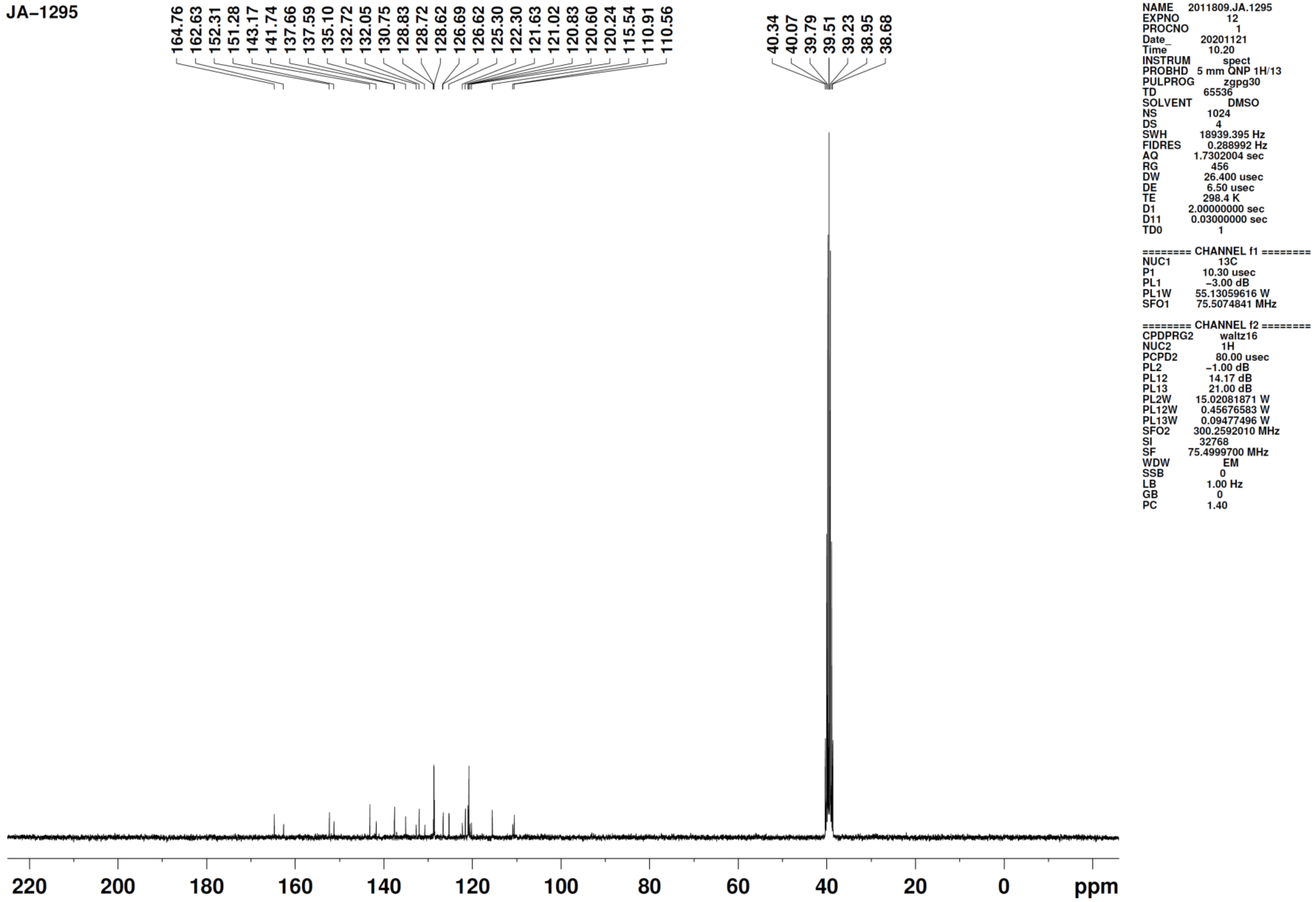

^{13}C NMR spectra of Compound **6b**

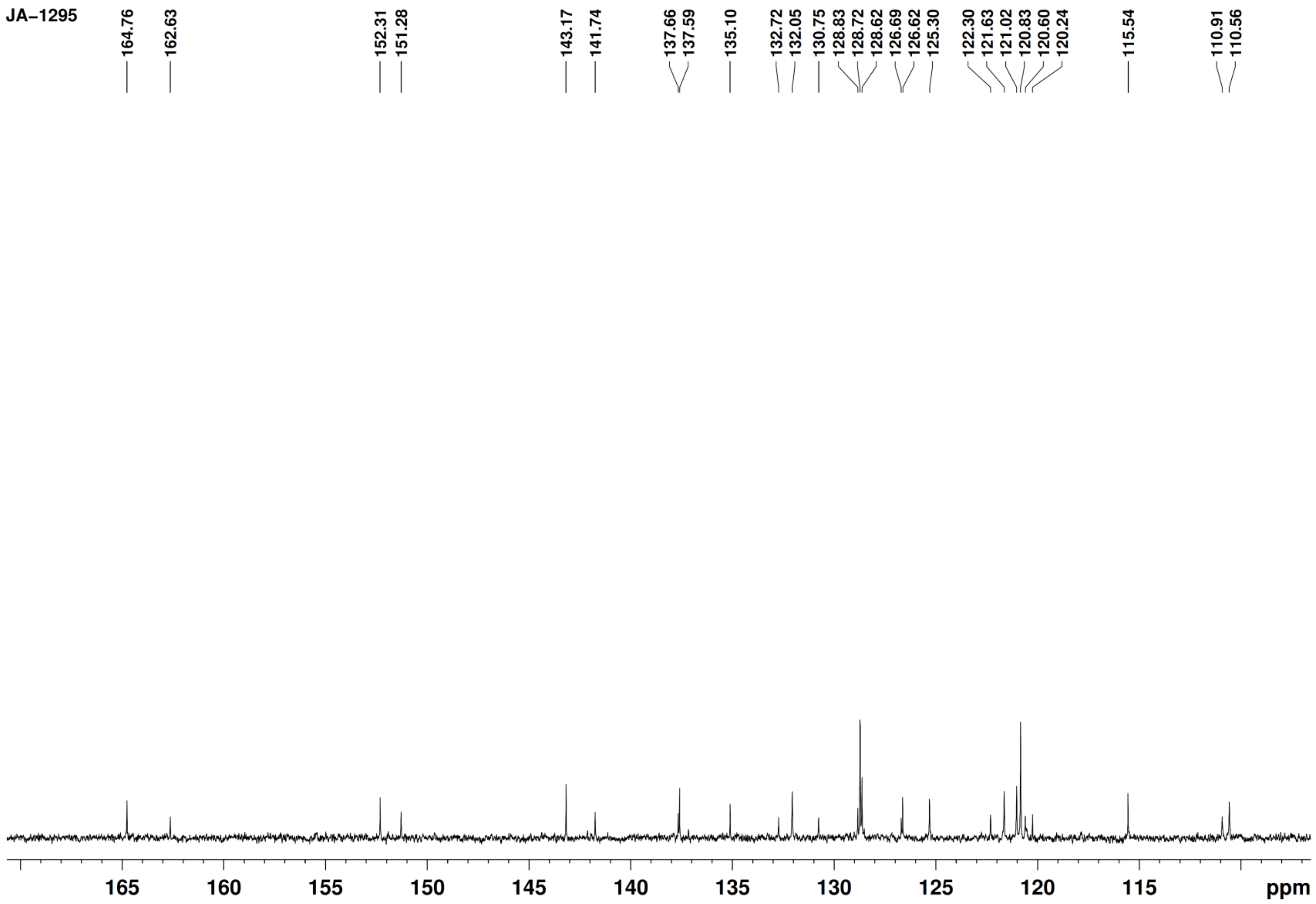

^{13}C NMR spectra of Compound **6b** showing multiplicity in peaks

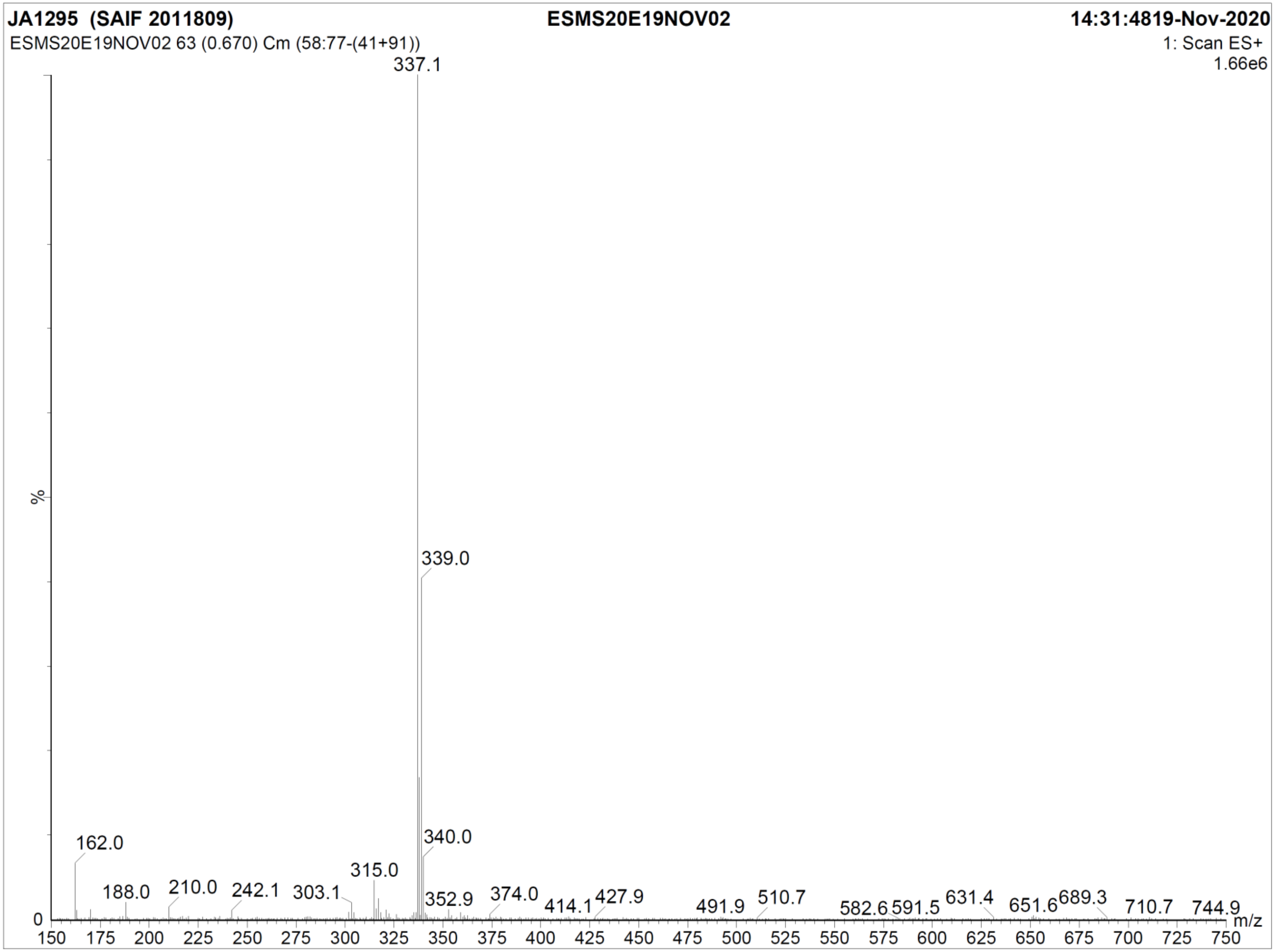

Mass spectra of Compound **6b**

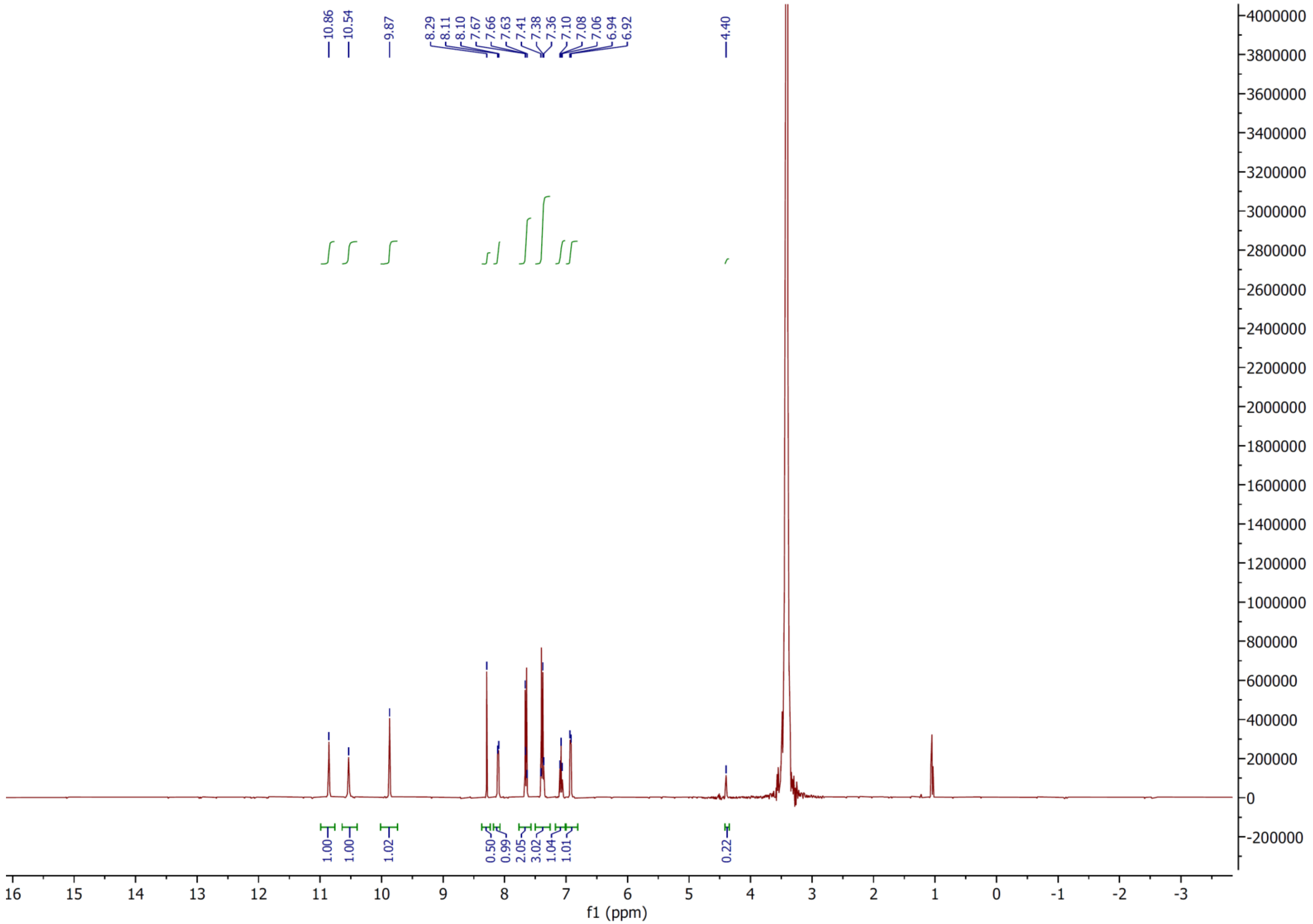

^{1}H NMR spectra of Compound **6c**

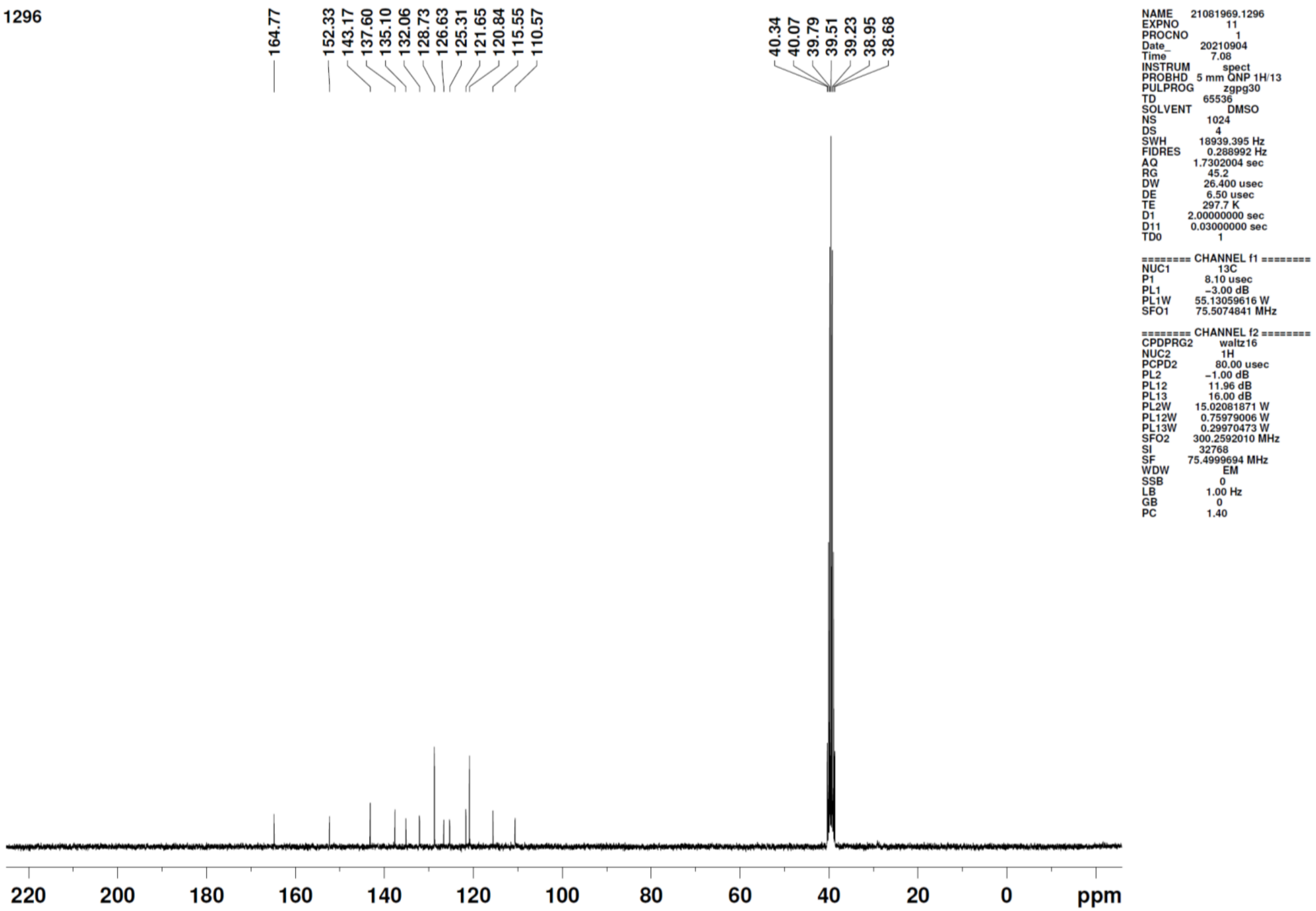

^{13}C NMR spectra of Compound **6c**

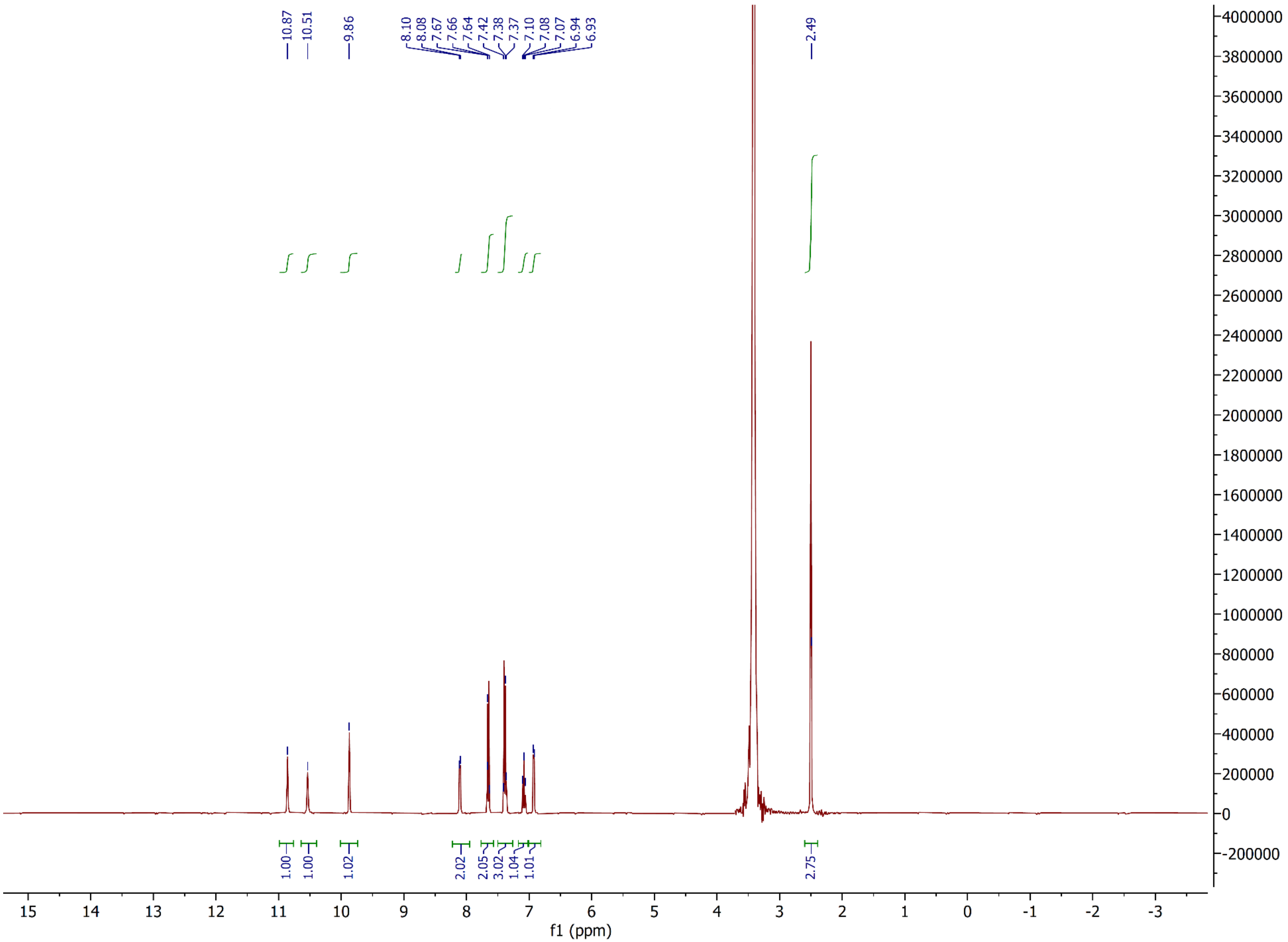

[1] H NMR spectra of Compound **6e**

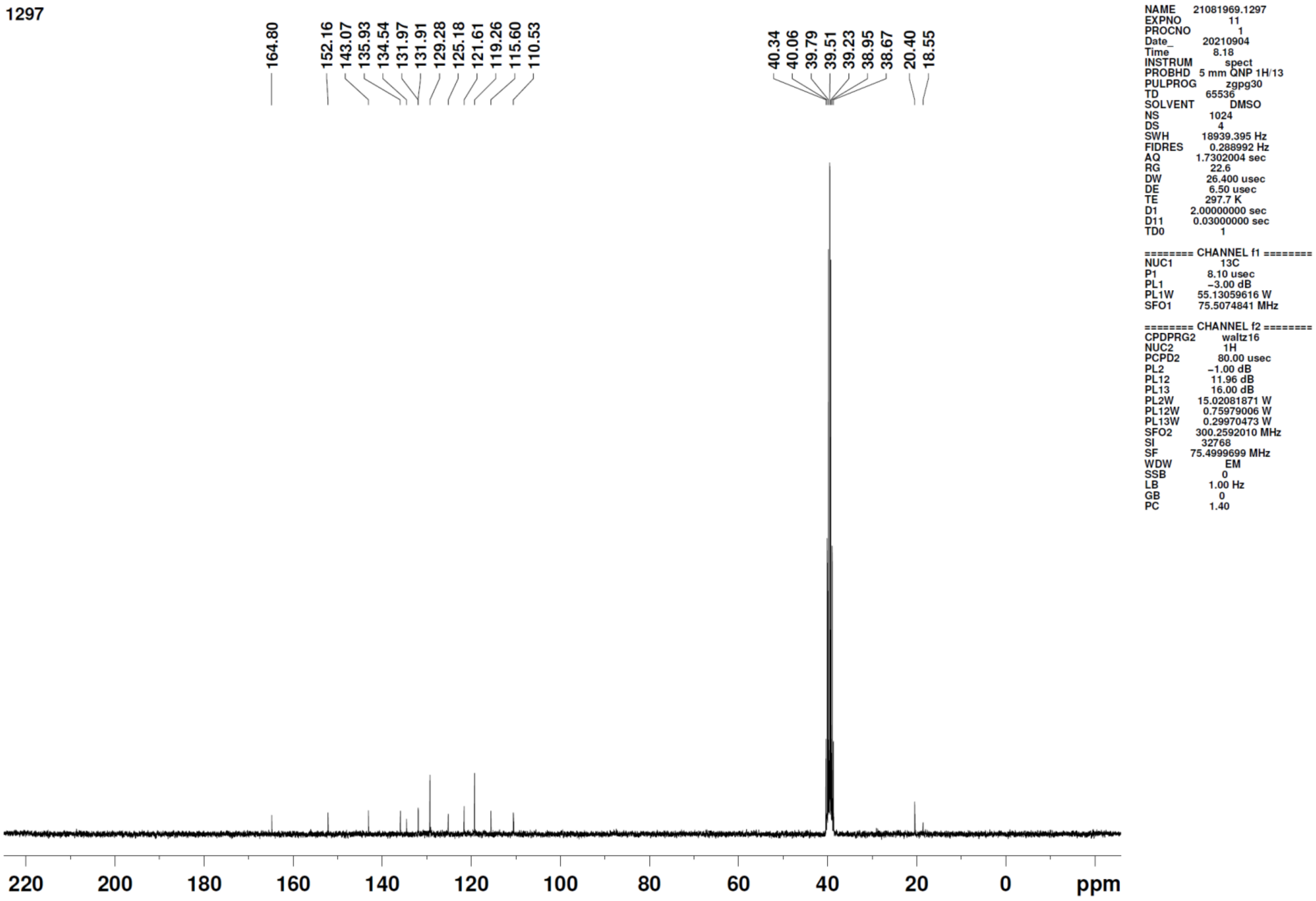

^{13}C NMR spectra of Compound **6e**

Sample Information

Date Acquired	: 6/19/2021 3:10:14 PM
Sample Name	:
Sample ID	: 1297
Data File	: JA1297
Method File	: LALSPC +&-.lcm

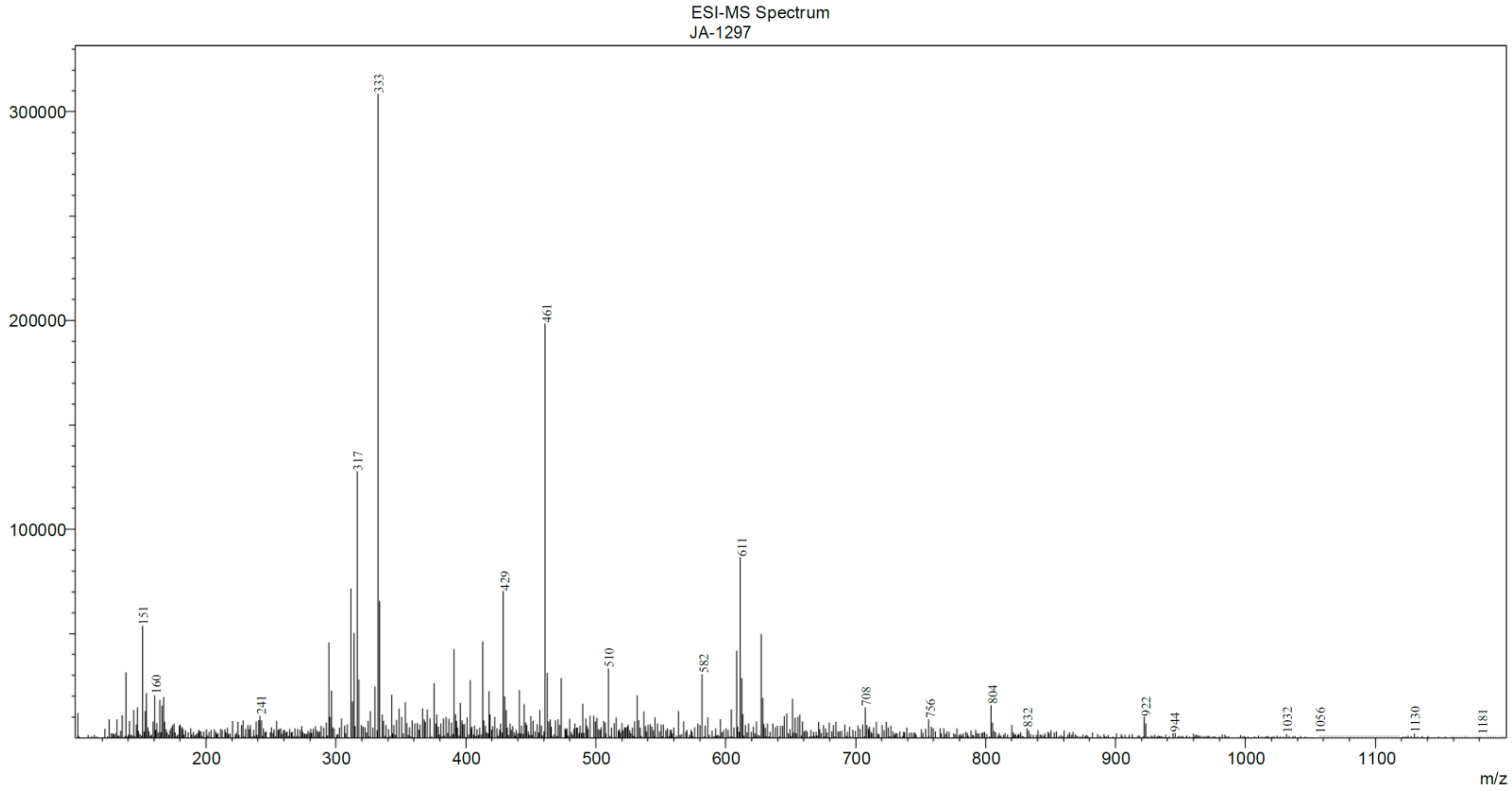

Mass spectra of Compound **6e**

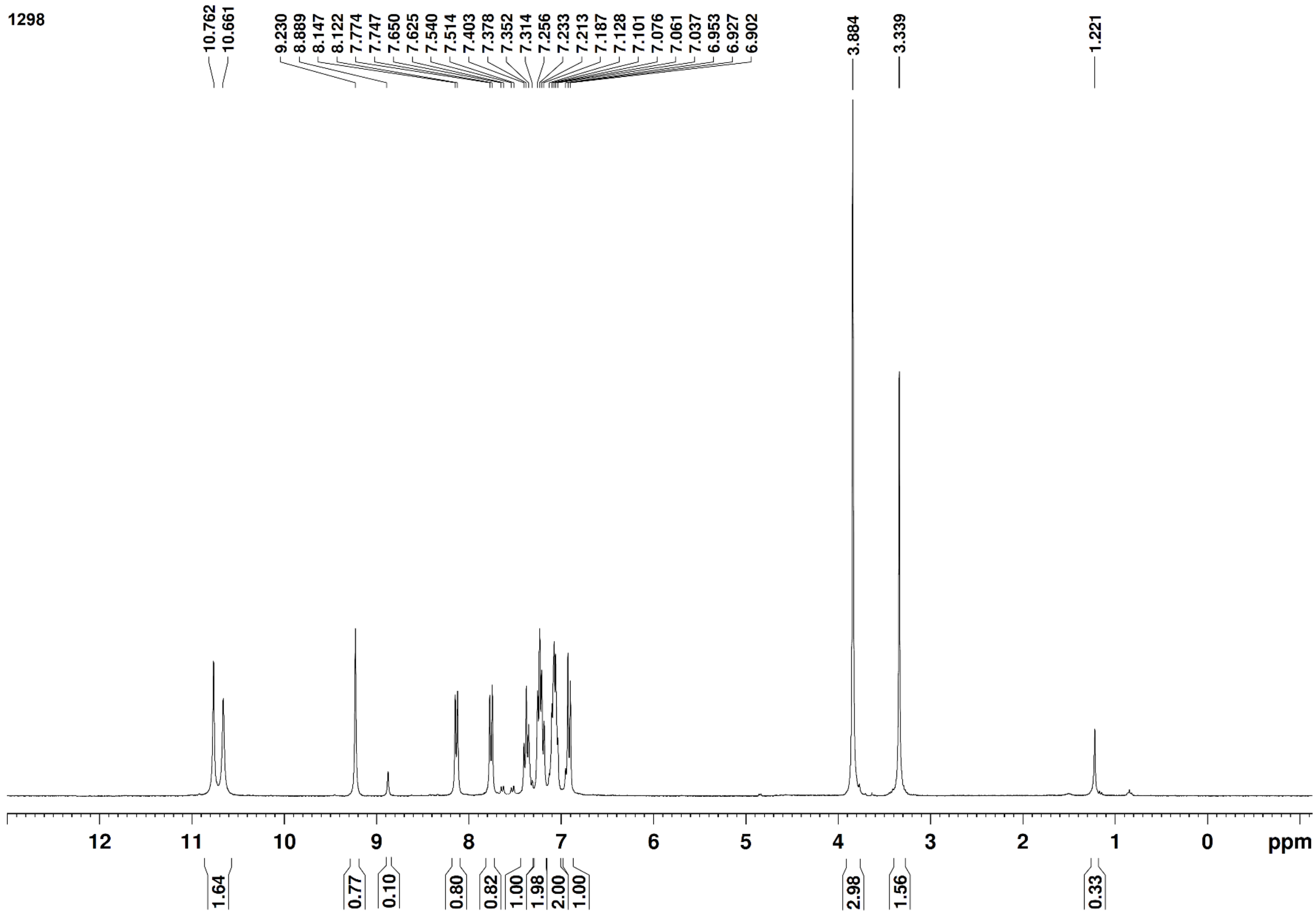

[1] H NMR spectra of Compound **6f**

Sample Information

Date Acquired	: 6/19/2021 3:14:43 PM
Sample Name	:
Sample ID	: JA-1298
Data File	: JA-1298
Method File	: LALSPC +&-.lcm

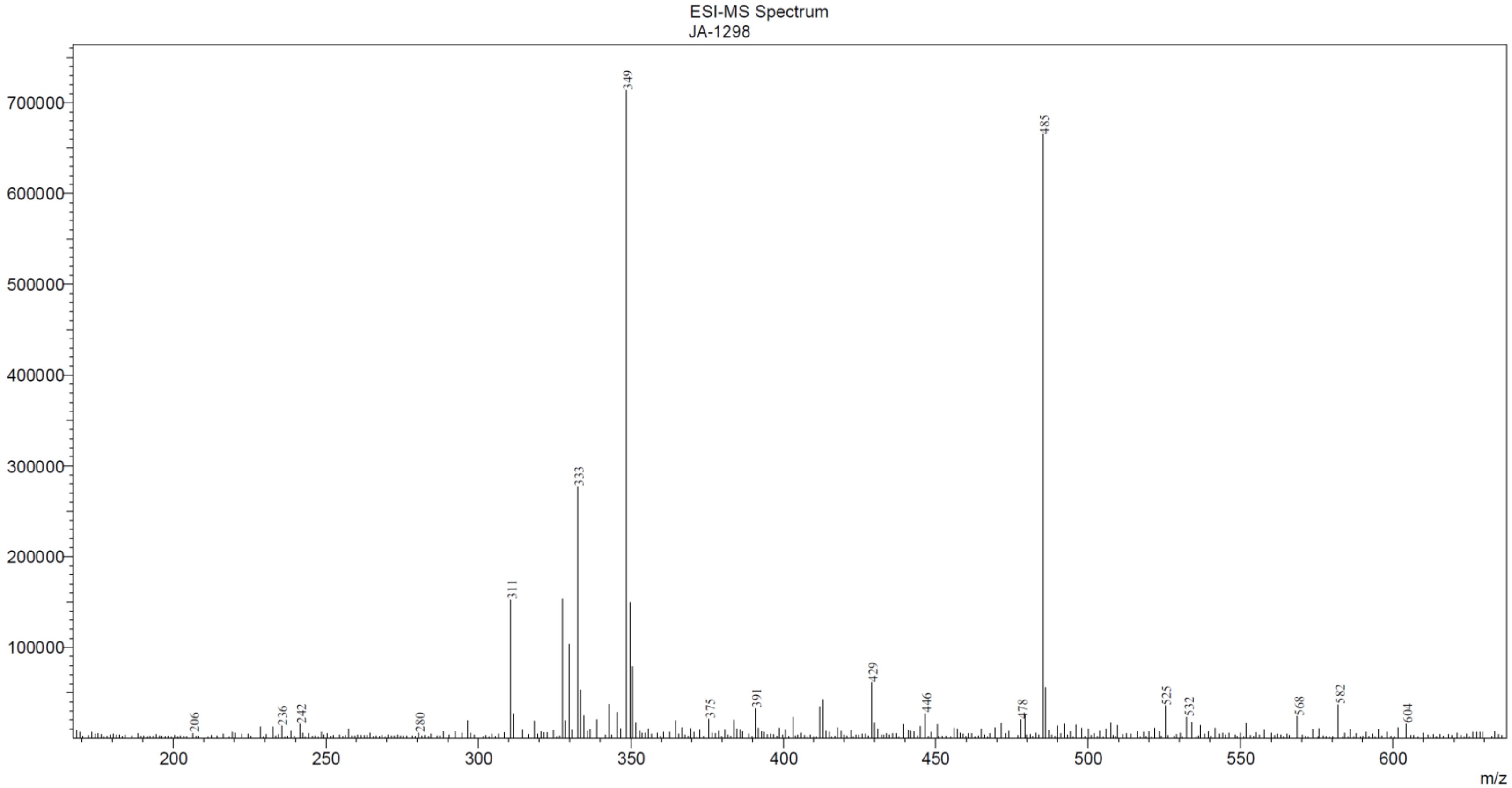

Mass spectra of Compound **6f**

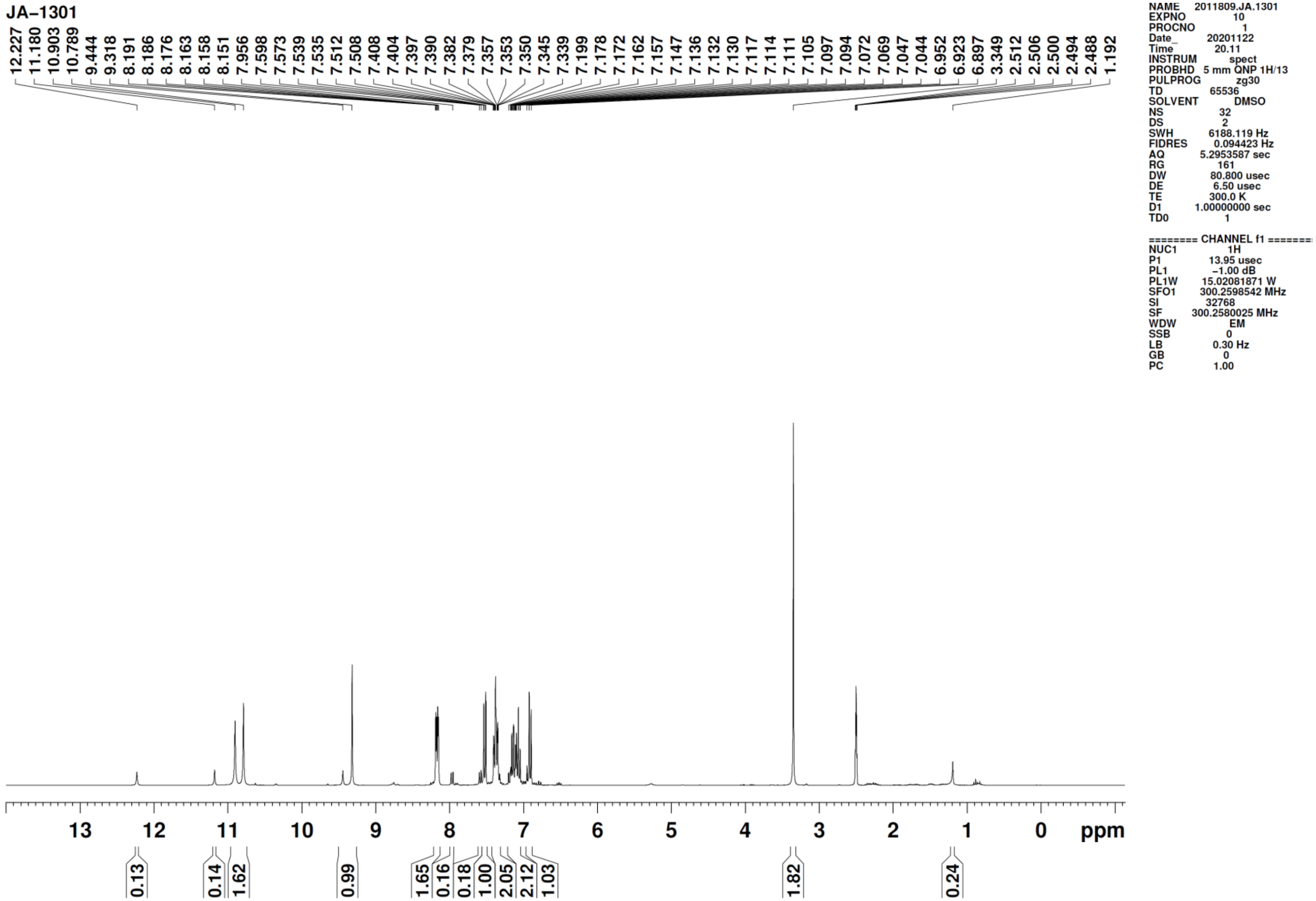

^{1}H NMR spectra of Compound **6g**

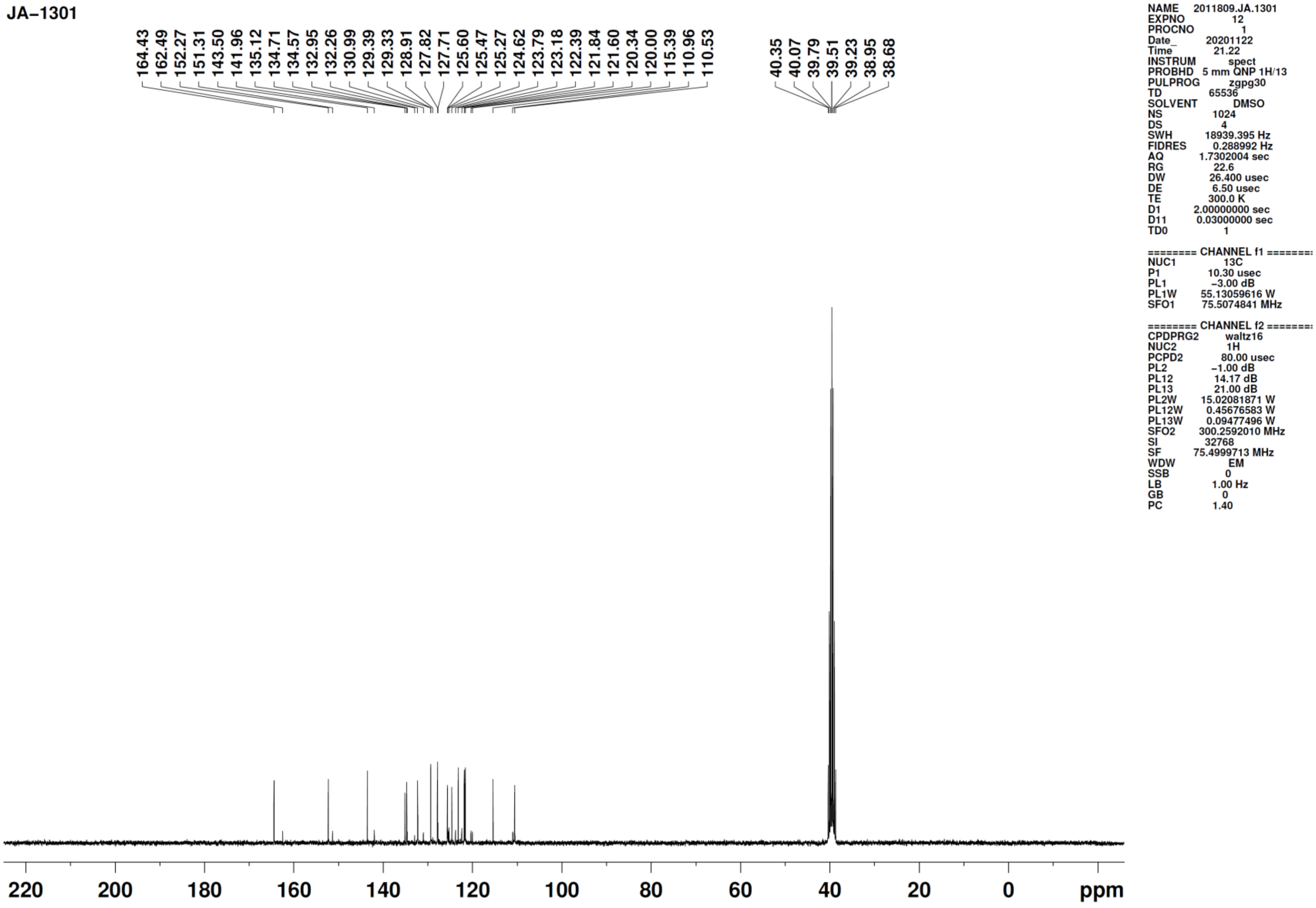

^{13}C NMR spectra of Compound **6g**

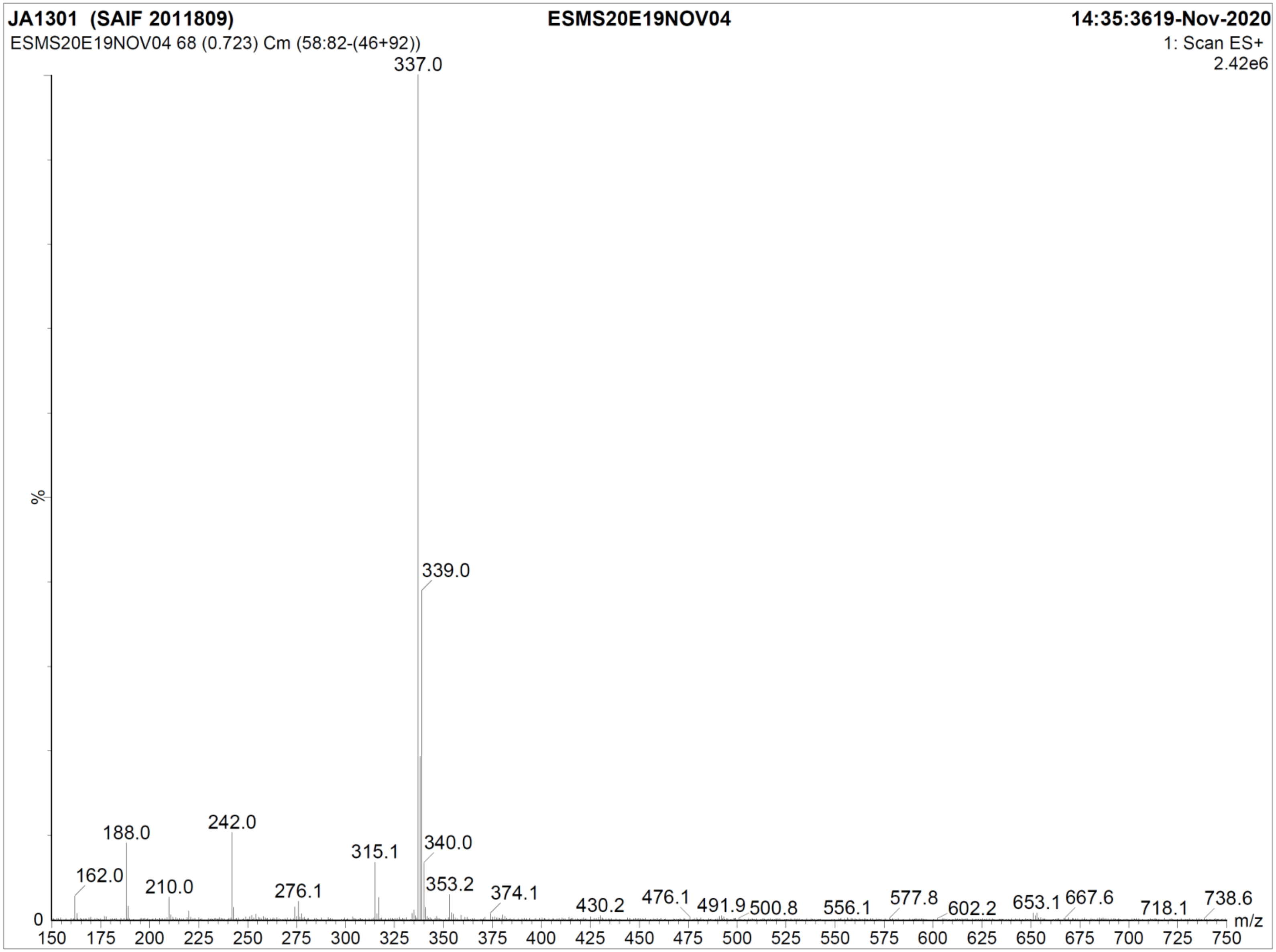

Mass spectra of Compound **6g**

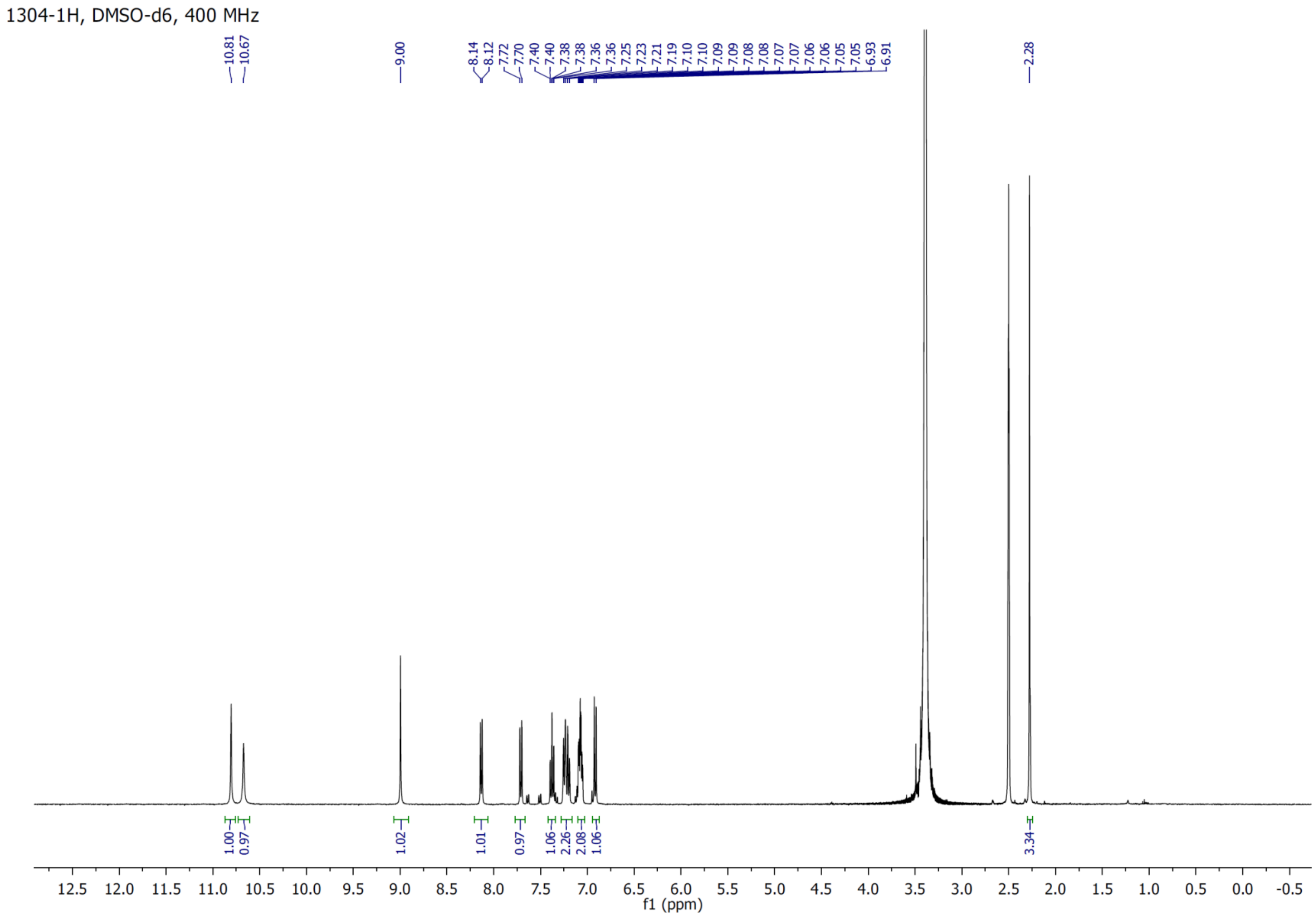

^{1}H NMR spectra of Compound **6h**

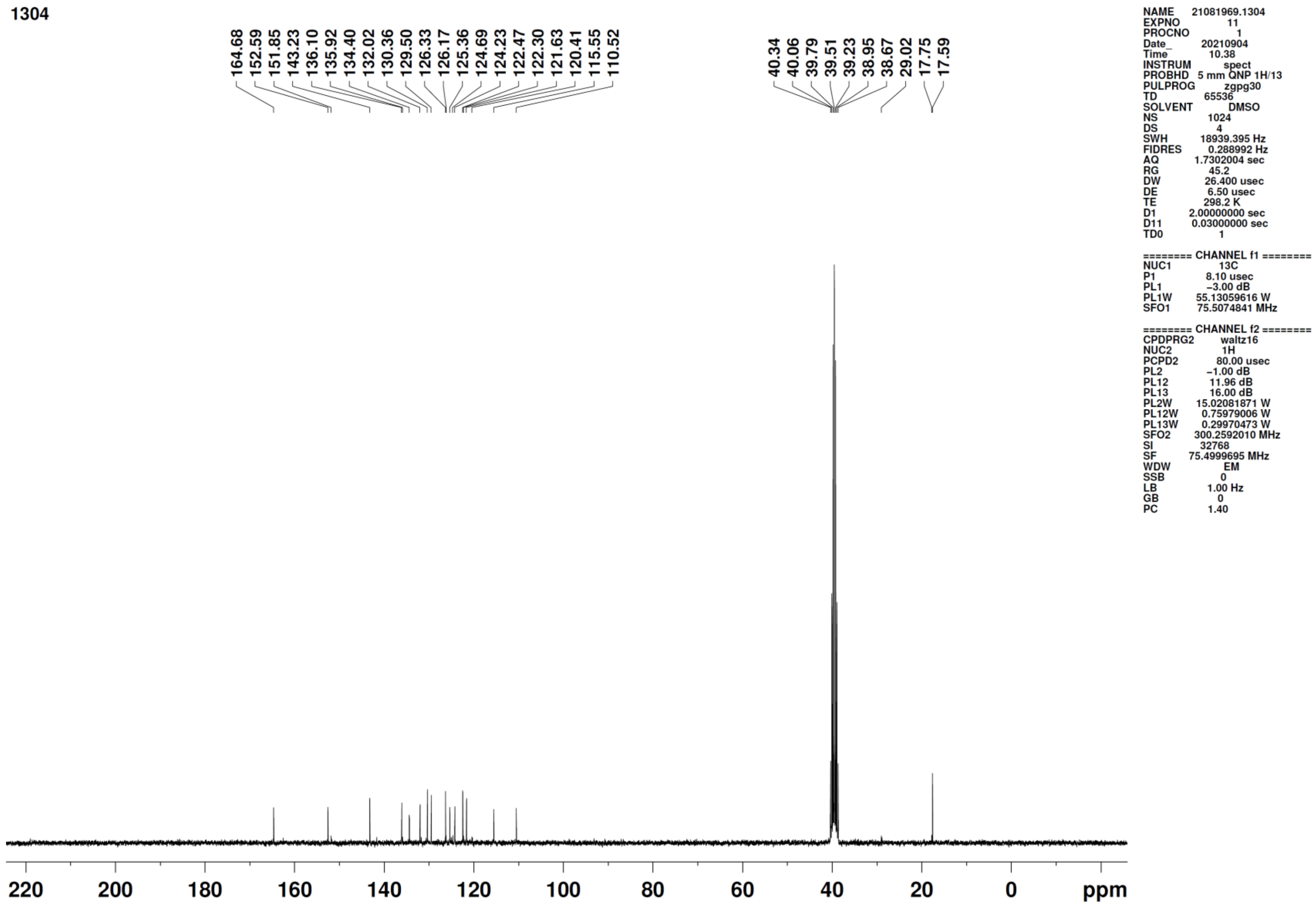

^{13}C NMR spectra of Compound **6h**

Sample Information

Date Acquired	: 6/19/2021 3:17:26 PM
Sample Name	:
Sample ID	: JA-1304
Data File	: JA-1304
Method File	: LALSPC +&-.lcm

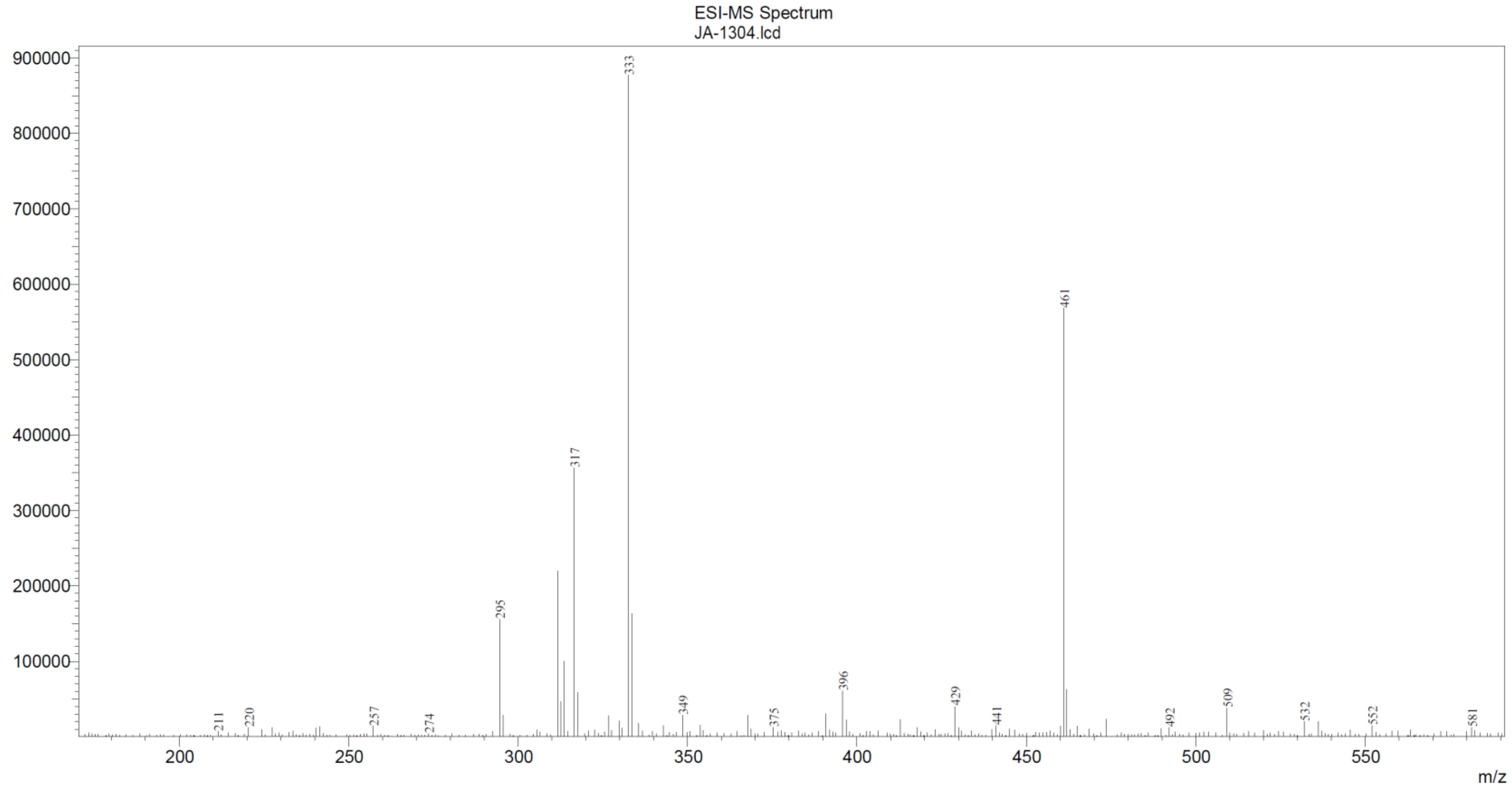

Mass spectra of Compound **6h**

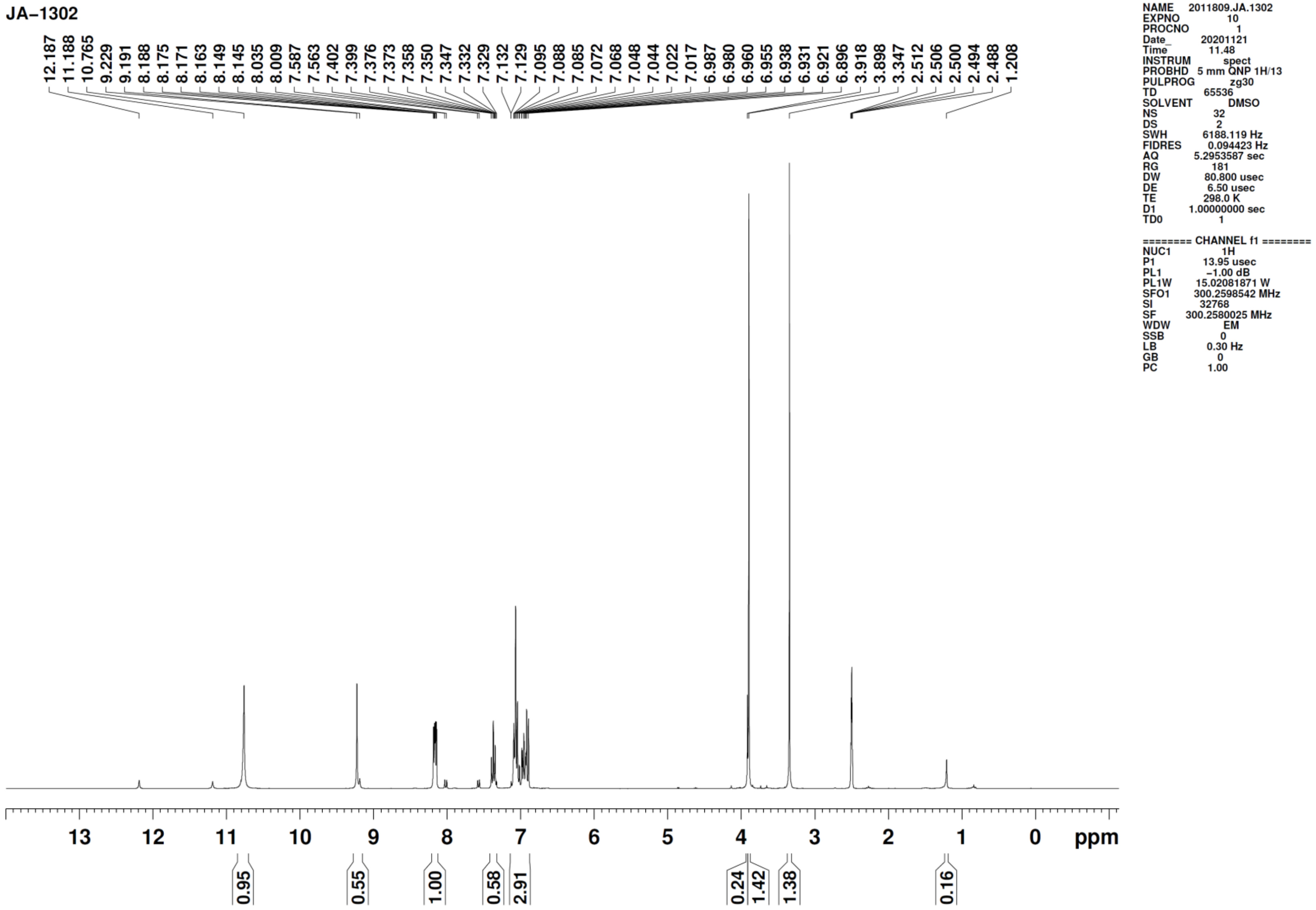

^{1}H NMR spectra of Compound **6i**

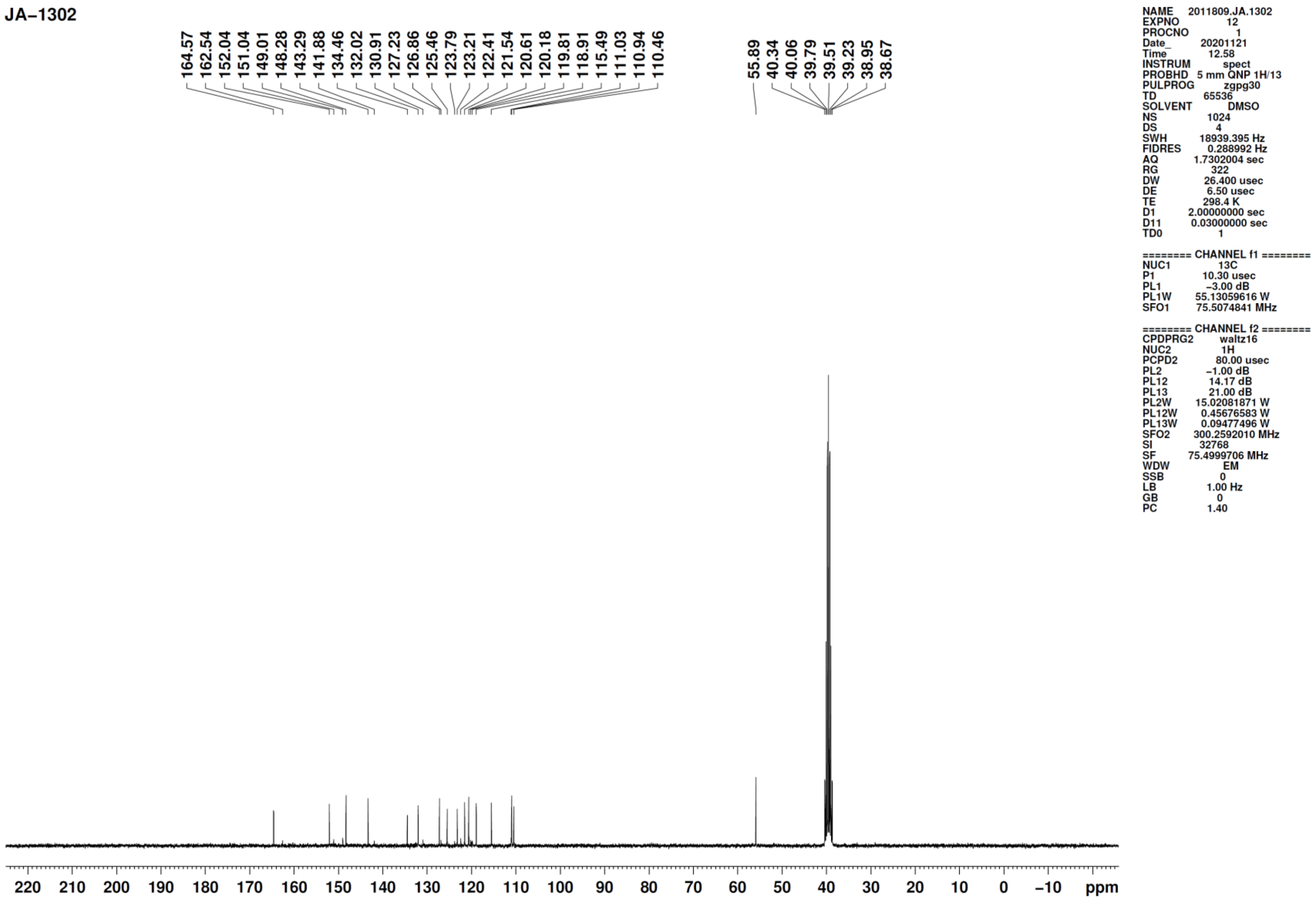

^{13}C NMR spectra of Compound **6i**

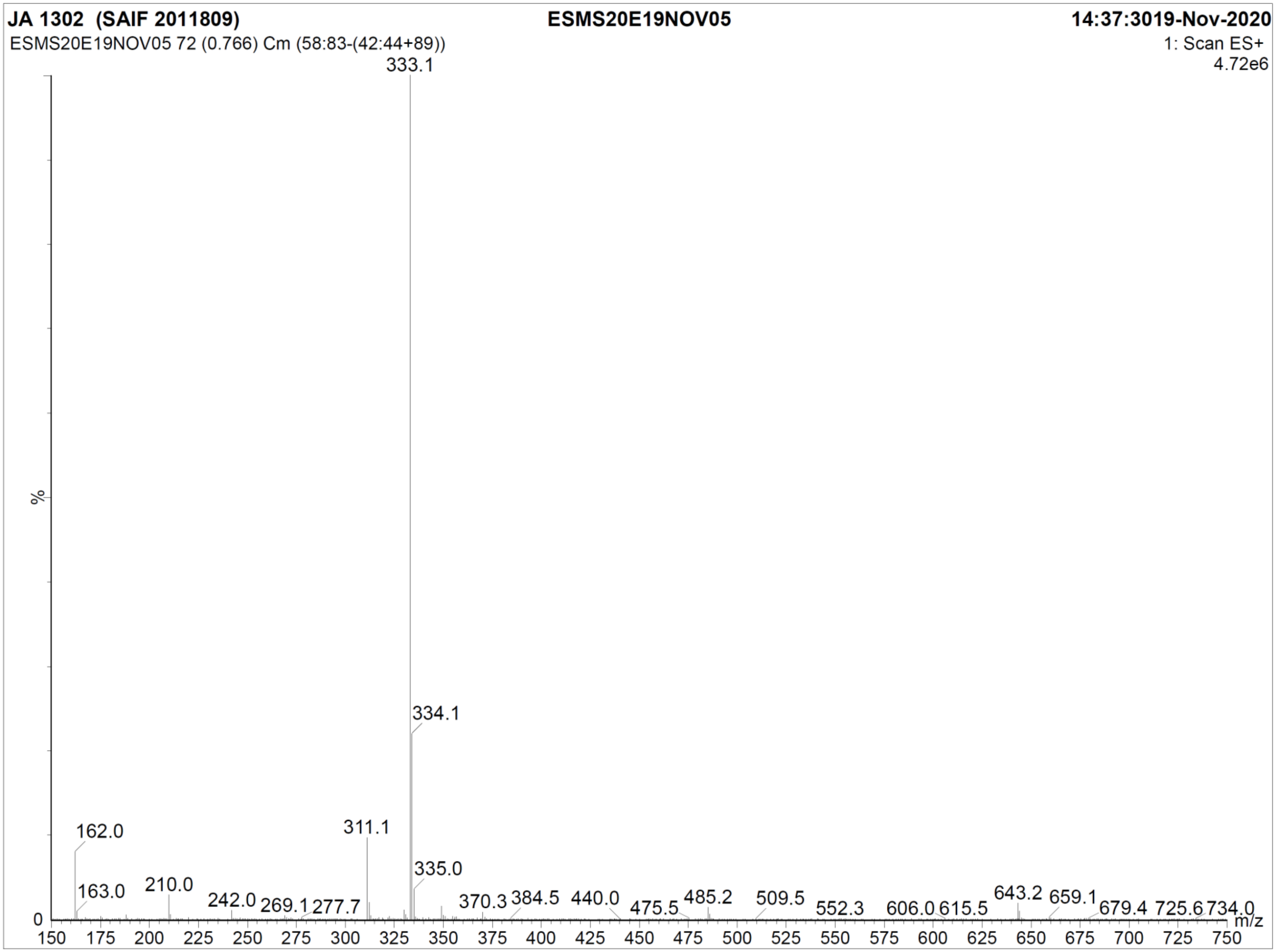

Mass spectra of Compound **6i**

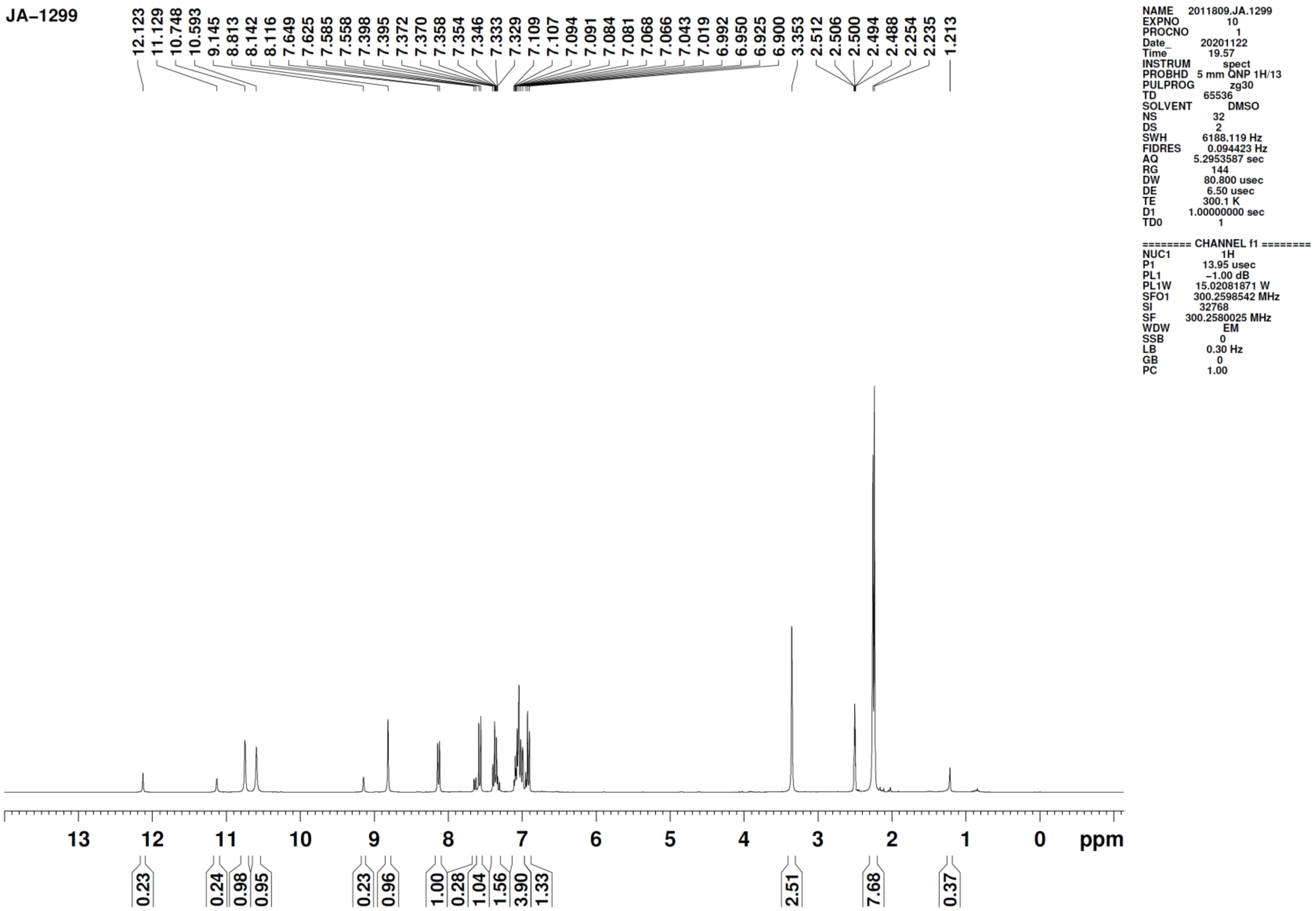

^{1}H NMR spectra of Compound **6j**

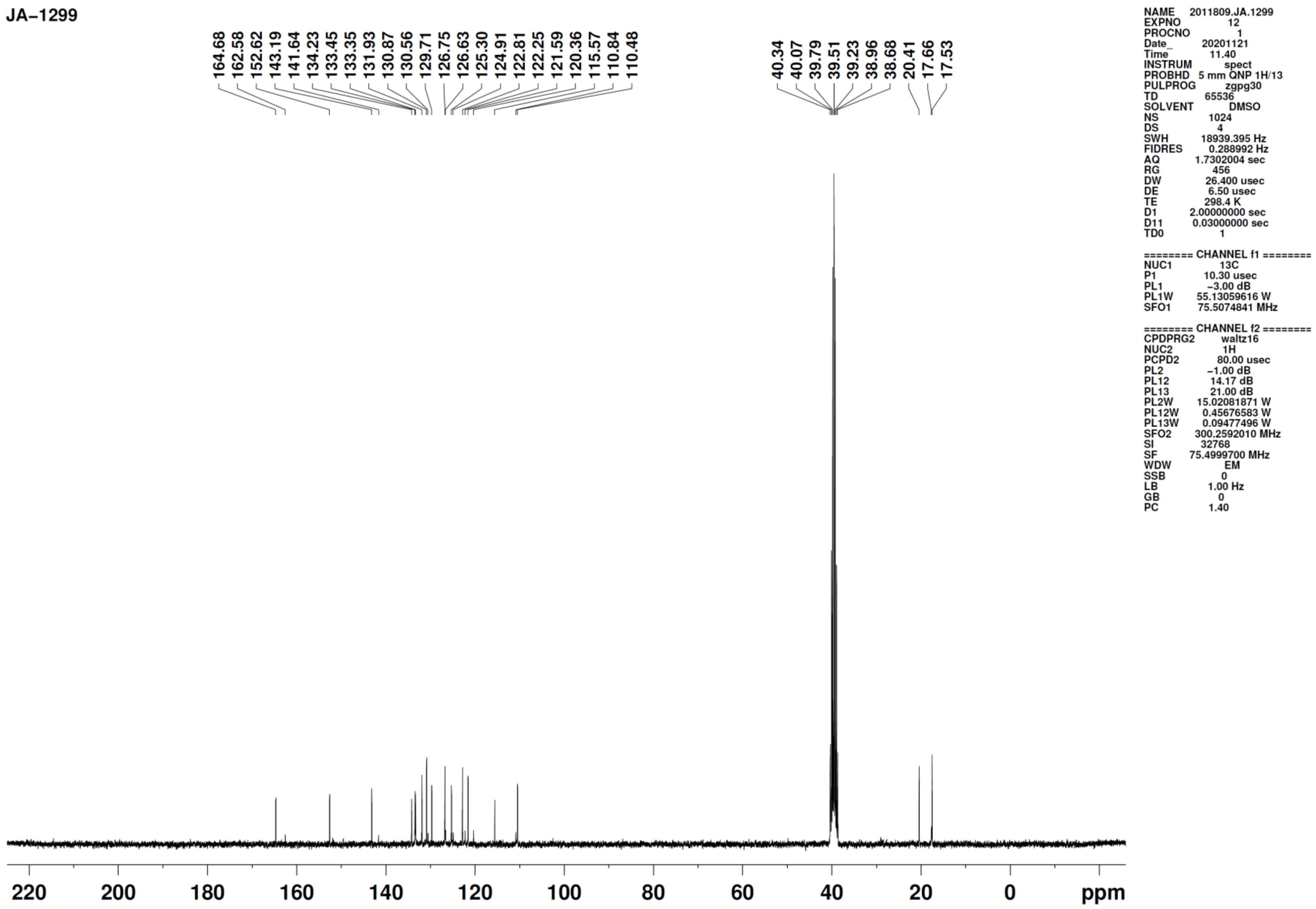

^{13}C NMR spectra of Compound **6j**

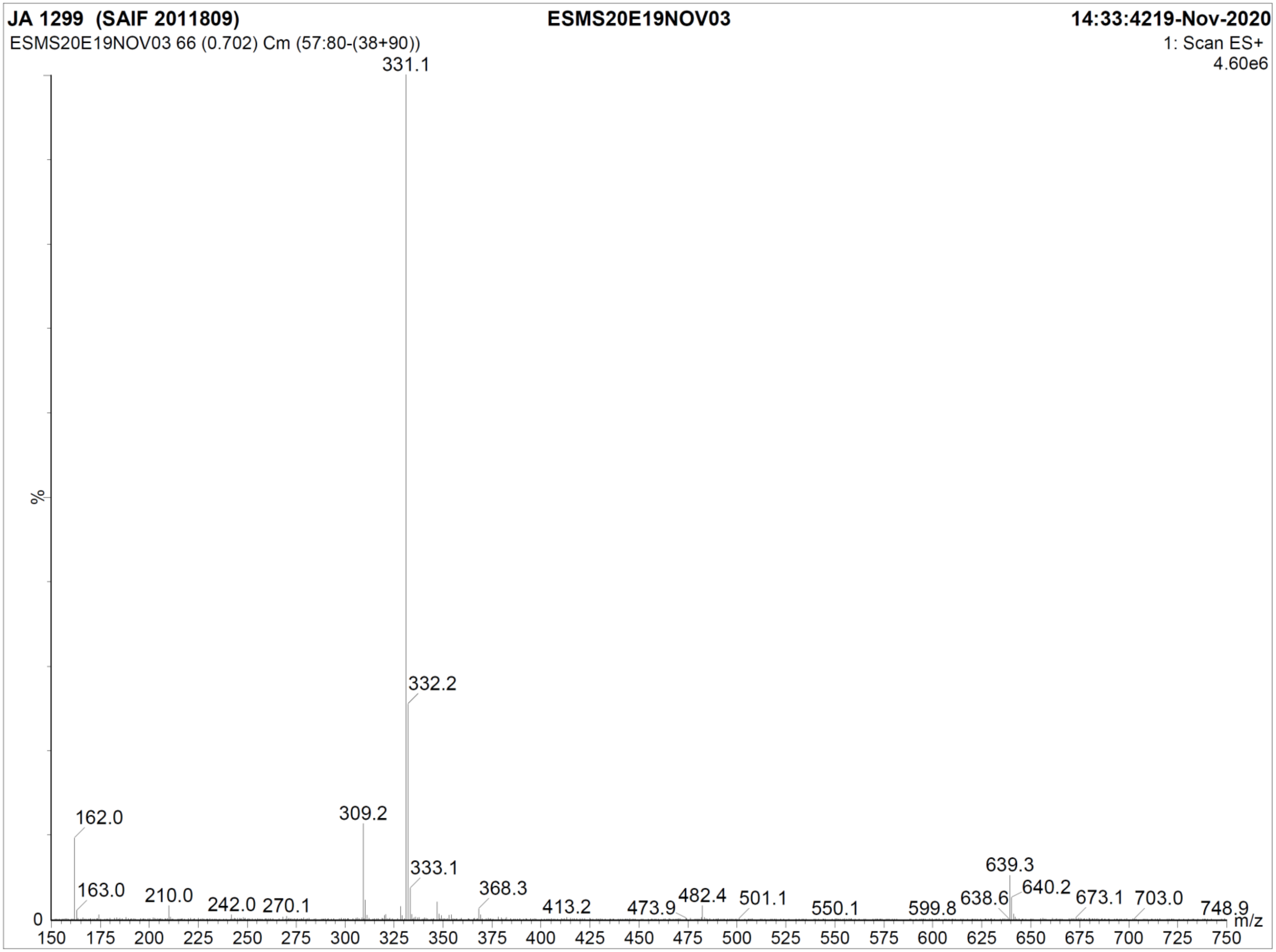

Mass spectra of Compound **6j**

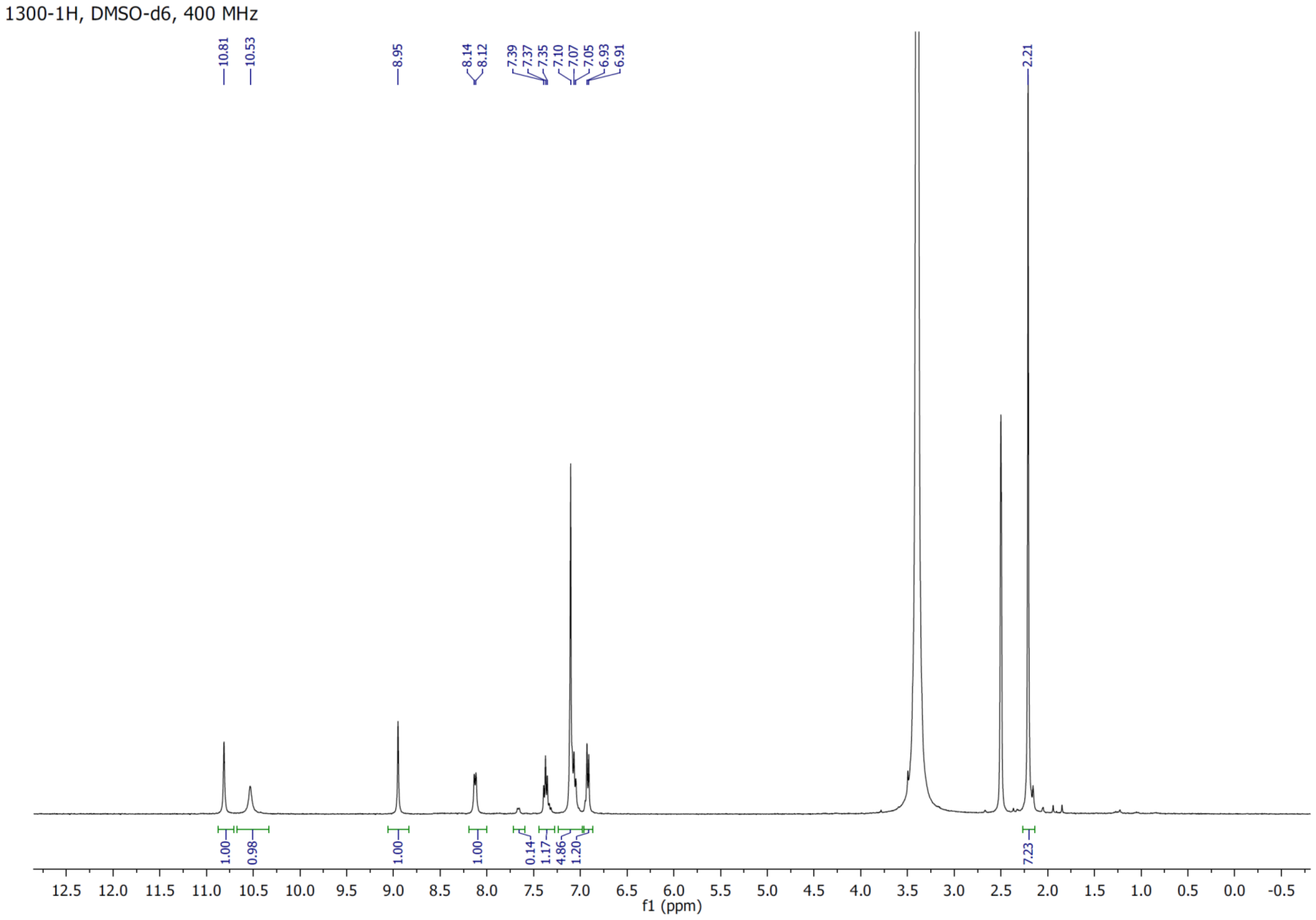

^{1}H NMR spectra of Compound **6k**

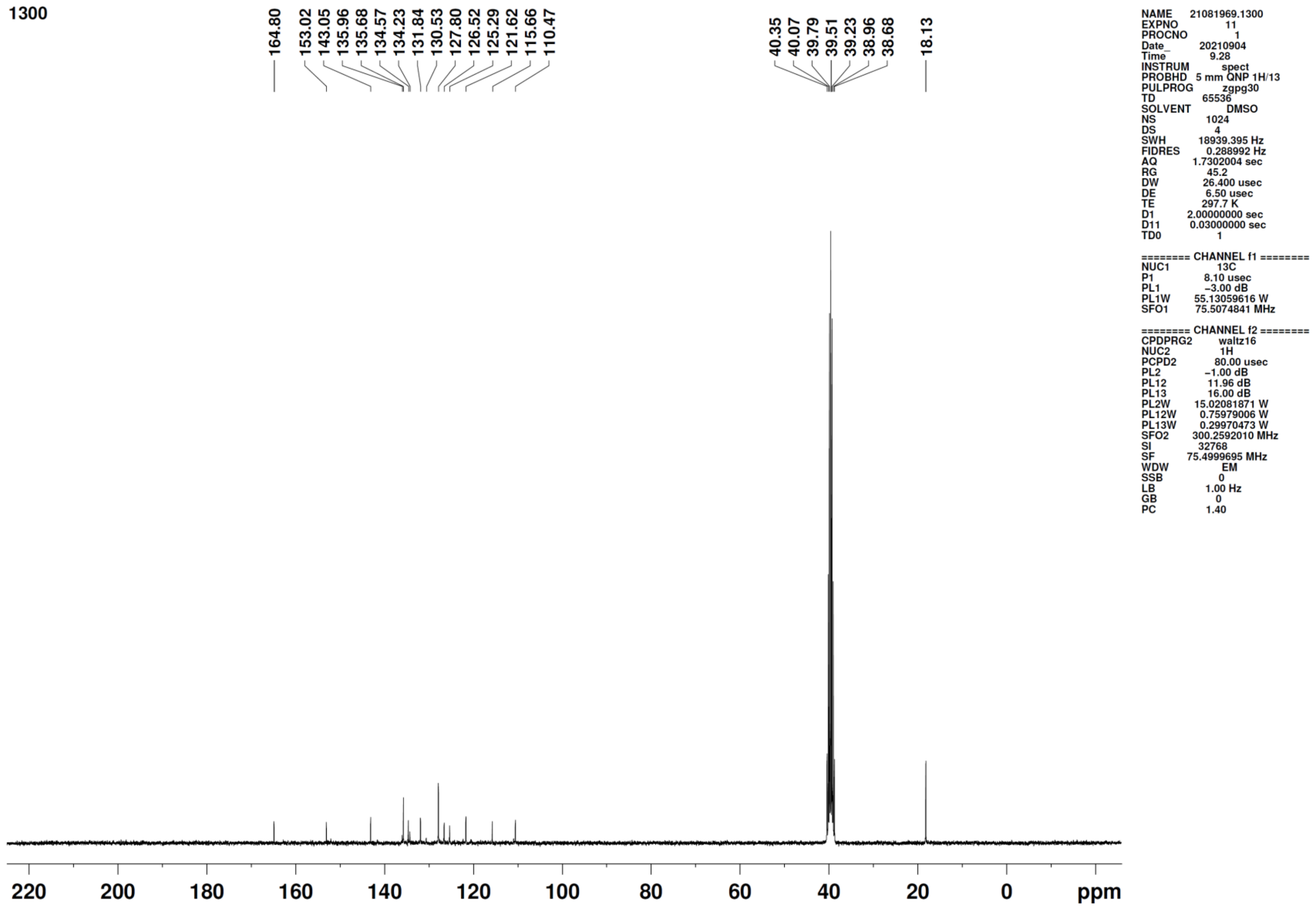

^{13}C NMR spectra of Compound **6k**

Date Acquired : 6/19/2021 3:11:57 PM
Sample Name :
Sample ID : JA-1300
Data File : JA-1300.lcd
Method File : LALSPC +&-.lcm

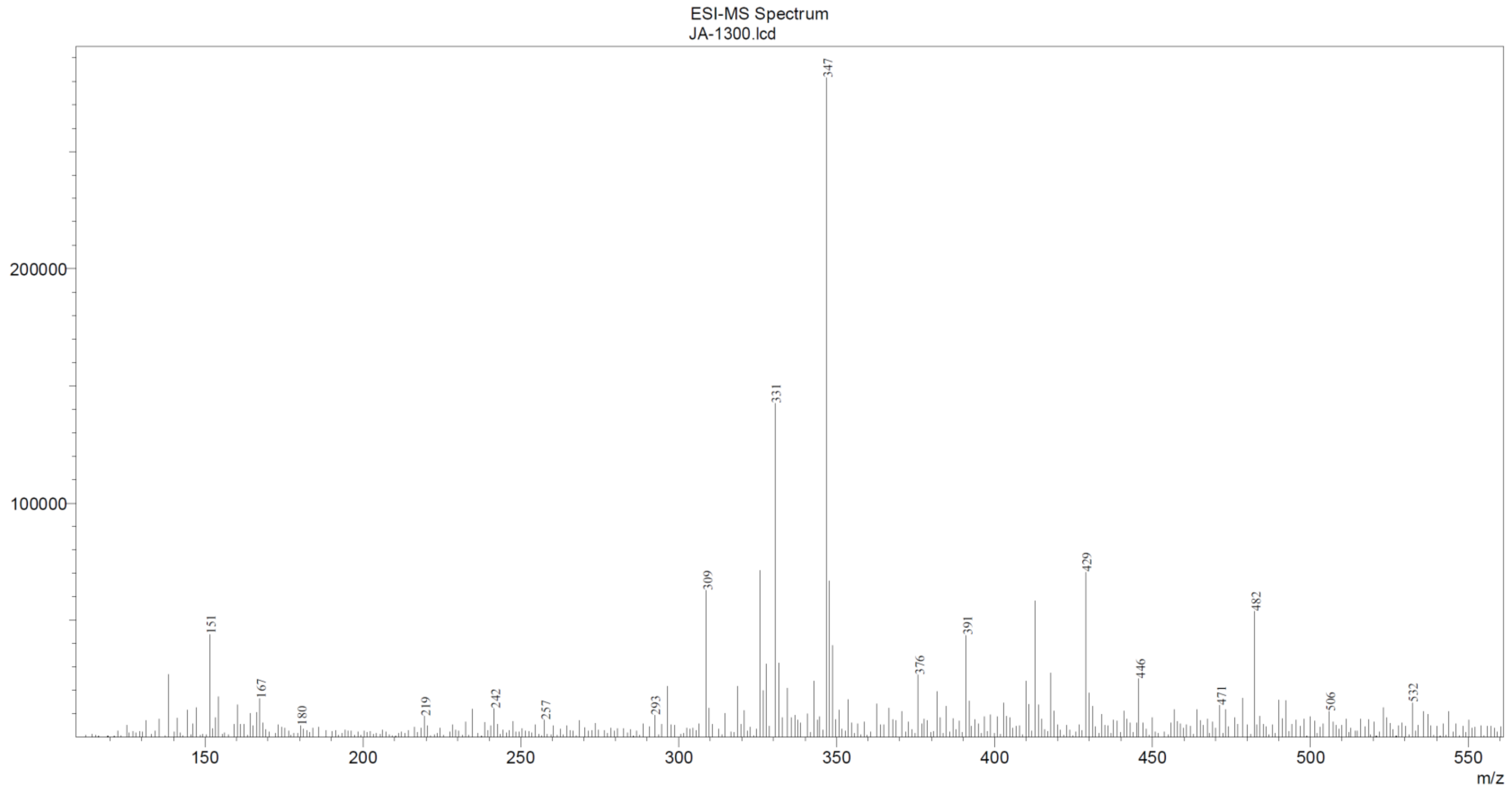

Mass spectra of Compound **6k**

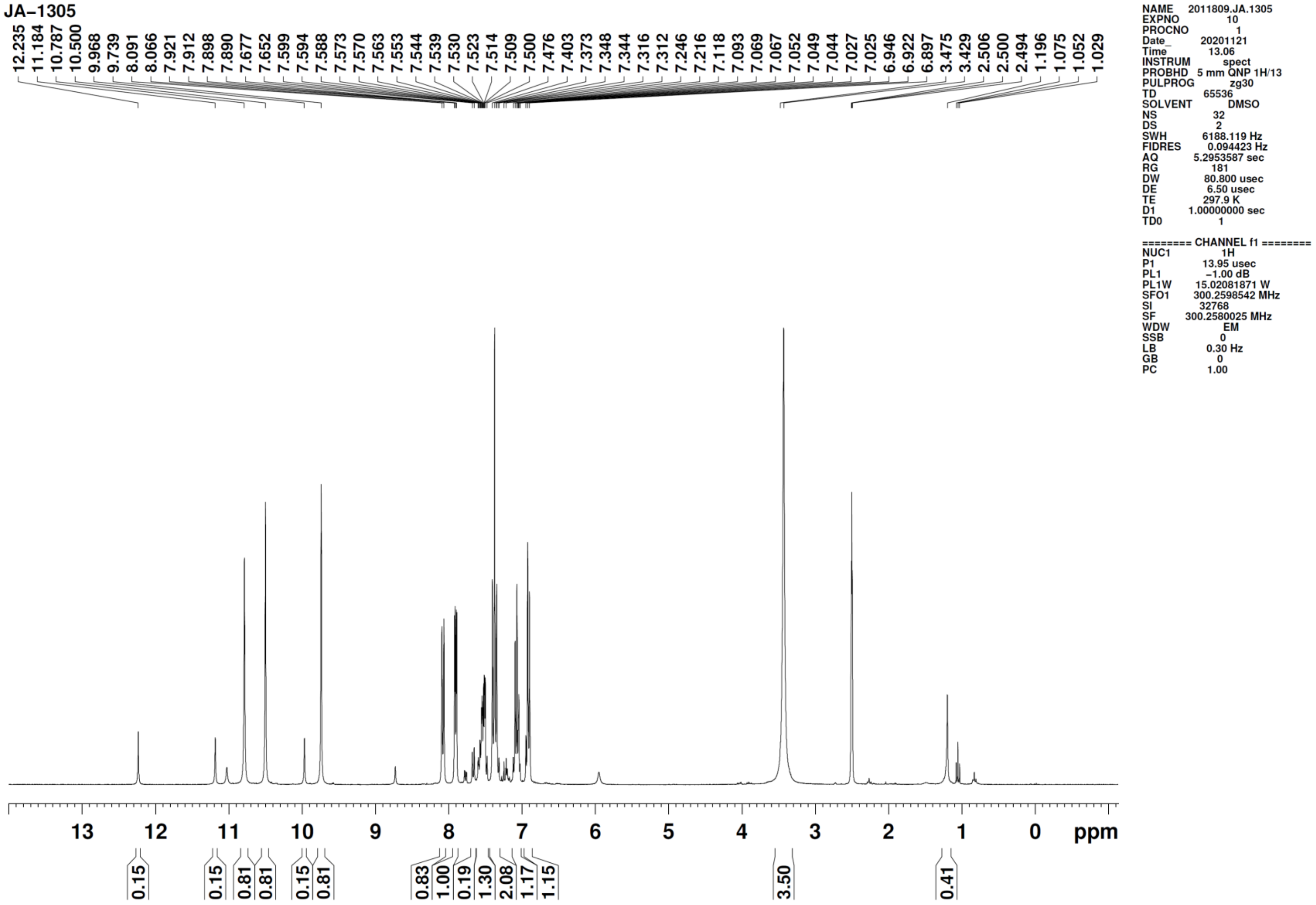

^{1}H NMR spectra of Compound **6l**

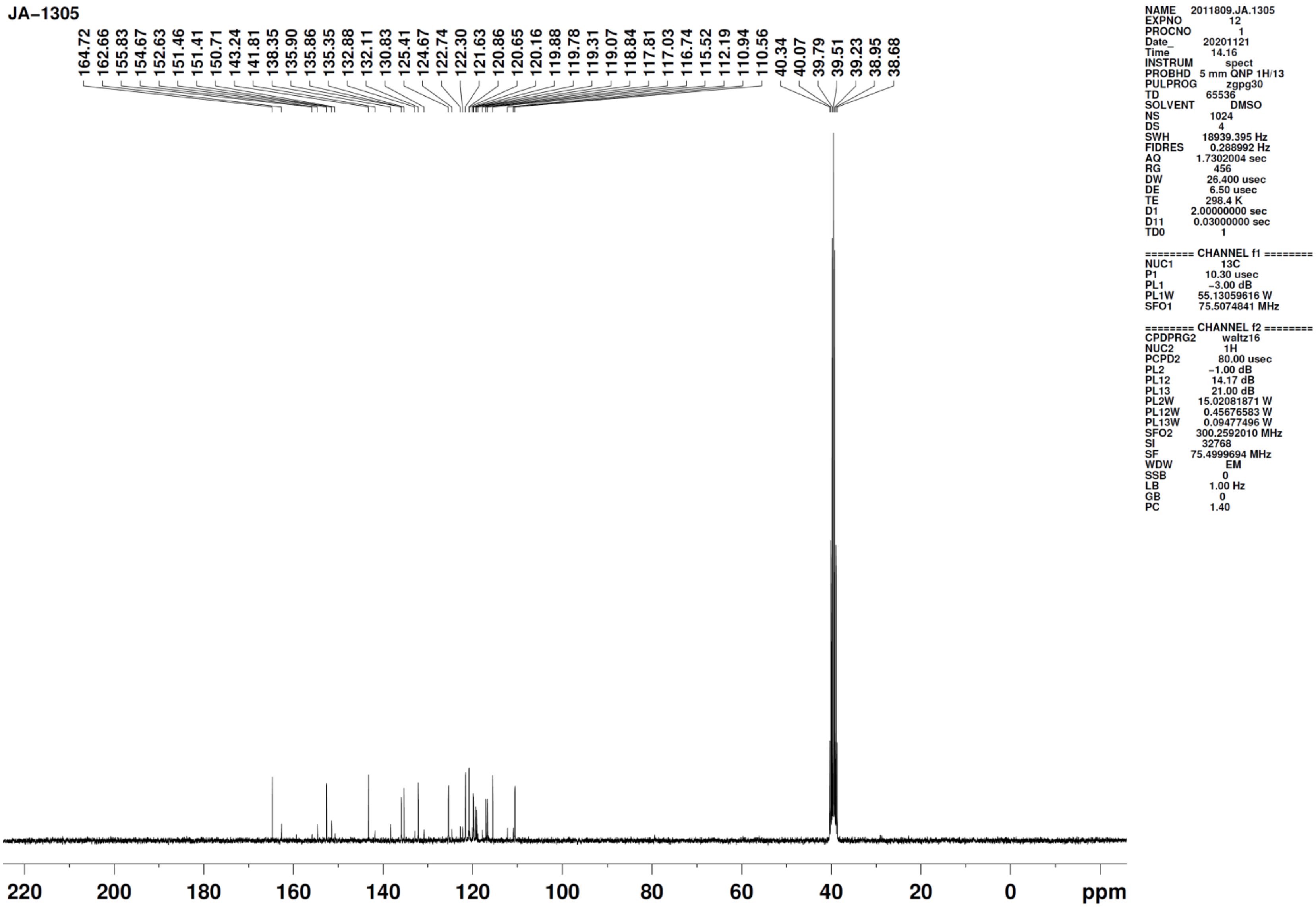

^{13}C NMR spectra of Compound **6l**

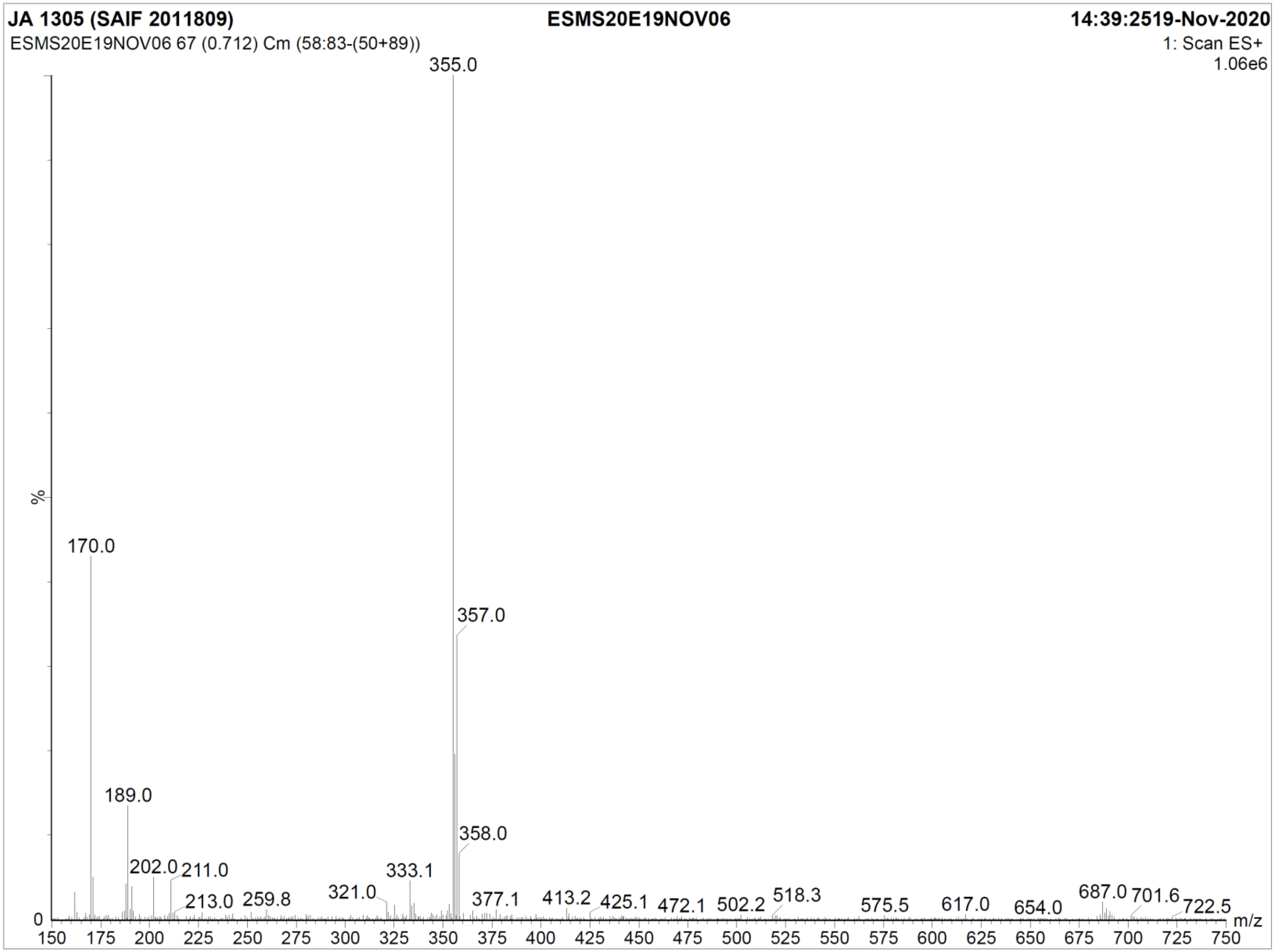

Mass spectra of Compound **6l**
